# One-Pot Regiodirected Annulations for the Rapid Synthesis of π-Extended Oligomers

**DOI:** 10.1021/acs.orglett.0c01043

**Published:** 2020-04-07

**Authors:** Andrea Nitti, Peshawa Osw, Giuseppe Calcagno, Chiara Botta, Samuel I. Etkind, Gabriele Bianchi, Riccardo Po, Timothy M. Swager, Dario Pasini

**Affiliations:** † Department of Chemistry, University of Pavia, Via Taramelli 12, 27100 Pavia, Italy; ‡ INSTM Research Unit, University of Pavia, Via Taramelli 12, 27100 Pavia, Italy; § Department of Chemistry, College of Science, Salahaddin University, 44001 Erbil, Kurdistan Iraq; ∥ Istituto per lo Studio delle Macromolecole (ISMAC), CNR, Via Corti 12, 20133 Milano, Italy; ⊥ Department of Chemistry, Massachusetts Institute of Technology, Cambridge, Massachusetts 02139, United States; # Research Center for Renewable Energies and Environment, Istituto Donegani, Eni Spa, Via Fauser 4, 28100 Novara, Italy

## Abstract

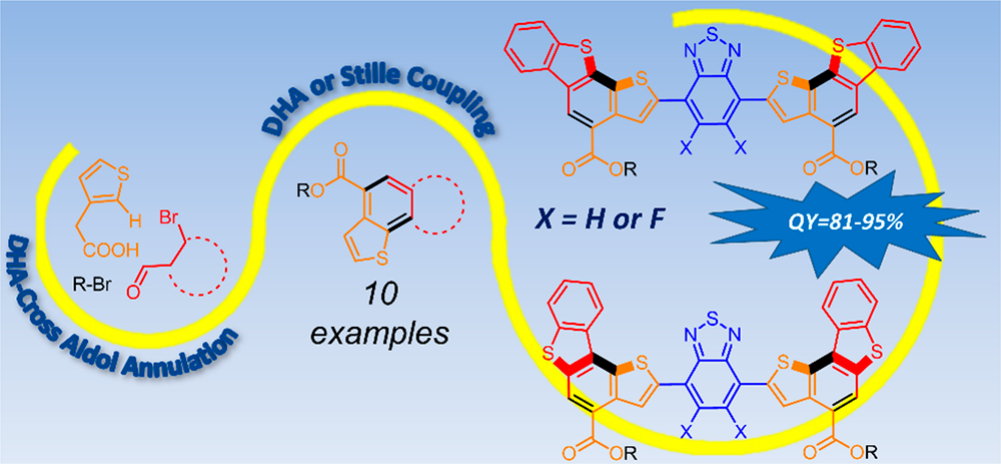

We demonstrate the broad applicability of the annulation protocol combining, in one pot, a direct arylation and cross aldol condensation for the straightforward synthesis at gram-scale of π-extended thiophene-based scaffolds. The regiospecific direct arylation drives the subsequent cross-aldol condensation proceed under the same basic conditions, and the overall protocol has broad applicability in the synthesis of extended aromatics wherein the thiophene ring is annulated with furans, pyridines, indoles, benzothiophenes, and benzofurans. These scaffolds can be further elaborated into π-extended, highly fluorescent oligomers with a central deficient benzothiadiazole unit with up to nine aromatic rings through coupling reactions.

The discovery of novel π-conjugated molecules is of foremost importance for the continued development of organic semiconductors for applications in solid-state devices.^1^ Although conjugated polymers are the current choice for solid state organic devices as a consequence of their film forming and mechanical properties,^2^ the use of oligomers has been pushed forward recently. Conjugated oligomers can share some of the electronic properties of conjugated polymers, and additionally present advantages over polymers, including their well-defined structure, easier purification procedures, and lack of chemical defects. Chemical modularity in the structures of such oligomeric architectures allows the fine-tuning of absorbance, emission, and HOMO/LUMO levels.^3^

Despite their enormous potential, large-scale applications of π-extended organic materials are generally hampered by the excessive costs of production, which do not yet take into account established sustainability indexes like the *E* factor (kg of organic waste/kg of product). *E* factor values for organic semiconductors are often in the excess of 10^4^, in some cases largely surpassing those for organic small molecules which are active components of pharmaceutical formulations.^4^ Our group has recently introduced a one-pot cascade methodology, comprising direct arylation^5^ (DHA) and cross-aldol condensations as the sequence of two alkaline-mediated reactions in a single process. The DHA step occurs regiospecifically on the 2-position of 3-thiopheneacetic acid and facilitates the subsequent cross aldol step, which completes the formation of an aromatic annulated ring system. This methodology is an attractive route to annulation^6^ of π-extended thiophene-based systems (Figure 1). For example, we developed a one-step syntheses of naphtho[1,2-*b*]thiophene **1** and benzo[1,2-*b*:6,5-*b′*]dithiophene **2** that has a nearly 2 orders of magnitude lower *E* factor when compared to previously reported multistep syntheses.^7^

**Figure 1:**
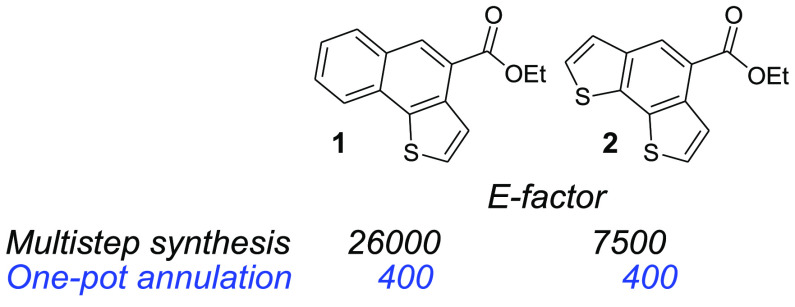
*E* factors obtained with previous multistep literature syntheses (black) and with our work (blue).

In this contribution, we report the extension of scope of the DHA-cross aldol annulation protocol to produce a library of thiophene-annulated π-molecules. The synthetic protocol is regiodirected and allows the study of regioisomers with different conjugation patterns. We were able to further elaborate the annulated products into π-extended oligomers of up to nine aromatic rings by incorporating benzo[*c*][1,2,5]thiadiazole (BT) units that have considerable utility in organic photovoltaics, solar concentrators, OLED, and OFET.^8^

In this library, the starting *o*-bromo aromatic aldehyde was systematically varied, whereas the other key synthon for annulation, 3-thiopheneacetic acid, was kept constant. The structures of the newly synthesized compounds, together with the isolated yields, are reported in Table 1. Several aldehydes were used that incorporate electron-rich (thiophene, pyrrole, indole, furan residues) and electron-deficient (pyridine) residues. *o*-Bromo aromatic aldehydes **3a**–**e** and **3i** were commercially available, aldehyde **3f** was prepared though benzylic bromination and oxidation of the commercially available 2-bromo-3-methylbenzothiophene, and aldehydes **3g** and **3h** were prepared from commercially available 2-coumaranone and indolin-2-one through a Vilsmeier–Hack reaction. Conditions and full characterization details are reported in the Supporting Information.

**Table 1:**
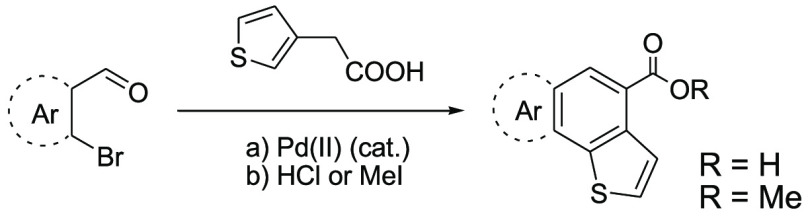

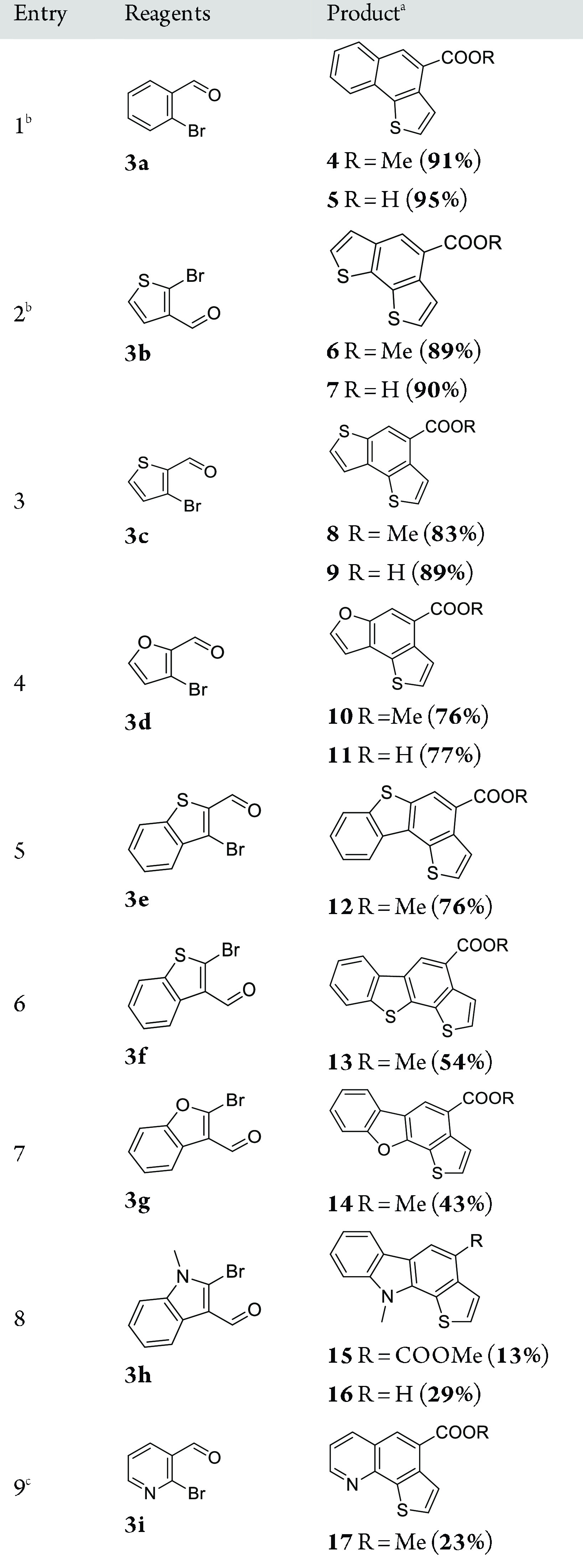
Reaction sScheme for DHA-Cross-Aldol Condensation between *o*-Bromoaldehydes Used as Starting Materials and 3-Thiopheneacetic Acid

a Yield (in parentheses) calculated after purification by acidification or flash chromatography. Reaction conditions: *o*-bromoaldehyde (1 equiv), 3-thiopheneacetic acid (1 equiv), Pd(OAc)_2_ (0,01 equiv), PPh_3_ (0,1 equiv), K_2_CO_3_ (3 equiv) in dry DMF (5 mL) at 110 °C for 12 h.

b Data taken from ref (6a).

c Reaction carried out for 1 week.

All of the reactions detailed in Table 1 were performed using the same reaction conditions. The reactions were quenched with concentrated HCl and filtration gave pure carboxylic acid products, whereas the methyl esters are easily obtained by addition of MeI at room temperature in a one-pot procedure. Only filtration through a short silica gel pad was required to isolate pure ester products. Excellent isolated yields were obtained for ester and free acids in almost all entries.

Slightly lower yields were obtained with benzothiophene and benzofuran derivatives (entries 5–7). In the case of the indole-containing aldehyde **3h**, the isolated yield of annulated products was comparable to the previous entries (42%), but decarboxylation occurred so that both products **15** and **16** were isolated and characterized. Both products, in the same ratio, were obtained when reaction times were prolonged to 4 days, other conditions being equal. In the case of entry 9, the synthesis of pyridine-thiophene mixed compound **17** using commercially available 2-bromonicotinaldehyde **3i** failed when the reaction was carried out for 12 h, but the desired product could be obtained in moderate yields (33%) when the reaction was carried out for substantially longer reaction times (7 days). This could be the result of the coordinating ability of the nitrogen containing heterocyclic system toward the catalyst.

Our protocols are noteworthy for their efficiency and regiodirected outcome. Specifically, it is possible to achieve the rapid and high yielding synthesis of benzodithiophene (BDT) scaffolds with both thiophenes on the same side (“linear”, compound **6**) or with an inverted thiophene structures (“bent”, not previously reported in the literature, compound **8**), merely by changing the position of the bromine and aldehyde functionalities on the starting substrates. The same complete regiospecificity could be observed by using benzothiophene derivatives **3e** and **3f**. The complete regiodirection of the reaction is guaranteed by the previously described mechanism,^9^ wherein the DHA step occurs first and directs the subsequent cross aldol reaction step to afford the desired product. In fact, cross-aldol byproducts occurring without annulation were not observed in the ^1^H NMR of any of the crude reaction mixtures reported in Table 1. The lack of polymeric byproducts that might be expected from Pd-calalyzed DHA homocoupling of the bromoaryls is further proof of the high selectivity and efficiency of the intermolecular DHA. The possibility to obtain both acid or ester derivatives in one-pot procedures affords a wide range of interesting postfunctionalization scenarios.

The substitution patterns of aromatic rings generates significant different conjugation pathways, and consequently diverse physical properties. In fact, “linear” and “bent” annulations produce conjugated structures, such as **6** and **8**, in which quinoidal resonance forms are much more or much less likely to describe the molecule, respectively. These resonance considerations are strong predictors of the electronic nature of semiconducting polymers. Inspection of the chemical shifts in the ^1^H NMR spectra (Figure 2) of **6** and **8** revealed little differences. The differences in the ^1^H NMR are more strongly dependent on the nature of five-membered rings (thiophene or furan, see **8** vs **10**) as demonstrated by the large shift of several proton signals. Unfortunately, a comparison between “linear” and “bent” annulation products was not feasible for the furan heterocycles because 2-bromofuran-3-carbaldehyde is an unknown compound and all synthetic procedures attempted failed to give this product. Compounds **6** and **8** show very similar π–π* transition energies, as indicated by the λ_max_ of their UV/vis spectra with nearly superimposable emission spectra. UV–vis and emission spectra for all compounds synthesized are shown in the SI.

**Figure 2:**
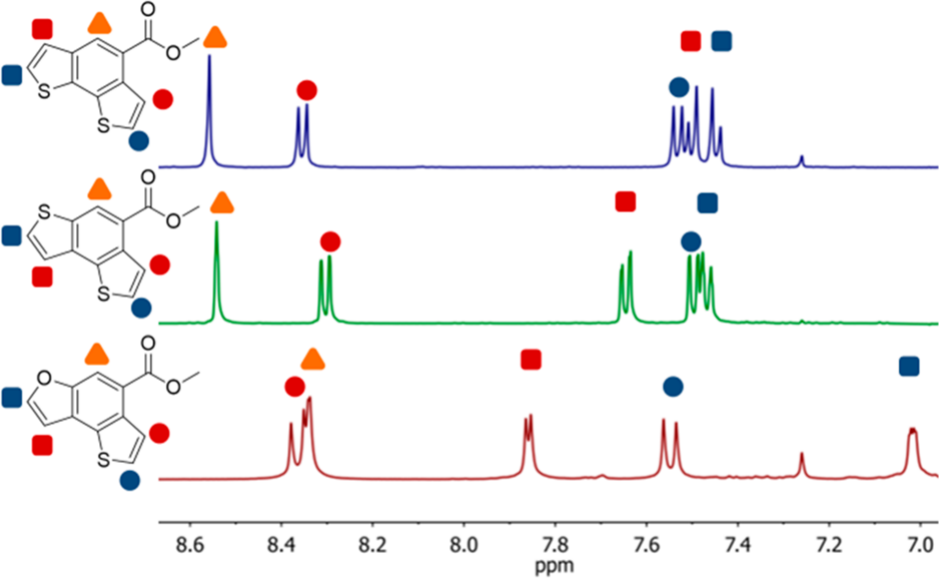
Stacked ^1^H NMR spectra (CDCl_3_, 400 MHz) of the aromatic region for compounds **6** (blue), **8** (green), and **10** (red).

We have investigated the extension of our synthetic protocols to generate larger π-systems. “Linear” and “bent” benzodithiophene derivatives **12** and **13**, respectively, as a result of their high planarity and π-conjugation, were identified as good candidates for the rapid construction of oligomers incorporating electron-withdrawing BT units as the central cores to create Donor–BT–Donor (D–BT–D) architectures (Scheme 1).

**Scheme 1:**
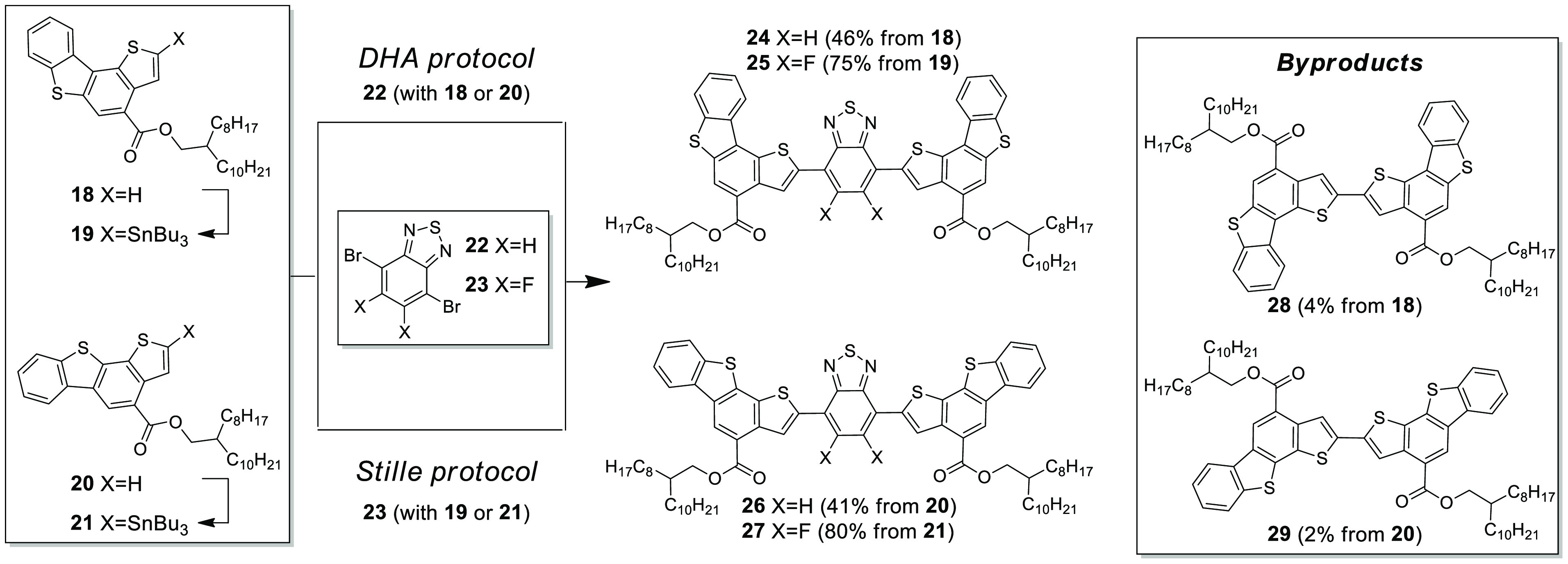
Synthesis of Donor–BT–Donor Oligomers **24**–**27**, and Structure of Side Products **28** and **29** DHA protocol: Pd(OAc)_2_, PPh_3_, PivOH, K_2_CO_3_, dry DMF, 120°C, 24 h. Stille protocol: Pd(PPh_3_)_4_, dry toluene, reflux, 24 h.

In order to improve solubility, long alkyl chains were installed on the π-scaffolds of **12** and **13**. Following the DHA-cross-aldol one-pot alkylation protocol, ester derivatives **18** and **20**, bearing C_20_ branched alkyl chains, were obtained in good yields (46% and 51%, respectively). Synthons **18** and **20** were initially screened for DHA reactivity using commercially available BT derivative **22**, obtaining **24** (46%) and **26** (41%) after purification by flash chromatography. Side products **28** and **29**, the result of homocoupling, often observed in DHA reactions,^5^ were isolated in minor yields (4% and 2% respectively), and no starting material was recovered. The extension of the DHA protocol for the synthesis of compounds **25** and **27** using fluorinated BT **23** with compounds **18** and **20** did not lead to the formation of the desired products. To overcome the synthetic difficulties observed using the DHA protocol, we evaluated the synthesis of compound **25** and **27** using a Stille protocol. Compounds **19** and **21** were obtained in good yields by stannylation of compounds **18** and **20** after lithiation by LDA and quenching with tributyltin chloride. Compounds **25** and **27** were thus obtained by Stille reactions in 75% and 80% yields, respectively, after flash chromatography.

The photophysical properties of dyes **24**–**27** in CHCl_3_ chloroform are summarized in Table 2. The UV–vis spectra of all compounds show a low energy band between 460 and 480 nm likely arising from π*–*π* transitions.

**Table 2:** Basic Properties in Solution (CHCl_3_) of Compounds **24**–**27**

compd	λ_abs_ (nm)	ε (cm^–1^ mol^–1^·L)	λ_em_ (nm)	QY (%)	τ (ns)
**24**	314, 478	1.64 × 10^4^	590	81	4.0
**26**	303, 480	3.58 × 10^4^	588	91	4.1
**25**	312, 467	1.65 × 10^4^	569	95	2.6
**27**	301, 466	3.79 × 10^4^	567	93	3.2

All oligomers exhibit intense broad emission bands centered between 570 and 590 nm typical of conjugated oligomers with substantial degrees of conformational freedom. All oligomers are highly fluorescent, with quantum yields between 0.81–0.95 and large Stokes shifts (over 100 nm). PL and UV–vis spectra of “linear” and “bent” benzodithiophene-containing oligomeric couples **24**–**26** and **25**–**27**, respectively, show that the two regioisomeric scaffolds have similar electron-donating ability toward the BT core. Comparison of the UV–vis spectra of **24** with **25** and **26** with **27** reveal significant red shifts of 10 nm upon fluorination. The bathochromic shifts by the introduction of fluorine in the BT backbone are in agreement with previous observations.^10^ HOMO–LUMO levels of the oligomers, as estimated by cyclic voltammetry (Figure 3), displayed a detectable lowering of the HOMO level in the “bent” vs “linear” derivative with the BT core (**24** vs **26**). To understand the role of the HOMO–LUMO structures in regard to the intended D–BT–D structures, density functional theory (DFT) calculations were undertaken for oligomers **24**–**27** (see Supporting Information). As expected, the HOMO is delocalized primarily over the Donor unit, while the LUMO resides primarily on the BT unit.

**Figure 3:**
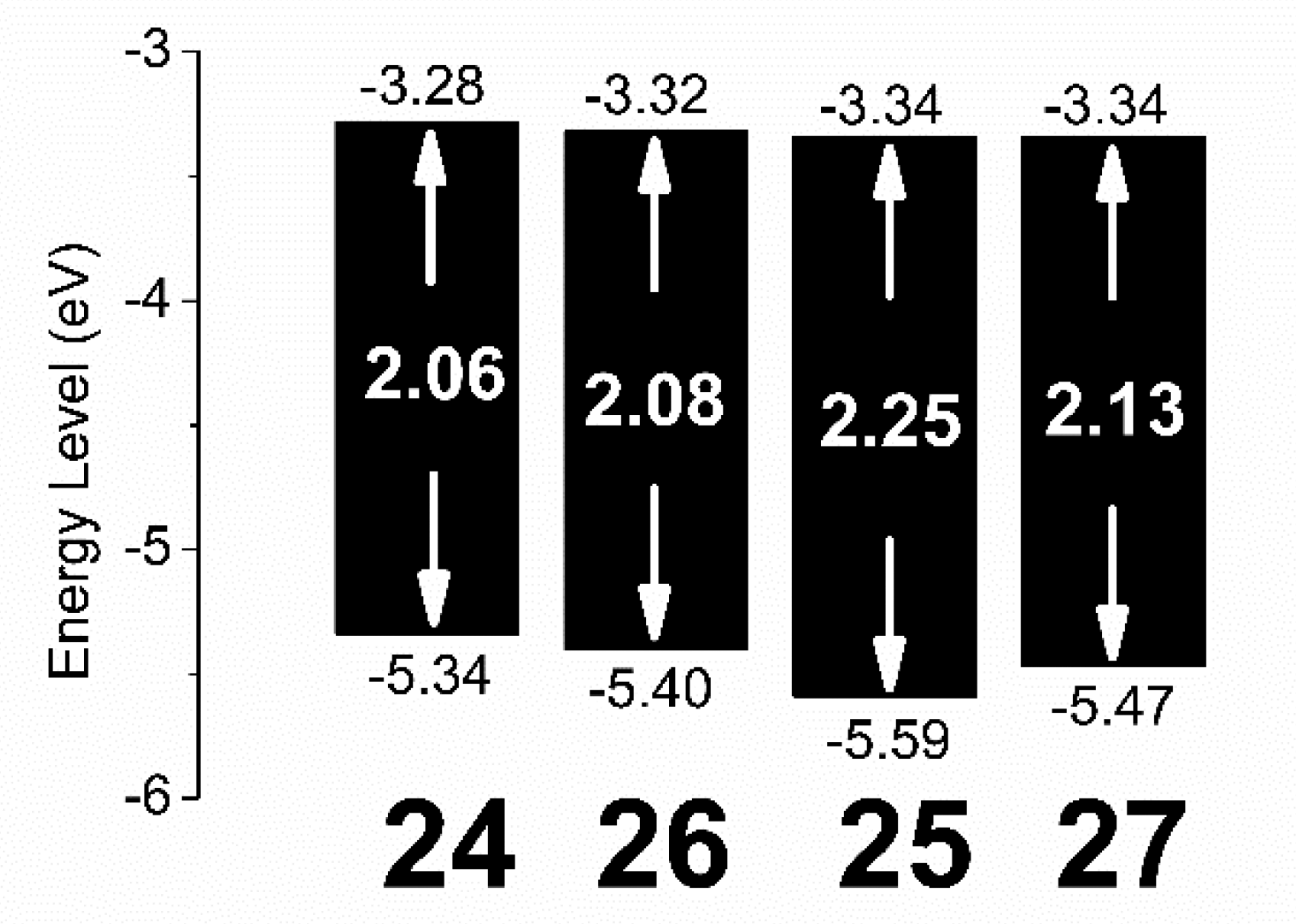
CV-derived energy level diagrams for **24**–**27**.

In conclusion, we have demonstrated that the DHA-cross aldol reaction is an efficient methodology for the annulation of several heteroaryl building blocks. Our one-pot method provides different typologies of polycyclic extended π-systems, with up to four fused rings containing alternating thiophene rings with several heterocyclic systems. Four novel D–BT–D highly emissive oligomers were synthesized, which as a consequence of their high quantum yields and large Stokes shifts are promising candidate for applications as luminescent solar concentrators. The regiodirected^9^ methodology can be susceptible of various interesting modifications, developing chromophores with substantially different shapes for applications as organic electronic materials. Given the importance of generating different molecular isomers for the tailoring of electronic and magnetic properties properties,^11^ and for the construction of chiral helical chromophores,^12^ the presented chemistry is a valuable new method for organic materials community.

## Supporting Information Available

Full experimental details and synthetic routes of elaborated precursors and new compounds; figures about UV–vis and photoluminescence spectroscopy; copies of ^1^H, ^13^C NMR, ESI-MS, and GC–MS spectra for new compounds (PDF)
FAIR data including the primary NMR FID files for compounds **9**, **10**, **17**, **18**, **19**, **20**, **21**, **24**, **25**, **26** (^1^H and ^13^C NMR) and **8**, **11**, **14**, **15**, **16**, **27** (ZIP)
