# Supplementary material for: One-Pot Regiodirected Annulations for the Rapid Synthesis of π-Extended Oligomers
Source: Org Lett. 2020 Apr 7;22(8):3263–7. doi: 10.1021/acs.orglett.0c01043 (PMC7997634; doi:10.1021/acs.orglett.0c01043)
Supplement: Supplementary file 1 — ol0c01043_si_001.pdf [file ol0c01043_si_001.pdf]

Supporting Information for:

One-Pot Regiodirected Annulations for the Rapid Synthesis of π-Extended Oligomers

Andrea Nitti, Peshawa Osw, Giuseppe Calcagno, Chiara Botta, Samuel I. Etkind, Gabriele Bianchi, Riccardo Po, Timothy M. Swager and Dario Pasini*

Table of Contents

1. General experimental *S2*

2. Synthesis of known compounds *S2*

2. Synthesis of new compounds *S4*

3. Additional experiments *S10*

4. References *S16*

5. Characterization of New Compounds *S17*

1. General Experimental

All commercially available reagents and solvents were purchased from Sigma-Aldrich, Fluorochem and Alfa Aesar. They were all used as received. Flash chromatography was carried out using Merck silica gel 60 (pore size 60 Å, 270-400 Mesh). ^1H and ^{13}C NMR spectra were recorded from solutions in deuterated solvents on 300 Bruker spectrometers or 400 Jeol with tetramethylsilane as internal standard. Low resolution mass spectra of pure compounds were recorded using Agilent Technologies ESI-MS Spectrometer instrument and a Thermofisher Finnigan TRACE DSQ GCMS instrument equipped with Direct Exposure Probe (DEP). Samples for the ESI-MS experiment were dissolved in a mixture of THF/MeOH 1:1, while samples for the DEP-MS were dissolved in a good solvent (eg. DCM) and settled on the probe filament. High resolution mass spectra were recorded using a Bruker Autoflex MALDI-TOF in reflectron mode with trans-2-[3-(4-tert-Butylphenyl)-2-methyl-2-propenylidene]malononitrile (DCTB) as a matrix. The UV-Vis spectroscopic studies were recorded using JASCO V-550 spectrophotometer. The PL and PLE spectra were recorded using Perkin Elmer LS55 luminescence spectrophotometer and Horiba Fluorolog. Cyclic voltammetry experiments were carried out using a Biologic SP-150 potentiostat with a polished glassy carbon working electrode, platinum counter electrode, silver pseudoreference electrode, and tetrabutylammonium hexofluorophosphate (recrystallized three times from EtOH) as a supporting electrolyte. Sample concentrations were between 0.2 and 1.0 mM. All electrochemical measurements were reference to the Fc/Fc^+ redox couple. Band gaps were estimated using the onset of the initial oxidation and reduction events, and E_{HOMO} and E_{LUMO} were estimated given an E_{HOMO} of 4.80 eV for ferrocene.[1] Theoretical calculations were carried out with the Spartan '18 (1.4.4, Wave function Inc, Irvine CA) software packages on a computer operating with Windows 10 OS. Geometry optimizations and energy calculations was performed with density functional theory (DFT) calculations using the ωB97X-D functional with the 6-31G* basis set in the gas phase. Solubilizing alkyl groups were replaced with methyl groups for simplicity.

2. Synthesis of known compounds

Scheme S1. Synthesis of aldehyde 3g.

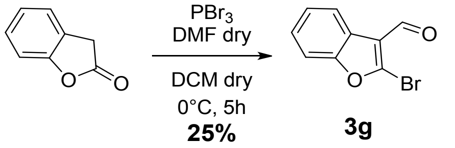

Compounds **3g** was synthesized in one step adapting the Vilsmeier-Hack protocol reported in literature for compound **3h**.[2]

2-bromobenzofuran-3-carbaldehyde (3g). A dried double neck flask purged with argon and sealed with a septum cap was charged with dry DCM (1 mL) and dry DMF (1 mL), cooled to 0 °C with an ice-water bath and PBr_3 (469 µL, 5 mmol, 2.5 eq) was added dropwise. The solution was stirred for 1 h at 0 °C, then benzofuran-2(*3H*)-one (268 mg, 2 mmol, 1.0 eq) was dissolved in dry DCM (3 mL) and added via syringe.

The reaction was refluxed for 12 h with an oil bath. After TLC monitoring, the solvent was removed under reduced pressure yielding an orange-brown lumpy solid. Purification by column chromatography (SiO_2, 8 : 2 diethyl ether : *n*-hexane) yielded pure **3g** as a pale yellow solid (220 mg, 46%). ^{1}H NMR (200 MHz, $CDCl_3$) δ = 10.05 (s, 1H), 8.13 (dd, J = 5.6, 3.1 Hz, 1H), 7.43-7.50 (m, 1H), 7.33-7.40 (m, 2H).

Scheme S2. Synthesis of aldehyde 3h

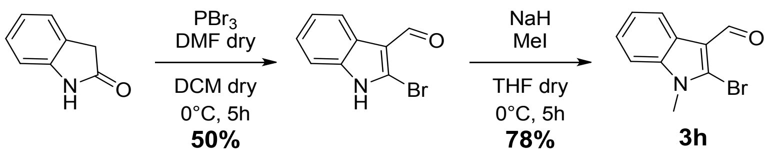

Compounds **3h** was synthetized adapting a Vilsmeier-Hack protocol reported in the literature.[2]

2-bromo-1*H*-indole-3-carbaldehyde. A dried double neck flask purged with argon and sealed with a septum cap was charged with dry DCM (6.0 mL) and dry DMF (1.8 mL), cooled to 0 °C with an ice-water bath and PBr_3 (5.8 g, 19 mmol, 2.5 eq) was added dropwise. The solution was stirred for 1 hour at 0 °C, then indolin-2-one (1.0 g, 7.5 mmol, 1.0 eq) in dry DCM (10 mL) was added via syringe. The reaction was refluxed for 5 h with an oil bath. During this time the reaction mixture showed an initial orange-white double layer changing into a limpid copper color. The color kept on changing into dark brown till a final dark blue. When a TLC check showed the reaction ending, the solvent was removed under reduced pressure yielded an orange-brown lumpy solid. Purification by column chromatography (SiO_2, 8:2 diethyl ether : *n*-hexane) yielded the pure product (507.8 mg, 50%). ^{1}H NMR (200 MHz, DMSO-d_6) δ = 13.03 (s, 1H), 9.89 (s, 1H), 8.07 (dd, J = 5.6, 3.1 Hz, 1H), 7.43 (dd, J = 5.9, 2.8 Hz, 1H), 7.35 – 7.11 (m, 2H). ^{13}C NMR (75 MHz, $CDCl_3$) δ = 184.5, 136.4, 124.8, 123.8, 123.3, 122.7, 119.7, 114.3, 111.7

2-bromo-1-methyl-1*H*-indole-3-carbaldehyde (3h). A dried double neck flask purged with argon was charged with 2-bromo-1*H*-indole-3-carbaldehyde (300 mg, 1.34 mmol, 1.0 eq), dry THF (14 mL), cooled to 0°C with an ice-water bath and NaH (96.4 mg, 4.0 mmol, 3.0 eq) was added in one portion. A color change from red to green was observed. The reaction mixture was kept under stirring for 30 min, then iodomethane (570 mg, 4.0 mmol, 3.0 eq) was added and the color changed to orange. The reaction was warmed to room temperature and kept for 2 h. The reaction was quenched with water and the solvents were removed under reduced pressure. The reaction mixture was purified by flash chromatography (SiO_2; *n*-hexane : AcOEt 8 : 2) affording a yellow solid (223 mg, 78%). ^{1}H NMR (200 MHz, $CDCl_3$) δ = 10.05 (s, 1H), 8.44–8.16 (m, 1H), 7.35 (s, 3H), 3.86 (s, 3H).

Scheme S3. Synthesis of aldehyde 3f.

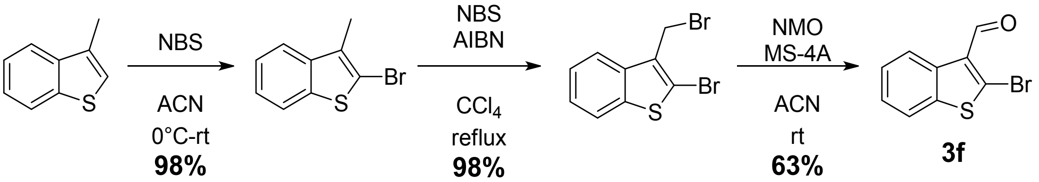

Compounds **3f** was synthetized adapting protocols reported in literature.[3,4,5]

2-bromo-3-methylbenzo[*b*]thiophene. A 10 mL round-bottom flask was equipped with a stir bar and cooled to 0 °C with an ice-water bath. Sequentially, 3-methylbenzothiophene (1,31 mL, 10 mmol, 1 eq), MeCN (12 mL, 0.85 mol·L^{-1}), and NBS (1.78 g, 10 mmol, 1 eq) were added. The ice-water bath was removed after 5 min and the solution was stirred at rt for 1h. The reaction was quenched with water (50 mL) and MeCN was removed. The residue was extracted with DCM (3×50 mL). The combined organic layers were dried over Na_2SO_4, filtered, and concentrated in vacuo. The resulting oil was purified via filtration on a short silica gel path using hexanes as the eluent, to give 2.22 g of a pale yellow oil (98%). ^{1}H NMR (200 MHz, $CDCl_3$) δ = 7.73 (dd, *J* = 6.1, 2.7 Hz, 1H), 7.65 (dd, *J* = 6.1, 2.7 Hz, 1H), 7.41–7.32 (m, 2H), 2.40 (s, 2H).

2-Bromo-3-(bromomethyl)methylbenzo[*b*]thiophene. To a solution of 2-bromo-3-methylbenzo[*b*] thiophene (2.27 g, 10 mmol, 1 eq) in dry CCl_4 (10 mL, 1 mol·L^{-1}) were added AIBN (49 mg, 0.03 mmol, 0.03 eq) and finely powdered NBS (1.78 g, 10 mmol, 1 eq). The reaction mixture was heated at reflux in an oil bath for 30 min and cooled to room temperature. The floating succinimide was filtered off and washed with carbon tetrachloride (15 mL). The combined filtrate was concentrated in vacuo to afford 2.99 g of 2-bromo-3-bromomethythiophene (98%) as a white solid. ^{1}H NMR (200 MHz, $CDCl_3$) δ = 7.80 (d, *J* = 7.2 Hz, 1H), 7.74 (d, *J* = 7.2 Hz, 1H), 7.41 (m, 2H), 4.71 (s, 2H).

2-bromobenzo[*b*]thiophene-3-carbaldehyde (3f). To a stirring mixture of *N*-methylmorpholine-*N*-oxide (1.76 g, 15 mmol, 3 eq) and 4Å molecular sieves (11 g) in MeCN (50 mL, 0.1 mol·L^{-1}) at 0°C in an ice-water bath under N_2 was added dropwise neat 2-bromo-3-(bromomethyl)thiophene (1.53 g, 5 mmol, 1 eq) over 5 min via syringe. After 4 h, the reaction mixture was filtered directly through a short pad of SiO_2, eluting with 600 mL of EtOAc. The organic solvent was removed under reduced pressure to give 0.763 g of aldehyde **3f** as a yellow oil (63%), which became an off-white solid after storage at 5°C. ^{1}H NMR (400 MHz, $CDCl_3$) δ = 9.95 (s, 1H), 7.36 (d, 1H, *J* = 6.0 Hz), 7.29 (d, 1H, *J* = 6.0 Hz).

3. Synthesis of new compounds

General procedure for the DHA-cross aldol condensation. *Selected example for compound* **8**. 3-Bromo-2-thiophenecarboxaldehyde **3c** (191 mg, 1 mmol, 1 eq) was added to a solution of 3-thiopheneacetic acid (142 mg, 1 mmol, 1 eq), $Pd(OAc)_2$ (11 mg, 0.05 mmol, 0.05 eq), PPh_3 (26 mg, 0.02 mmol, 0.1 eq), K_2CO_3 (414 mg, 3 mmol, 3 eq) in dry DMF (5 mL, 0.2 mol·L^{-1}) under inert atmosphere and then stirred at 110 °C with an oil bath for 12 h. The reaction suspension was cooled to rt and iodomethane (160 μL, 3 mmol, 3 eq) was added in one portion. The reaction mixture was kept under stirring for a further 4 h. After removal of solvent under reduced pressure, the crude of reaction was purified by short flash chromatography (SiO_2; *n*-hexanes : AcOEt 95:5).

*Methyl benzo[1,2-b:3,4-b']dithiophene-4-carboxylate (***8***).* White solid (206 mg, 83%). ^{1}H NMR (300 MHz, $CDCl_3$) δ = 8.54 (t, *J* = 0.9 Hz, 1H), 8.30 (dd, *J* = 5.5, 0.9 Hz, 1H), 7.65 (dd, *J* = 5.4, 1.0 Hz, 1H), 7.50 (dd, *J* = 5.5, 0.9 Hz, 1H), 7.47 (dd, *J* = 5.4, 1.0 Hz, 1H), 3.99 (s, 3H).^{13}C NMR (75 MHz, $CDCl_3$) δ = 167.2, 137.9,

136.0, 125.9, 125.6, 125.5, 125.1, 125.0, 121.7, 52.0. DEP-MS: *m/z* 248. Anal. Calcd. For $C_{12}H_8O_2S_2$: C, 58.0; H, 3.2. Found: C, 58.0; H, 3.2.

*benzo[1,2-b:6,5-b']dithiophene-4-carboxylic acid (***9***)*. Grey solid (206 mg, 89%). ^{1}H NMR (300 MHz, DMSO-*d*$_6$) δ= 13.16 (s, 1H), 8.71 (s, 1H), 8.29 (d, *J* = 5.5 Hz, 1H), 8.18 (d, *J* = 5.5 Hz, 1H), 7.91 (d, *J* = 5.5 Hz, 1H), 7.79 (d, *J* = 5.4 Hz, 1H). ^{13}C NMR (75 MHz, DMSO-*d*$_6$) δ= 167.53, 136.38, 135.42, 134.88, 134.75, 133.22, 126.93, 125.16, 123.33, 121.81, 121.65. ESI-MS: *m/z* 233 [M-1]$^-$, 467 [2M-1]$^-$. Anal. Calcd. for $C_{11}H_6O_2S_2$: C, 56.6; H, 2.1. Found: C, 56.6; H, 2.2.

*methyl thieno[2,3-e]benzofuran-4-carboxylate (***10***)*. Beige solid (177 mg, 76%).^{1}H NMR (300 MHz, CDCl$_3$) δ= 13.15 (s, 1H), 8.71 (s, 1H), 8.30 (d, J = 5.5 Hz, 1H), 8.19 (d, J = 5.4 Hz, 1H), 7.91 (d, J = 5.5 Hz, 1H), 7.80 (d, J = 5.4 Hz, 1H). ^{13}C NMR (75 MHz, CDCl$_3$) δ= 167.5, 136.4, 135.4, 134.9, 134.8, 133.2, 126.9, 125.17, 123.3, 121.8, 121.7. DEP-MS: *m/z* 232. Anal. Calcd. for $C_{12}H_8O_3S$: C, 62.06; H, 3.47. Found: C, 62.0; H, 3.4.

*thieno[2,3-e]benzofuran-4-carboxylic acid (***11***)*. Brown solid (168 mg, 77%). ^{1}H NMR (300 MHz, DMSO-*d*$_6$) δ= 13.15 (s, 1H), 8.52 – 8.14 (m, 1H), 7.89 (d, *J* = 5.6 Hz, 1H), 7.37 (s, 1H). ^{13}C NMR (75 MHz, DMSO-*d*$_6$) δ= 167.5, 136.4, 135.4, 134.9, 134.8, 133.2, 126.9, 125.17, 123.3, 121.8, 121.7. ESI-MS: *m/z*: 217 [M-1]$^-$.. Anal. Calcd. for $C_{11}H_6O_3S$: C, 56.6; H, 2.2. Found: C, 56.6; H, 2.2.

*methyl 1H-benzo[b]thiophene[4,5-d]thiophene-4-carboxylate (***12***)*.Pale yellow solid (226 mg, 76%). ^{1}H NMR (300 MHz, CDCl$_3$) δ= 8.64 (s, 1H), 8.55 – 8.37 (m, 2H), 7.98 (dd, *J* = 6.6, 1.7 Hz, 1H), 7.76 – 7.44 (m, 3H), 4.05 (s, 3H). ^{13}C NMR (75 MHz, CDCl$_3$) δ= 166.7, 141.2, 136.5, 135.2, 135.0, 134.0, 132.5, 127.1, 125.8, 124.9, 124.7, 124.4, 122.9, 122.8, 122.7, 52.2. DEP-MS: *m/z* 298. Anal. Calcd. for $C_{16}H_{10}O_2S_2$: C, 64.4; H, 3.4. Found: C, 64.4; H, 3.3.

methyl 1H-benzo[b]thiophene[5,4-d]thiophene-4-carboxylate (**13**). Pale yellow solid (161 mg, 54%). ^{1}H NMR (300 MHz, CDCl$_3$) δ= 8.88 (s, 1H), 8.37 (d, *J* = 5.5 Hz, 1H), 8.30 – 8.19 (m, 1H), 7.95 – 7.86 (m, 1H), 7.62 (d, *J* = 5.5 Hz, 1H), 7.59–7.43 (m, 2H), 4.06 (s, 3H). ^{13}C NMR (75 MHz, CDCl$_3$) δ= 166.7, 141.2, 136.5, 135.2, 135.0, 134.0, 132.5, 127.1, 125.8, 124.9, 124.7, 124.4, 122.9, 122.8, 122.7, 52.2. DEP-MS: *m/z* 298. Anal. Calcd. for $C_{16}H_{10}O_2S_2$: C, 64.40 H, 3.4. Found: C, 64.4; H, 3.3.

Methyl benzo[b]thieno[3,2-g]benzofuran-4-carboxylate (**14**). Pale yellow solid (121 mg, 43% yield). ^{1}H NMR (200 MHz, CDCl$_3$) δ= 8.88 (s, 1H), 8.37 (d, *J* = 5.5 Hz, 1H), 8.30 – 8.19 (m, 1H), 7.95 – 7.86 (m, 1H), 7.62 (d, *J* = 5.5 Hz, 1H), 7.59 – 7.43 (m, 2H), 4.06 (s, 3H). ^{13}C NMR (75 MHz, CDCl$_3$) δ= 166.7, 141.2, 136.5, 135.2, 135.0, 134.0, 132.5, 127.1, 125.8, 124.9, 124.7, 124.4, 122.9, 122.8, 122.7, 52.2. ESI-MS: *m/z* 283 [M+1]$^+$. Anal. Calcd. for $C_{16}H_{10}O_3S$: C, 68.1; H, 3.6. Found: C, 68.0; H, 3.5.

*methyl 10-methyl-10H-thieno[2,3-a]carbazole-4-carboxylate (***15***)*. Yellow solid (38 mg, 13%) ^{1}H NMR (300 MHz, CDCl$_3$) δ= 8.12 (dd, *J* = 13.8, 8.0 Hz, 2H), 7.68 (d, *J* = 8.3 Hz, 1H), 7.55 (d, *J* = 5.3 Hz, 1H), 7.51 – 7.43 (m, 3H), 7.31 (s, 1H), 4.22 (s, 3H). ^{13}C NMR (75 MHz, CDCl$_3$) δ= 167.6, 140.8, 138.6, 138.4, 125.8, 125.5, 125.4, 123.3, 122.8, 122.2, 120.2, 120.0, 117.5, 115.9, 108.9. ESI-MS: *m/z* 296 [M+1]$^+$. Anal. Calcd. for $C_{17}H_{13}NO_2S$: C, 69.1; H, 4.4. Found: C, 69.1; H, 4.4.

*10-methyl-10H-thieno[2,3-a]carbazole (***16***)*. Yellow solid (69 mg, 29%). ^{1}H NMR (300 MHz, $CDCl_3$) δ = 8.79 (s, 1H), 8.53 (d, J = 5.5 Hz, 1H), 8.12 (d, J =7.7 Hz, 1H), 7.59 (d, J = 5.5 Hz, 1H), 7.53 – 7.20 (m, 3H), 4.12 (s, 3H), 4.04 (s, 3H). ^{13}C NMR (75 MHz, $CDCl_3$) δ = 167.6, 140.8, 138.6, 138.4, 125.8, 125.5, 125.4, 123.3, 122.8, 122.2, 120.2, 120.0, 117.5, 115.9, 108.9, 51.7, 31.1. DEP-MS: *m/z* 237. Anal. Calcd. for $C_{15}H_{11}NS$: C, 75.9; H, 4.7. Found: C, 75.9; H, 4.6.

*Methyl thieno[3,2-h]quinoline-4-carboxylate (***17***)*.Pale yellow solid (56 mg, 23% yield). ^{1}H NMR (300 MHz, $CDCl_3$) δ = 8.99 (dd, J = 4.3, 1.7 Hz, 1H), 8.52 (s, 1H), 8.35 – 8.28 (m, 2H), 7.76 (d, J = 5.5 Hz, 1H), 7.50 (dd, J = 8.2, 4.4 Hz, 1H), 4.04 (s, 3H). ^{13}C NMR (75 MHz, $CDCl_3$) δ = 166.5, 151.7, 146.0, 140.2, 137.8, 137.8, 129.6, 129.1, 125.7, 124.1, 124.0, 121.5, 52.3. ESI-MS: *m/z* 244 $[M+1]^+$. Anal. Calcd. for $C_{13}H_9NO_2S$: C, 64.2; H, 3.7. Found: C, 64.1; H, 3.7.

General procedure for the DHA-cross aldol condensation and alkylation with 9-(bromomethyl)nonadecane. *Selected example for compound* **18**. 3-bromobenzo[*b*]thiophene-2-carbaldehyde **3e** (2.41 g, 10 mmol, 1 eq) was added to a solution of 3-thiopheneacetic acid (1.42 g, 10 mmol, 1 eq), $Pd(OAc)_2$ (224 mg, 1 mmol, 0.1 eq), PPh_3 (524 mg, 2 mmol, 0.2 eq), K_2CO_3 (2.76 g, 20 mmol, 2 eq) in dry DMF (20 mL, 0.2 mol·L^{-1}) under inert atmosphere and then stirred at 110 °C in an oil bath for 24 h. The reaction suspension was cooled to 60°C and 9-(bromomethyl)nonadecane (3.62 g, 10 mmol, 1 eq) was added in one portion. The reaction mixture was kept under stirring for a further 12 h. After removal of solvent under reduced pressure, the crude of reaction was purified by flash chromatography (SiO_2; d=5 cm, h=35 cm; eluent: *n*-hexanes : AcOEt 98 : 2). Pure product was obtained as a pale yellow oil (2.88 g, 51%). TLC 7:3 *n*-hexanes : AcOEt, R_f = 0.49.

*2-octyldodecyl benzo[b]thieno[2,3-e]benzothiophene-4-carboxylate (***18***)*. Pale Yellow oil (2.88 g, 51%). ^{1}H NMR (200 MHz, $CDCl_3$) δ = 8.62 (s, 1H), 8.56 – 8.42 (m, 2H), 7.98 (dd, J = 6.9, 2.0 Hz, 1H), 7.73 – 7.45 (m, 3H), 4.37 (d, J = 5.7 Hz, 2H), 1.96 – 1.75 (m, 1H), 1.54 – 1.13 (m, 32H), 0.96 – 0.77 (m, 6H). ^{13}C NMR (75 MHz, $CDCl_3$) δ = 168.5, 138.2, 137.4, 137.1, 135.4, 134.3, 131.3, 126.5, 125.5, 124.7, 122.6, 121.9, 121.5, 121.3, 77.2, 76.7, 76.3, 67.8, 37.3, 31.6, 31.4, 29.8, 29.4, 29.4, 29.3, 29.1, 26.6, 22.4, 13.8. DEP-MS: *m/z* 565. Anal. Calcd. for $C_{35}H_{48}O_2S_2$: C, 74.4; H, 8.6. Found: C, 74.2; H, 8.9.

*2-octyldodecyl benzo[b]thieno[3,2-g]benzothiophene-4-carboxylate (***20***)*. Pale Yellow oil (2.60 g, 46%). ^{1}H NMR (300 MHz, $CDCl_3$) δ = 8.78 (s, 1H), 8.35 (d, J = 5.5 Hz, 1H), 8.19 – 8.08 (m, 1H), 7.90–7.75 (m, 1H), 7.57 (d, J = 5.4 Hz, 1H), 7.46 (hept, J = 5.4 Hz, 2H), 4.39 (d, J = 5.7 Hz, 2H), 1.93 (t, J = 5.8 Hz, 1H), 1.59 – 1.18 (m, 32H), 0.89 (t, J = 6.5 Hz, 6H). ^{13}C NMR (75 MHz, $CDCl_3$) δ = 166.5, 138.2, 137.6, 137.2, 135.4, 134.4, 131.3, 126.5, 125.5, 124.7, 122.6, 121.9, 121.5, 121.3, 77.2, 76.7, 76.3, 67.8, 37.3, 31.6, 31.4, 29.8, 29.4, 29.4, 29.3, 29.1, 26.6, 22.4, 13.8. DEP-MS: *m/z* 565. Anal. Calcd. for $C_{35}H_{48}O_2S_2$: C, 74.4; H, 8.6. Found: C, 74.2; H, 8.8.

General procedure for stannylation. *Selected example: compound* **19**. A solution of compound **18** (1 g, 1.77 mmol, 1eq) in dry THF (35 mL) under argon atmosphere was cooled to -78 °C in a dry ice-acetone bath. After 15 min a 1 M solution of LDA in Hexane (2.66 mL; 2.66 mmol, 1.5 eq) was added dropwise and kept for 2h at same temperature, than Bu_4SnCl (960 µL, 3.54 mmol, 2 eq) was added in one portion. The reaction mixture after 30 min at -78°C was warmed to room temperature and kept at same temperature overnight. The reaction mixture was quenched with water, the solvent removed under reduced pressure, extracted with Et_2O (3 times) and collected organic phases was dried over Na_2SO_4. After removal of the solvent under reduced pressure, crude of reaction was purified by flash chromathography on silica gel (eluent: *n*-hexanes) previously conditioned with a mixture of 9 : 1 *n*-hexanes: triethylamine (TEA) and washed with n-Hexane affording pure compound as colorless oil (1.10 g, 73%).

*2-octyldodecyl 2-(tributylstannyl)benzo[b]thieno[2,3-e]benzothiophene-4-carboxylate (***19***).* Pale yellow oil (1.10 g, 73%). ^{1}H NMR (400 MHz, $CDCl_3$) δ = 8.68 – 8.44 (m, 3H), 7.99 (d, J = 7.5 Hz, 1H), 7.72 – 7.52 (m, 2H), 4.38 (d, J = 5.4 Hz, 2H), 1.98 – 1.77 (m, 1H), 1.76 – 1.14 (m, 50H), 1.01 – 0.78 (m, 15H). ^{13}C NMR (75 MHz, $CDCl_3$) δ = 168.1, 141.5, 140.1, 139.9, 139.0, 138.3, 136.9, 133.9, 130.5, 126.3, 125.2, 123.2, 122.1, 121.8, 121.6, 37.3, 32.1, 31.8, 30.3, 29.6, 29.5, 29.3, 29.2, 29.1, 28.8, 27.4, 27.1, 22.5, 14.1, 13.6, 11.1.

*2-octyldodecyl 2-(tributylstannyl)benzo[b]thieno[3,2-g]benzothiophene-4-carboxylate (***21***).* Pale yellow oil (1.19 g, 79%). ^{1}H NMR (300 MHz, $CDCl_3$) δ = 8.86 (s, 1H), 8.43 (s, 1H), 8.24 (dd, J = 7.6, 1.6 Hz, 1H), 7.90 (d, J = 1.8 Hz, 1H), 7.57 – 7.39 (m, 2H), 4.40 (d, J = 5.7 Hz, 3H), 2.02 – 1.84 (m, 1H), 1.80 – 1.07 (m, 38H), 1.07 – 0.70 (m, 15H). ^{13}C NMR (75 MHz, $CDCl_3$) δ = 167.5, 141.9, 139.7, 139.1, 138.9, 137.7, 136.2, 133.7, 130.9, 126.8, 125.2, 123.2, 122.1, 121.7, 121.6, 37.7, 32.1, 31.98, 30.2, 29.8, 29.8, 29.5, 29.3, 29.1, 29.0, 27.4, 27.0, 22.8, 14.2, 13.8, 11.2.

General procedure for the direct arylation protocol for the synthesis of oligomers. *Selected example for the* **s***ynthesis of compound* **24.** 4,7-dibromobenzo[*c*][1,2,5]thiadiazole **22** (150 mg, 0.51 mmol) was added to a solution of compound **18** (519 g, 1,02 mmol), $Pd(OAc)_2$ (11 mg, 0.05 mmol), PPh_3 (26 mg, 0.1 mmol), K_2CO_3 (138 mg, 1 mmol) in dry DMAc (3 mL) and then warmed to 110 °C with an oil bath for 24 h. After TLC monitoring (eluent 7:3 petroleum ether : DCM, R_f = 0.39), reaction solvent was removed under reduce pressure. Crude of reaction was purified by flash chromatography (SiO_2; eluent: petroleum ether : DCM 8 : 2) to give pure product as red solid (296 mg, 46%).

diicosyl 2,2'-(benzo[c][1,2,5]thiadiazole-4,7-diyl)bis(benzo[b]thieno[2,3-e]benzothiophene-4-carboxylate) (**24**). Red solid (296 mg, 46%). ^{1}H NMR (400 MHz, $CDCl_3$) δ = 9.22 (s, 2H), 8.27 (d, J = 7.2 Hz, 2H), 8.16 (d, J = 4.9 Hz, 2H), 7.81 – 7.73 (m, 2H), 7.71 (s, 2H), 7.52 – 7.37 (m, 4H), 4.33 – 4.25 (m, 4H), 1.98 – 1.86 (m, 2H), 1.63 – 1.14 (m, 64H), 0.93 – 0.78 (m, 12H). ^{13}C NMR (101 MHz, $CDCl_3$) δ = 165.9, 152.0, 140.9, 138.4, 136.7, 135.3, 133.6, 133.5, 131.4, 129.5, 127.0, 126.6, 125.7, 124.7, 124.5, 124.4, 122.6, 122.4, 68.1, 37.3, 31.8, 31.4, 30.0, 29.6, 29.2, 26.7, 22.5, 13.9. HRMS (MALDI-TOF): Calcd. for $C_{76}H_{96}N_2O_4S_5$: 1260.5974. Found: 1260.6020.

bis(2-octyldodecyl) [2,2'-bibenzo[b]thieno[2,3-e]benzothiophene]-4,4'-dicarboxylate (**28**). Pale yellow oil (23 mg, 4%). ^{1}H NMR (200 MHz, $CDCl_3$) δ = 8.65 (s, 2H), 8.35 (d, J = 5.6 Hz, 4H), 7.85 (d, J = 7.5 Hz, 2H), 7.61 – 7.42 (m, 4H), 4.35 (d, J = 5.5 Hz, 4H), 1.91 (d, J = 5.4 Hz, 2H), 1.26 (s, 64H), 0.87 (dd, J = 10.3, 4.3 Hz, 12H).

diicosyl 2,2'-(benzo[c][1,2,5]thiadiazole-4,7-diyl)bis(benzo[b]thieno[3,2-g]benzothiophene-4-carboxylate) (**26**). Red solid (264 mg, 41%). ^{1}H NMR (400 MHz, $CDCl_3$) δ = 8.89 (s, 2H), 8.27 (s, 2H), 7.65 (d, J = 7.8 Hz, 4H), 7.48 (s, 2H), 7.28 – 7.19 (t, J = 7.4 Hz, 2H), 7.13 (t, J = 7.4 Hz, 2H), 4.45 (d, J = 5.8 Hz, 4H), 2.08 – 1.96 (m, 2H), 1.69 – 1.14 (m, 64H), 0.93 – 0.79 (m, 12H). ^{13}C NMR (101 MHz, $CDCl_3$) δ = 166.9, 152.3, 139.7, 139.1, 137.7, 137.2, 135.9, 135.3, 132.0, 126.9, 126.1, 125.8, 125.8, 125.1, 123.0, 122.0, 121.9, 121.8, 68.6, 38.2, 32.5, 32.4, 32.2, 30.7, 30.3, 29.9, 27.5, 23.2, 14.6. HRMS (MALDI-TOF): calcd. $C_{76}H_{96}N_2O_4S_5$: 1260.5974. Found: 1260.5976.

bis(2-octyldodecyl) [2,2'-bibenzo[b]thieno[3,2-g]benzothiophene]-4,4'-dicarboxylate (**29**). Pale yellow oil (11 mg, 2%). ^{1}H NMR (300 MHz, $CDCl_3$) δ = 8.64 (s, 2H), 8.46 (s, 2H), 8.00 (d, J = 3.8 Hz, 2H), 7.77 (d, J = 5.0 Hz, 2H), 7.44 – 7.32 (m, 4H), 4.45 (d, J = 5.6 Hz, 4H), 2.05 – 1.89 (m, 2H), 1.64 – 1.16 (m, 58H), 0.83 (t, J = 6.3 Hz, 12H). ^{13}C NMR (75 MHz, $CDCl_3$) δ = 166.72, 138.83, 137.86, 137.26, 135.70, 134.43, 132.30, 127.10, 127.04, 125.16, 123.97, 122.95, 122.11, 121.93, 68.43, 37.86, 32.10, 32.06, 31.92, 30.30, 29.92, 29.87, 29.56, 29.52, 27.18, 22.83, 14.25.

General procedure for the Stille protocol for the synthesis of oligomers. *Synthesis of compound* **25.** A solution of icosyl 2-(tributylstannyl)benzo[*b*]thieno[2,3-*e*]benzothiophene-4-carboxylate **19** (118 mg, 0.21 mmol) in 1 mL of degassed dry toluene was added to a degassed solution of 4,7-dibromo-5,6-difluorobenzo[*c*][1,2,5]thiadiazole **23** (33 mg, 0.1 mmol), $Pd(PPh_3)_4$ (4.5 mg, 0.04 mmol, 0.05 eq), in dry toluene (1 mL) and then stirred to 110 °C in an oil bath for 24 h. After TLC monitoring (7 : 3 petroleum ether : DCM, R_f = 0.41), reaction solvent was removed under reduced pressure. The crude reaction mixture was purified by flash chromatography (SiO_2; eluent: petroleum ether : DCM 8:2). The pure product was obtained as orange solid (97 mg, 75%).

diicosyl 2,2'-(5,6-difluorobenzo[c][1,2,5]thiadiazole-4,7-diyl)bis(benzo[b]thieno[2,3-e]benzothiophene-4-carboxylate) (**25**). Red solid (97 mg, 75%). ^{1}H NMR (400 MHz, $CDCl_3$) δ = 9.12 (s, 2H), 7.98 (d, J = 7.4 Hz, 2H), 7.91 (d, J = 5.6 Hz, 2H), 7.57 (d, J = 7.4 Hz, 2H), 7.38 – 7.31 (m, 2H), 7.26 (t, J = 7.3 Hz, 2H), 4.26 (d, J = 5.2 Hz, 4H), 1.97 – 1.84 (m, 2H), 1.61 – 1.17 (m, 64H), 0.91 – 0.81 (m, 12H). ^{13}C NMR (101 MHz, $CDCl_3$) δ = 166.1, 148.3, 141.2, 136.4, 136.1, 135.8, 133.9, 131.5, 131.4, 128.8, 128.7, 126.9, 125.0, 124.9, 123.1, 123.0, 122.6, 111.7, 111.6, 68.8, 37.9, 32.5, 32.4, 31.9, 30.7, 30.3, 30.2, 29.9, 27.4, 23.2, 14.6. HRMS (MALDI-TOF): calcd for $C_{76}H_{94}F_2N_2O_4S_5$: 1296.5785. Found: 1296.5808.

diicosyl 2,2'-(5,6-difluorobenzo[c][1,2,5]thiadiazole-4,7-diyl)bis(benzo[b]thieno[3,2-g]benzothiophene-4-carboxylate) (**27**). Red solid (104 mg, 80%). ^{1}H NMR (400 MHz, $CDCl_3$) δ = 8.86 (s, 2H), 8.10 (s, 2H), 7.54 (d, J = 7.7 Hz, 2H), 7.41 (d, J = 7.7 Hz, 2H), 7.10 (t, J = 7.3 Hz, 2H), 6.91 (t, J = 7.2 Hz, 2H), 4.46 (d, J = 5.3 Hz, 4H), 2.10 – 1.90 (m, 2H), 1.72 – 1.16 (m, 64H), 0.88 (d, J = 7.4 Hz, 12H). ^{13}C NMR (101 MHz, $CDCl_3$) δ

= 166.6, 139.0, 136.5, 136.3, 135.8, 135.5, 132.2, 132.1, 129.3, 129.2, 126.8, 124.8, 122.8, 122.1, 122.0, 121.6, 111.3, 68.7, 38.2, 32.5, 32.4, 32.1, 30.8, 30.3, 29.9, 27.5, 23.2, 14.6. HRMS (MALDI-TOF): calcd. for $C_{76}H_{94}F_2N_2O_4S_5$: 1296.5785. Found: 1296.5833.

3. Additional experiments

Compound **24**

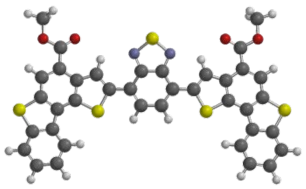

Compound **25**

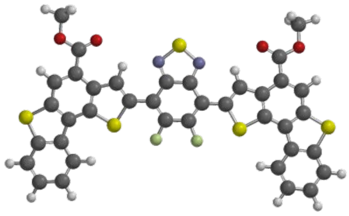

Compound **26**

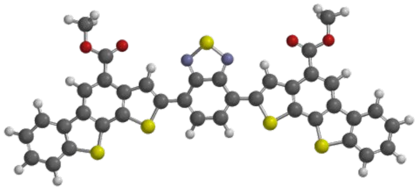

Compound **27**

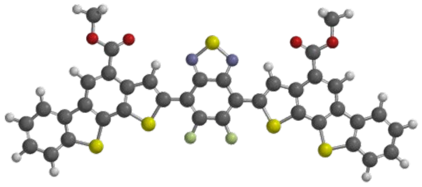

	HOMO	LUMO
**24**	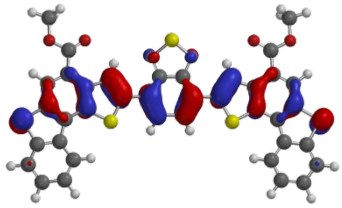	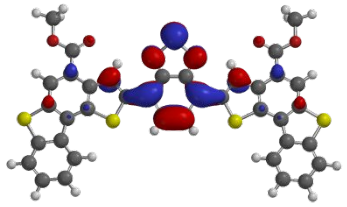
**25**	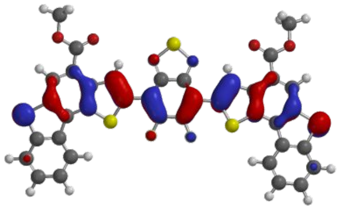	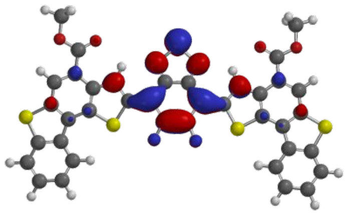
**26**	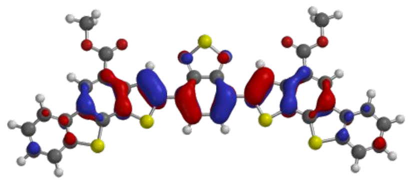	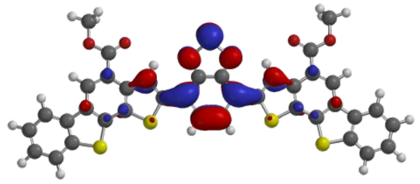
**27**	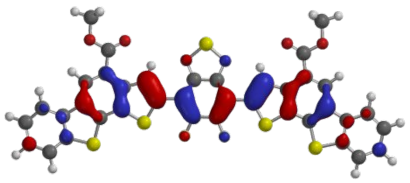	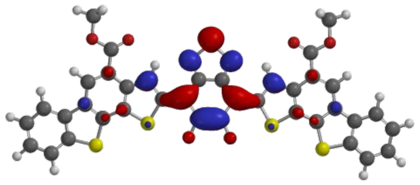

Figure S1. Computationally determined structures of the HOMO and LUMO for compounds **24-27**.

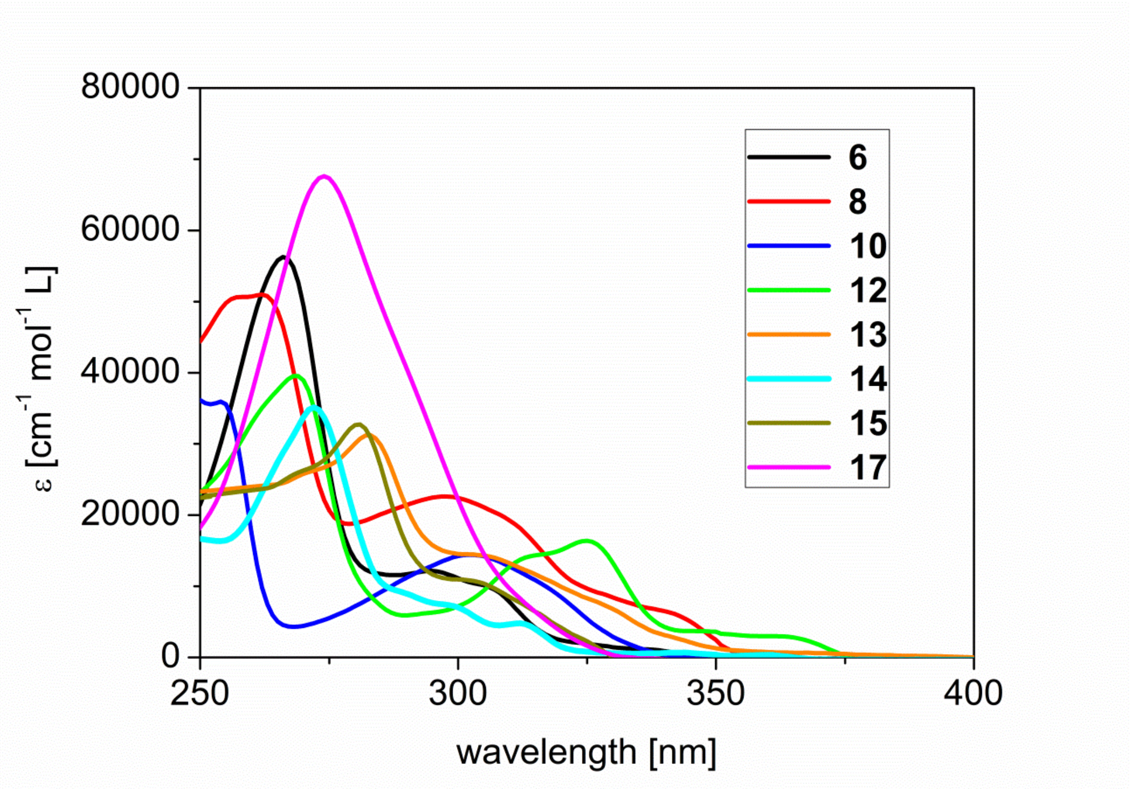

Figure S2. UV-Vis of compounds **6–17** in solution 10^{-5} mol·L^{-1} of $CHCl_3$.

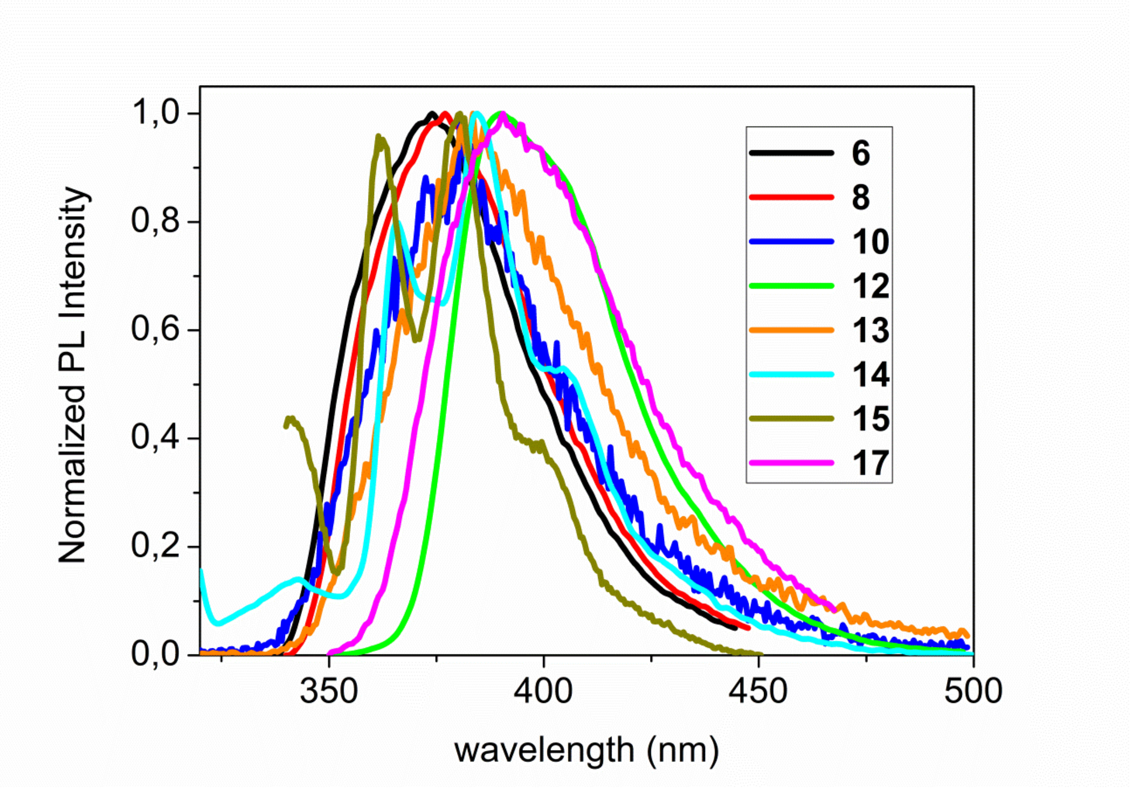

Figure S3. Normalized PL spectra of compound **6–17** in solution 10^{-5} mol·L^{-1} of $CHCl_3$.

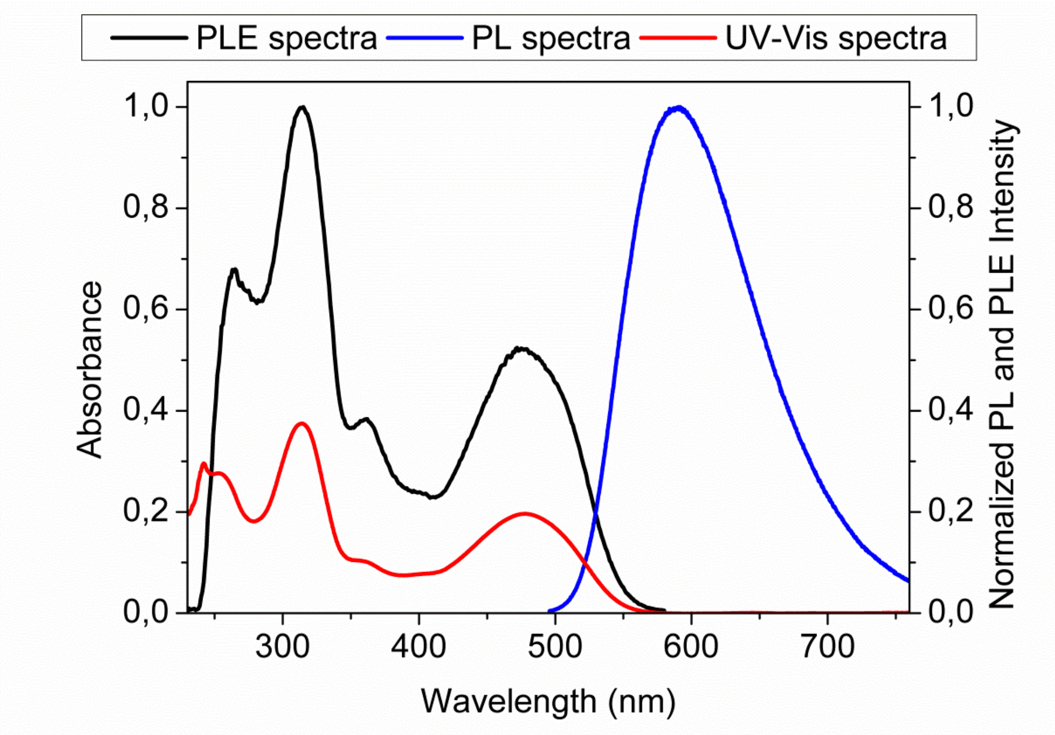

Figure S4. Spectroscopic characterization of compound **24** in $CHCl_3$ solution (10^{-5} mol L^{-1}). Red line: UV-Vis, Bue line: Normalized PL spectra, Black line: Normalized PLE spectra.

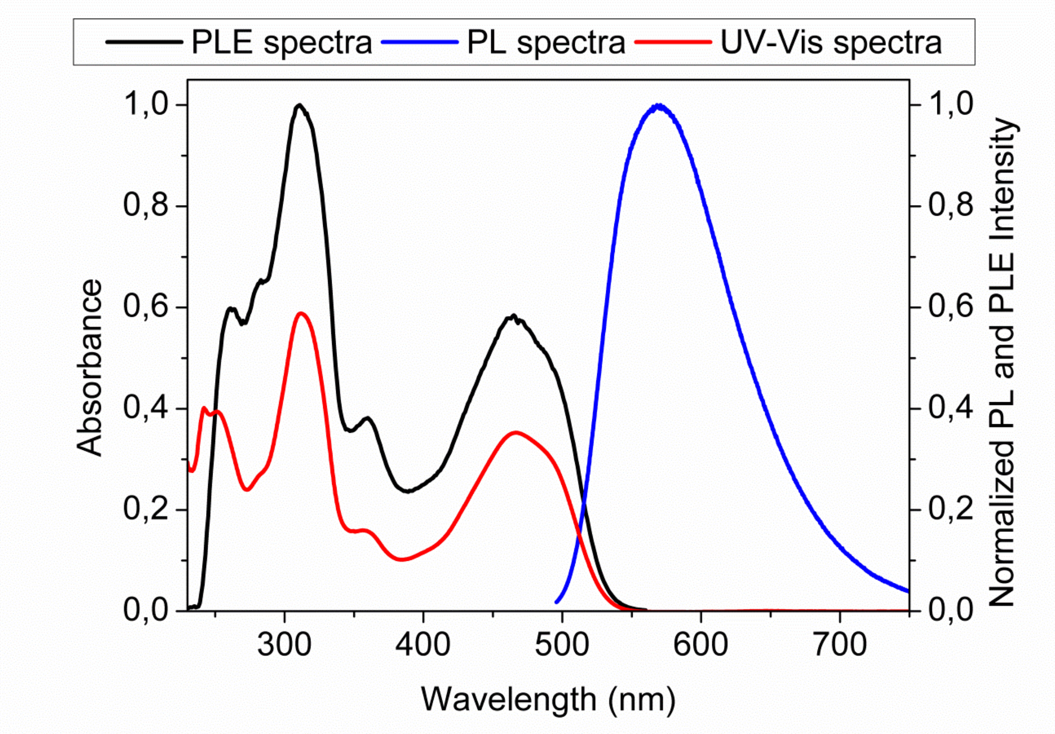

Figure S5. Spectroscopic characterization of compound **25** in $CHCl_3$ solution (10^{-5} mol L^{-1}). Red line: UV-Vis, Bue line: Normalized PL spectra, Black line: Normalized PLE spectra.

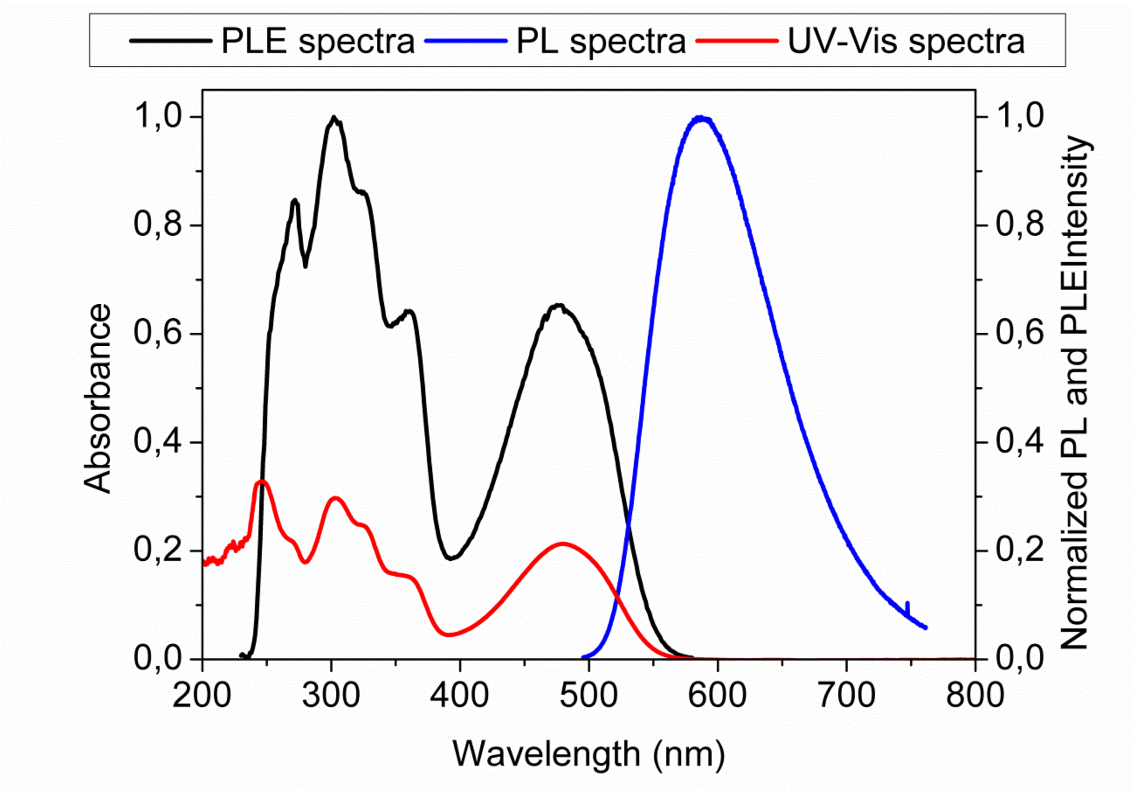

Figure S6. Spectroscopic characterization of compound **26** in $CHCl_3$ solution (10^{-5} mol L^{-1}). Red line: UV-Vis, Bue line: Normalized PL spectra, Black line: Normalized PLE spectra.

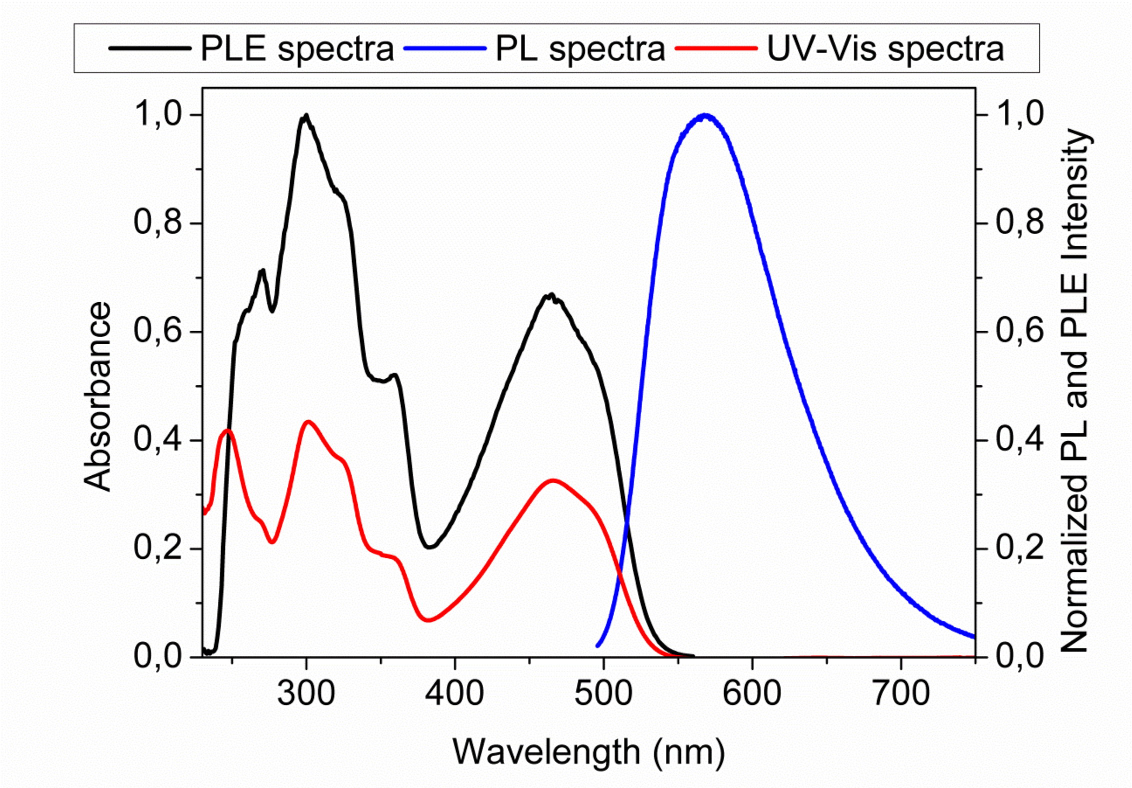

Figure S7. Spectroscopic characterization of compound **27** in $CHCl_3$ solution (10^{-5} mol L^{-1}). Red line: UV-Vis, Bue line: Normalized PL spectra, Black line: Normalized PLE spectra.

3.1 CV spectra of compounds **24-27**

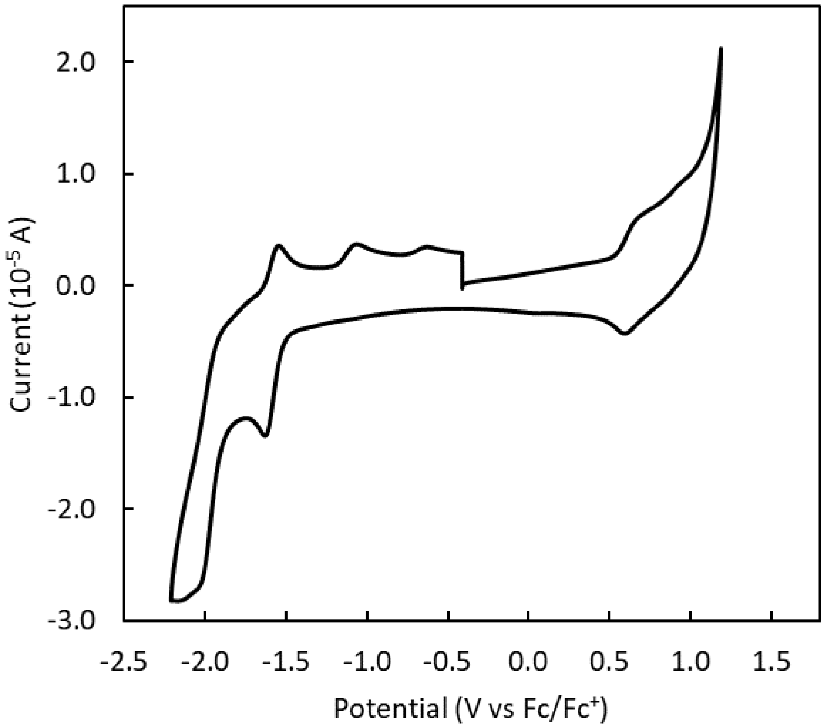

Figure S8. Cyclic Voltammogram of **24** in 0.1 M $TBAPF_6$ CH_2Cl_2.

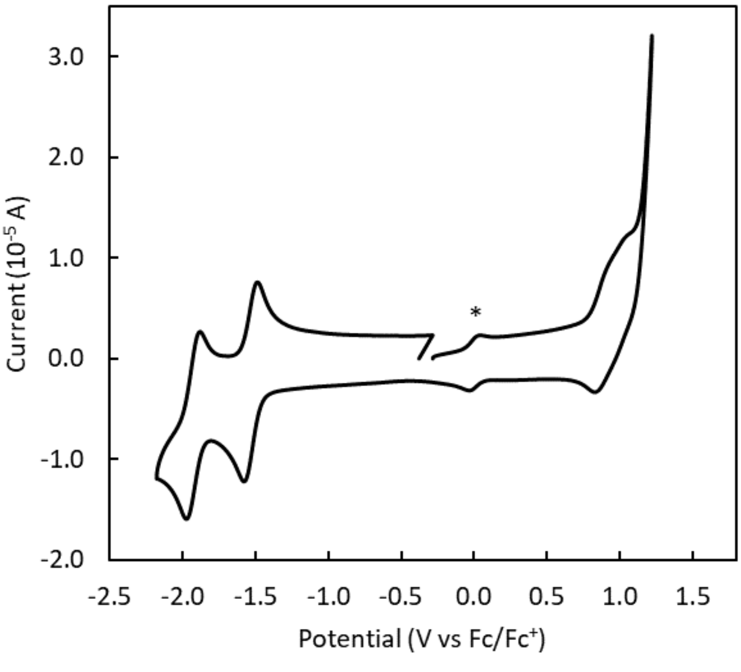

Figure S9. Cyclic Voltammogram of **25** in 0.1 M $TBAPF_6$ in 1:4 MeCN:*o*-DCB. The asterisk (*) denotes the Fc/Fc^+ redox couple.

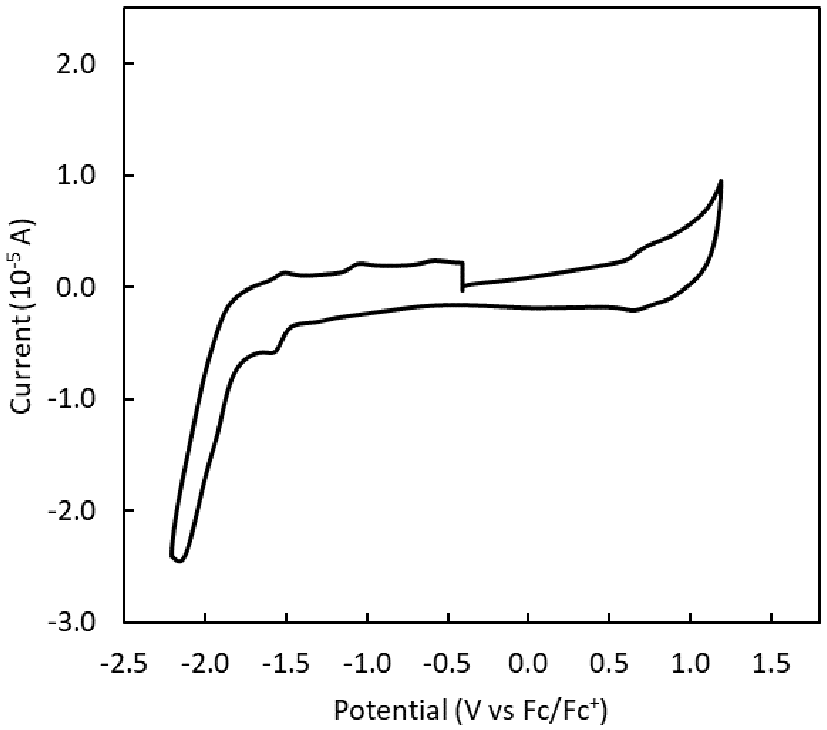

Figure S10. Cyclic Voltammogram of **26** in 0.1 M $TBAPF_6$ in CH_2Cl_2.

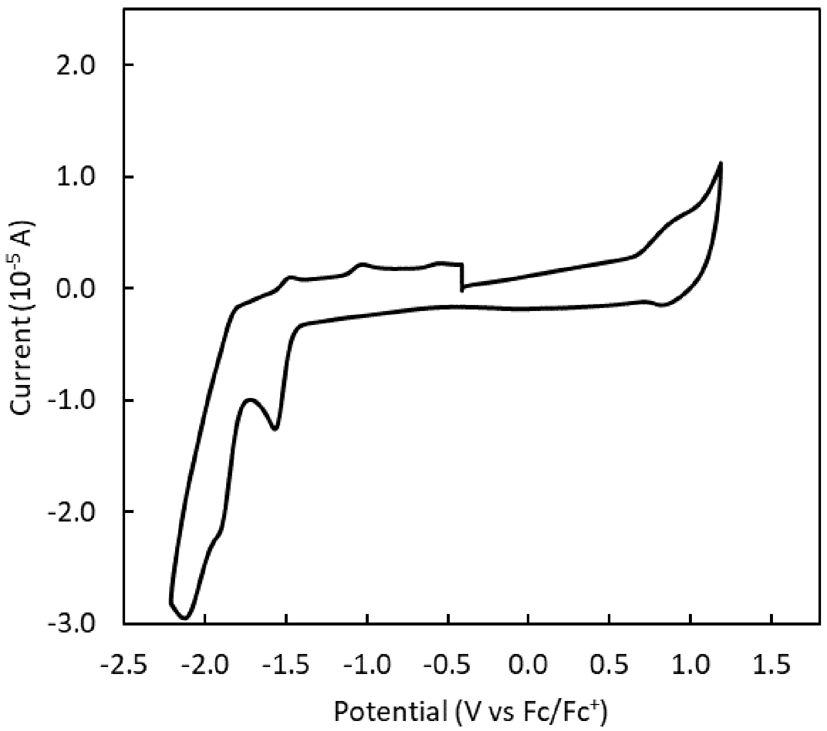

Figure S11. Cyclic Voltammogram of **27** in 0.1 M $TBAPF_6$ in CH_2Cl_2.

4. References

[1] Pommerehne, J.; Vestweber, H.; Guss, W.; Mahrt, R. F.; Bässler, H.; Porsch, M.; Daub, J. *Adv. Mater.* **1995**, *7*, 551-554.

[2] Burroughs, L.; Eccleshare, L.; Ritchie, Jo.; Kulkarni, O.; Lygo, B.; Woodward, S.; Lewis, W. *Angew. Chem. Int. Ed.* **2015**, *54*, 10648-10651.

[3] O. Hall, A.; Lee, S. R.; Bootsma, A. N.; Bloom, J. W. G.; Wheeler, S. E.; McNeil, A. J. *J. Polym Sci Part A Polym Chem.* **2017**, *55*, 1530-1535.

[4] Sankar, E.; Raju, P.; Karunakaran, J.; Mohanakrishnan, A. K. *J. Org. Chem.* **2017**, *82*, 13583-13593.

[5] Levine, D. R.; Siegler, M. A.; Tovar, J. D. *J. Am. Chem. Soc.* **2014**, *136*, 7132-7139.

5. Spectra of New Compounds

Compound **8**

^{1}H NMR (300 MHz, $CDCl_3$)

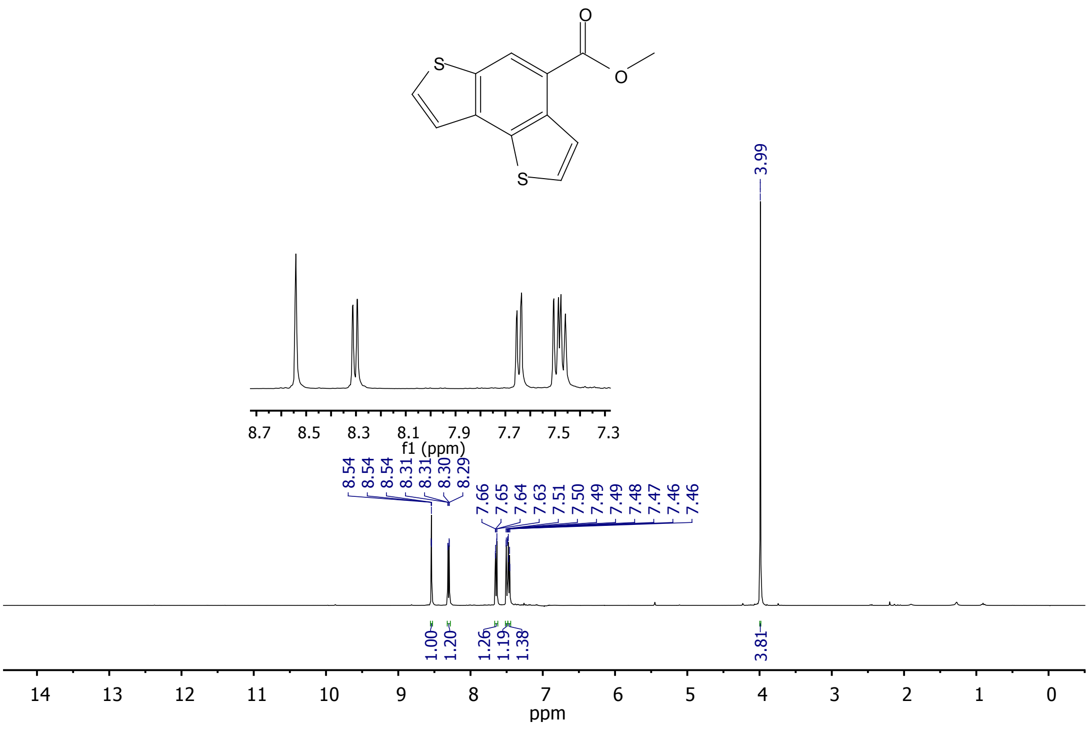

^{13}C NMR (90 MHz, $CDCl_3$)

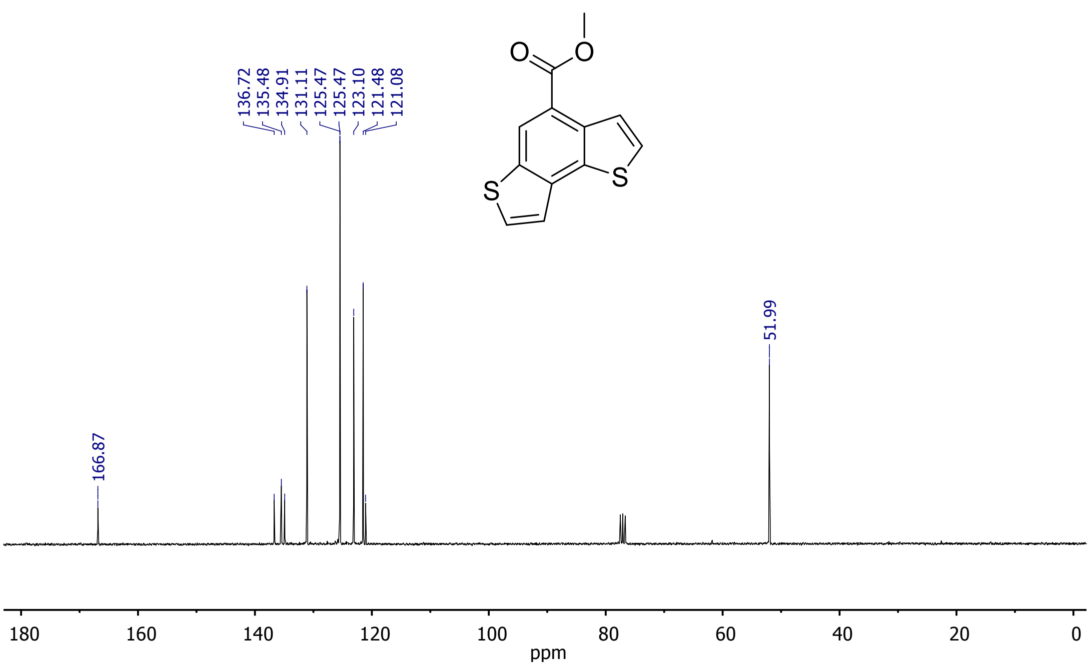

DE-MS

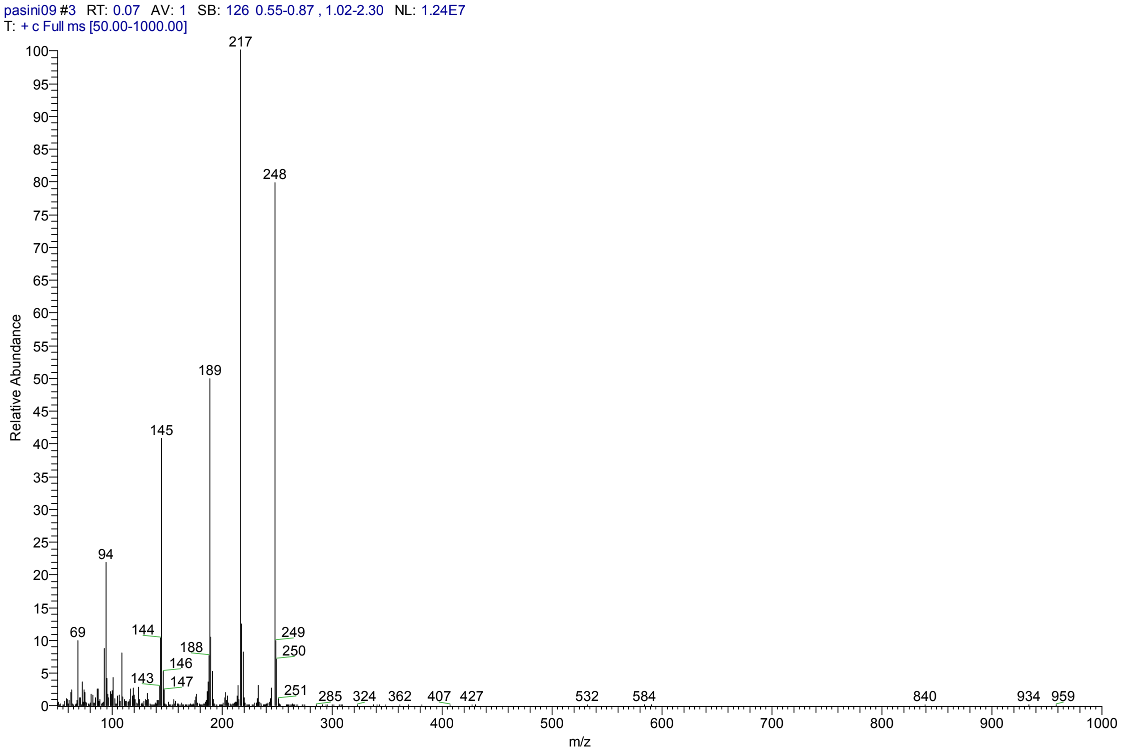

pasini09 #3 RT: 0.07 AV: 1 SB: 126 0.55-0.87 , 1.02-2.30 NL: 1.24E7
T: + c Full ms [50.00-1000.00]
Relative Abundance
m/z
217
248
189
145
94
69
144
143
188
146
147
249
250
251
285
324
362
407
427
532
584
840
934
959

Compound **9**

^{1}H NMR (300 MHz, DMSO-d_6)

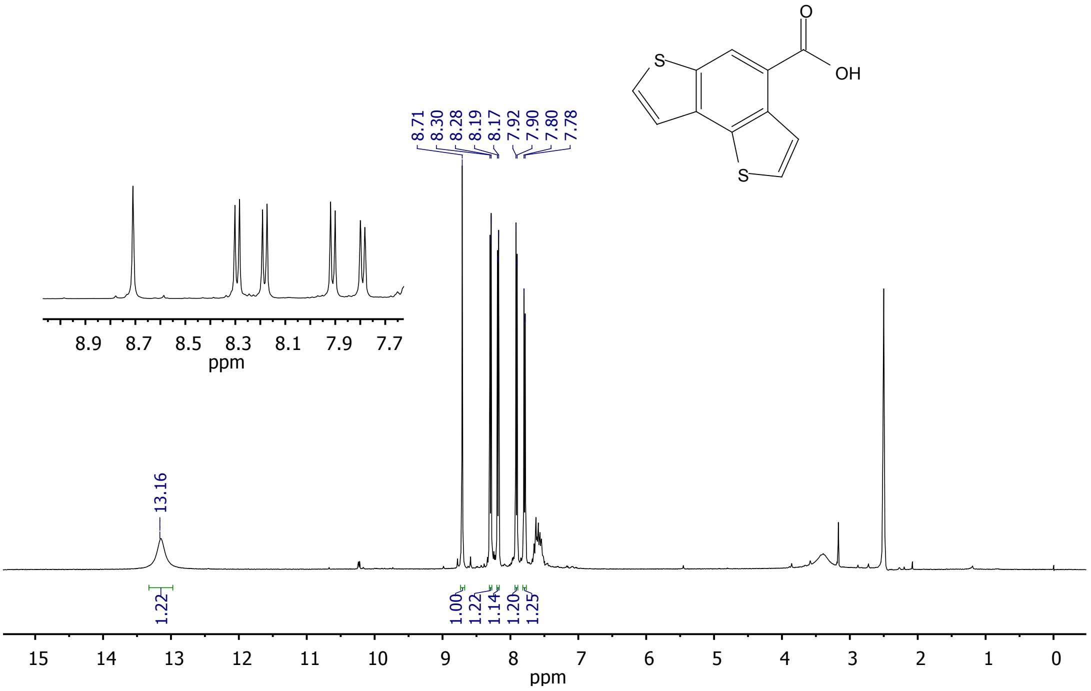

^{13}C NMR (90 MHz, DMSO-d_6)

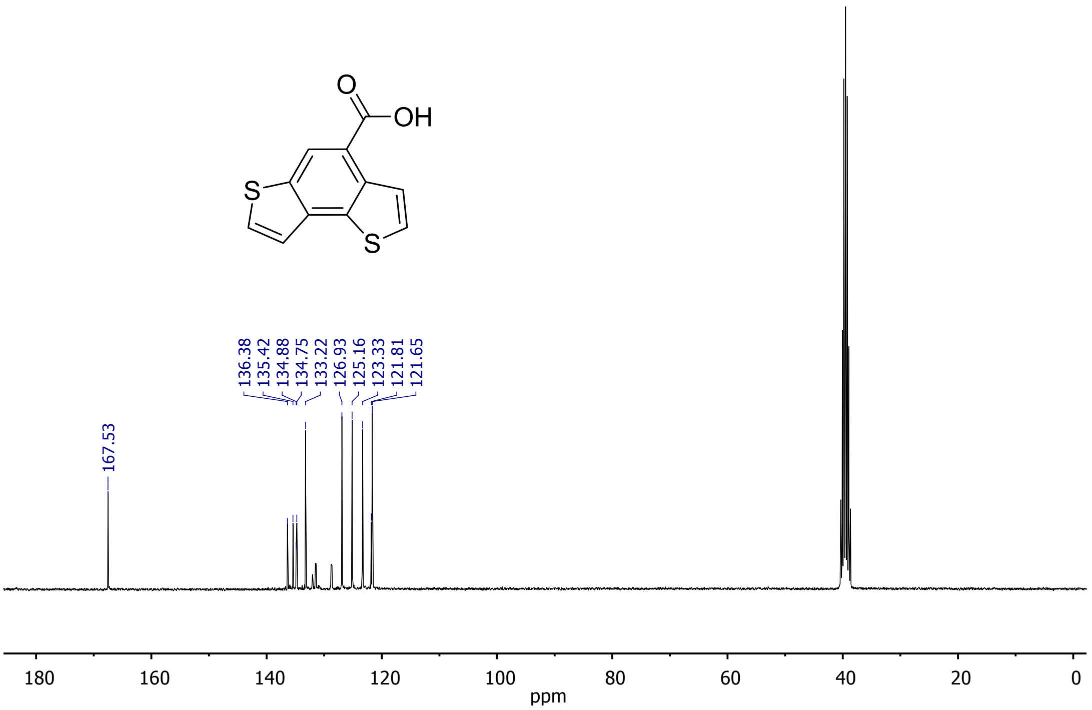

ESI-MS

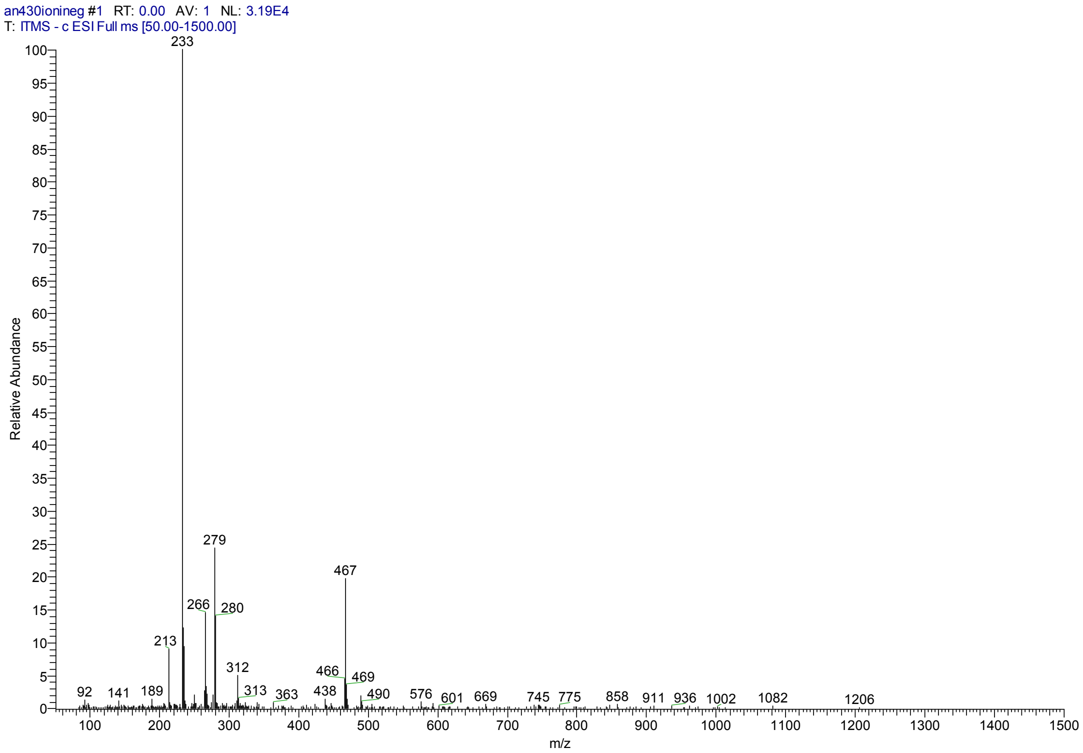
an430ionineg #1 RT: 0.00 AV: 1 NL: 3.19E4
T: ITMS - c ESI Full ms [50.00-1500.00]
Relative Abundance
m/z
233
279
467
266
280
213
312
466
469
92
141
189
313
363
438
490
576
601
669
745
775
858
911
936
1002
1082
1206

Compound **10**

^{1}H NMR (300 MHz, $CDCl_3$)

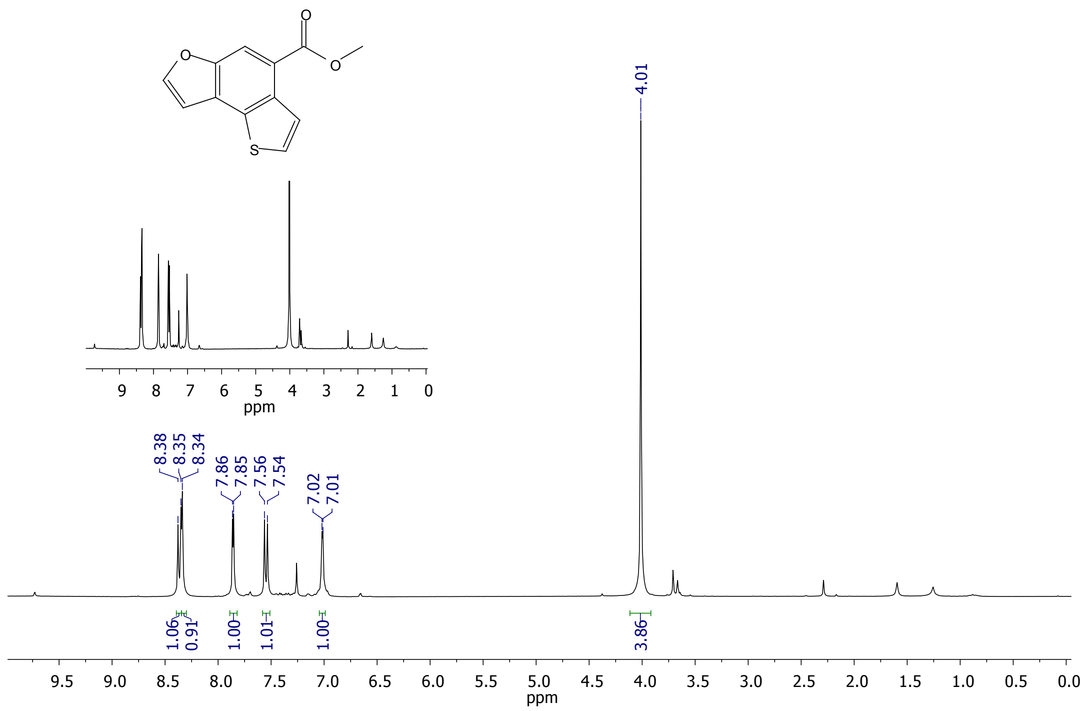

^{13}C NMR (75 MHz, $CDCl_3$)

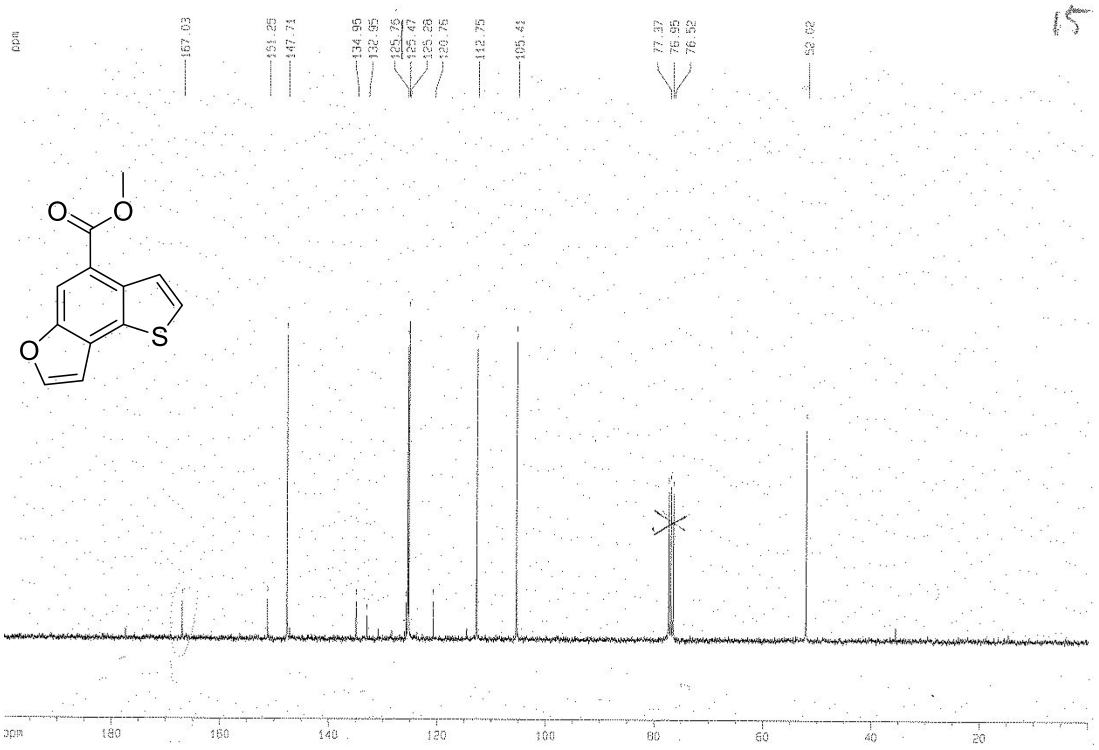

COSY

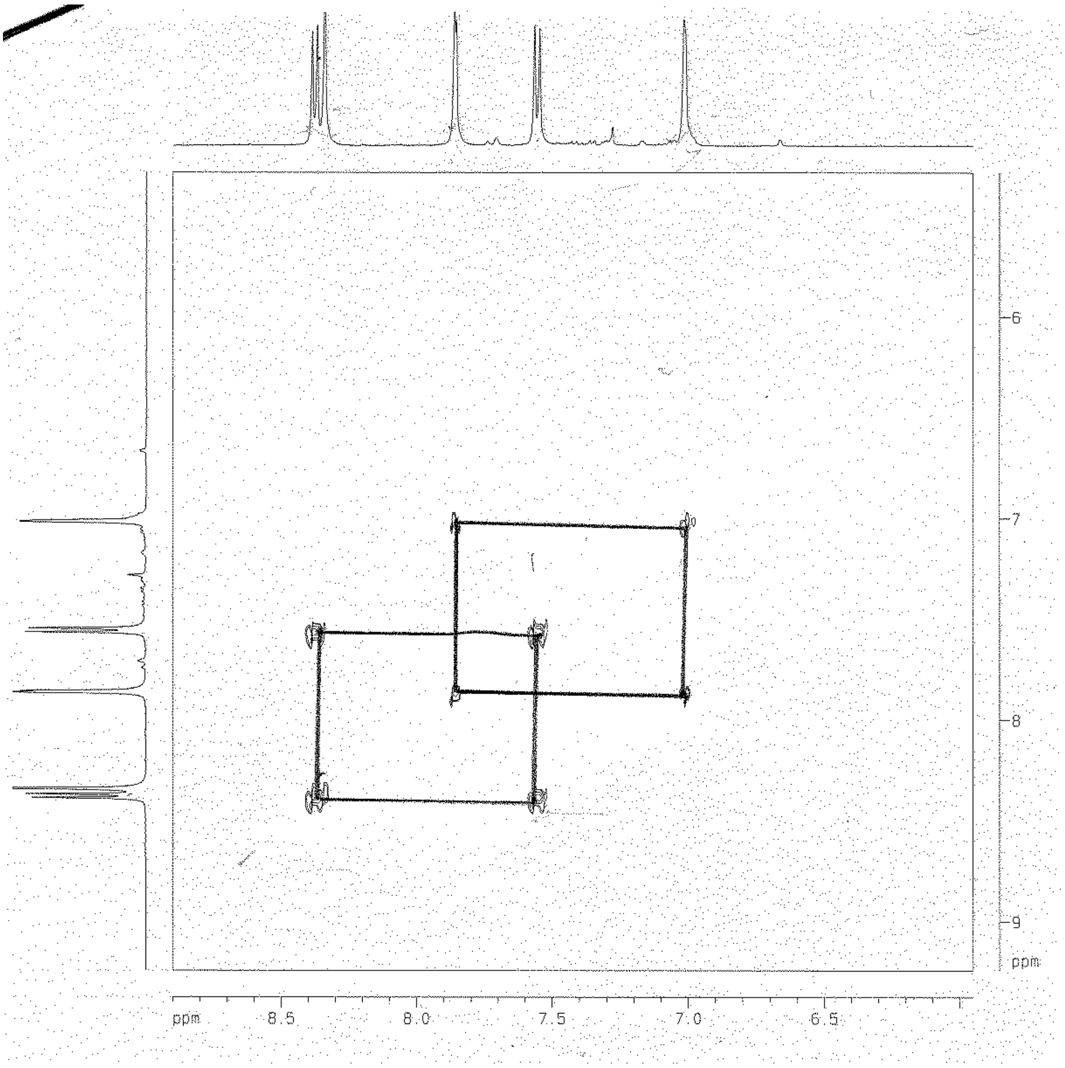

6
7
8
9
ppm
ppm
8.5
8.0
7.5
7.0
6.5

DE-MS

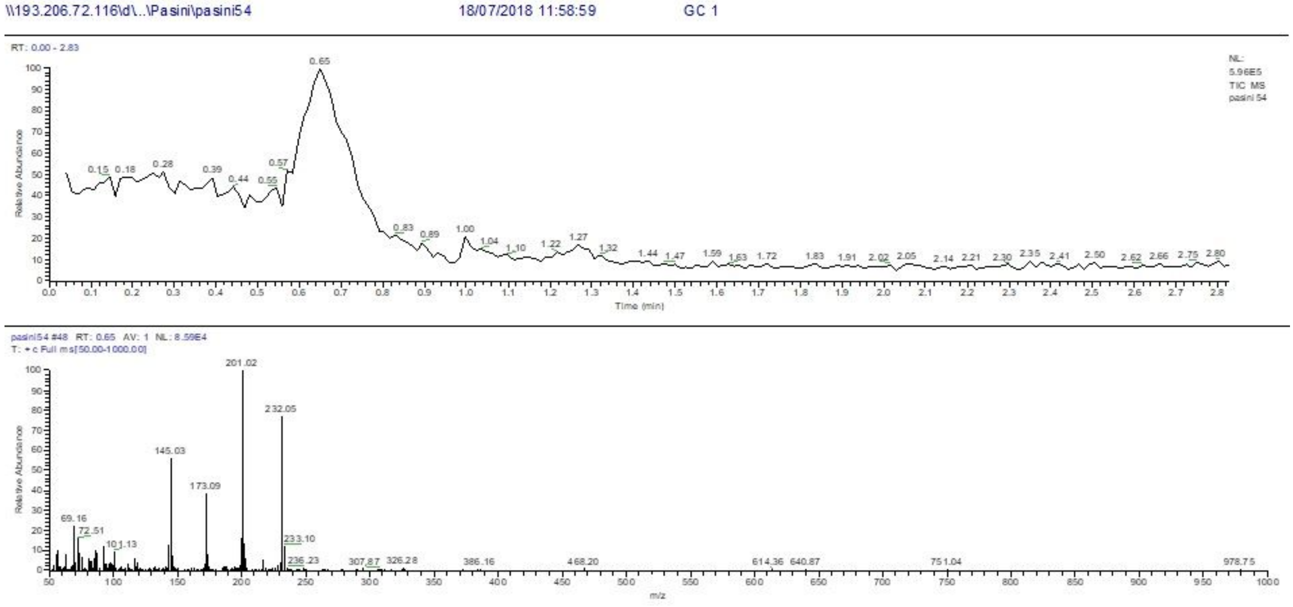

\\193.206.72.116\d\..\Pasini\pasini54
18/07/2018 11:58:59
GC 1
RT: 0.00 - 2.83
NL: 5.96E5 TIC MS pasini 54
Relative Abundance
Time (min)
0.65
pasini54 #48 RT: 0.65 AV: 1 NL: 8.59E4
T: + c Full ms [50.00-1000.00]
Relative Abundance
m/z
201.02
232.05
145.03
173.09
69.16
72.51
101.13
233.10
236.23
307.87
326.28
386.16
468.20
614.36
640.87
751.04
978.75

Compound **11**

¹H NMR (200 MHz, DMSO-d_6)

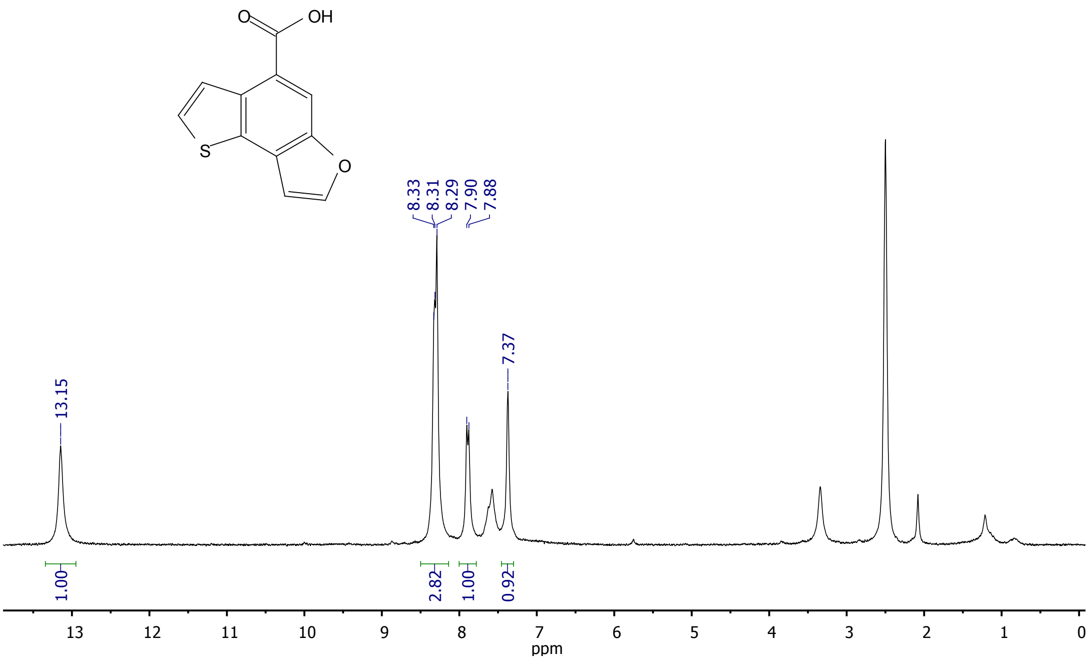

¹³C NMR (90 MHz, DMSO-d_6)

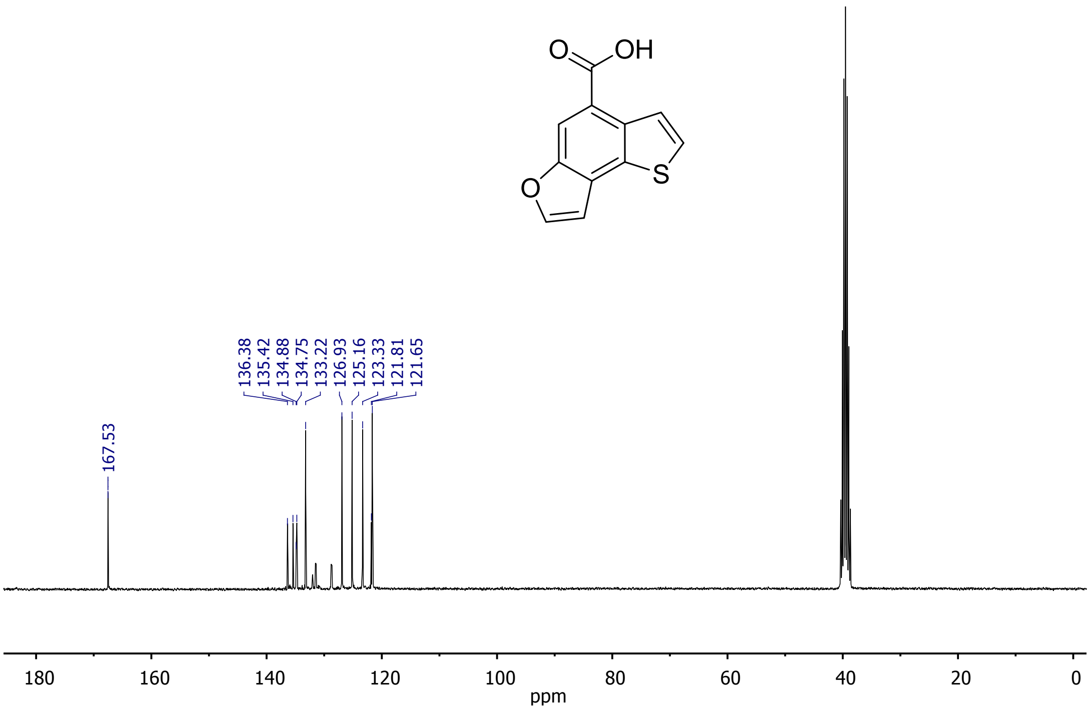

ESI-MS

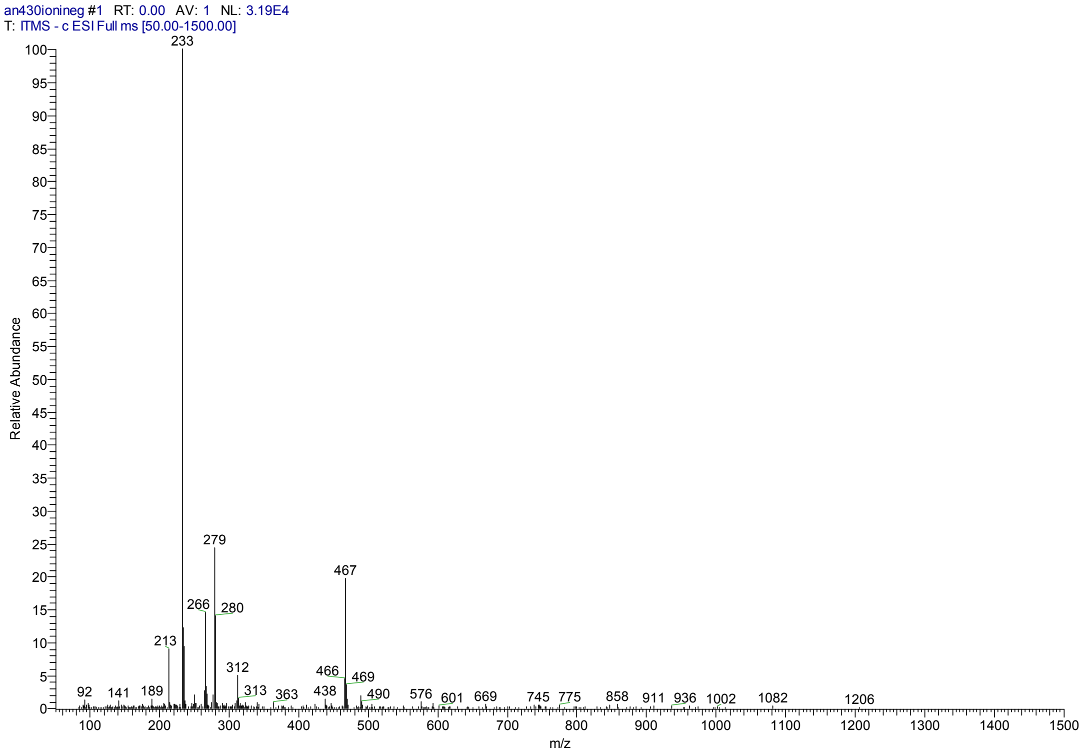
an430ionineg #1 RT: 0.00 AV: 1 NL: 3.19E4
T: ITMS - c ESI Full ms [50.00-1500.00]
Relative Abundance
m/z
233
279
467
266
280
213
312
466
469
92
141
189
313
363
438
490
576
601
669
745
775
858
911
936
1002
1082
1206

Compound **12**

^{1}H NMR (300 MHz, CDCl$_3$)

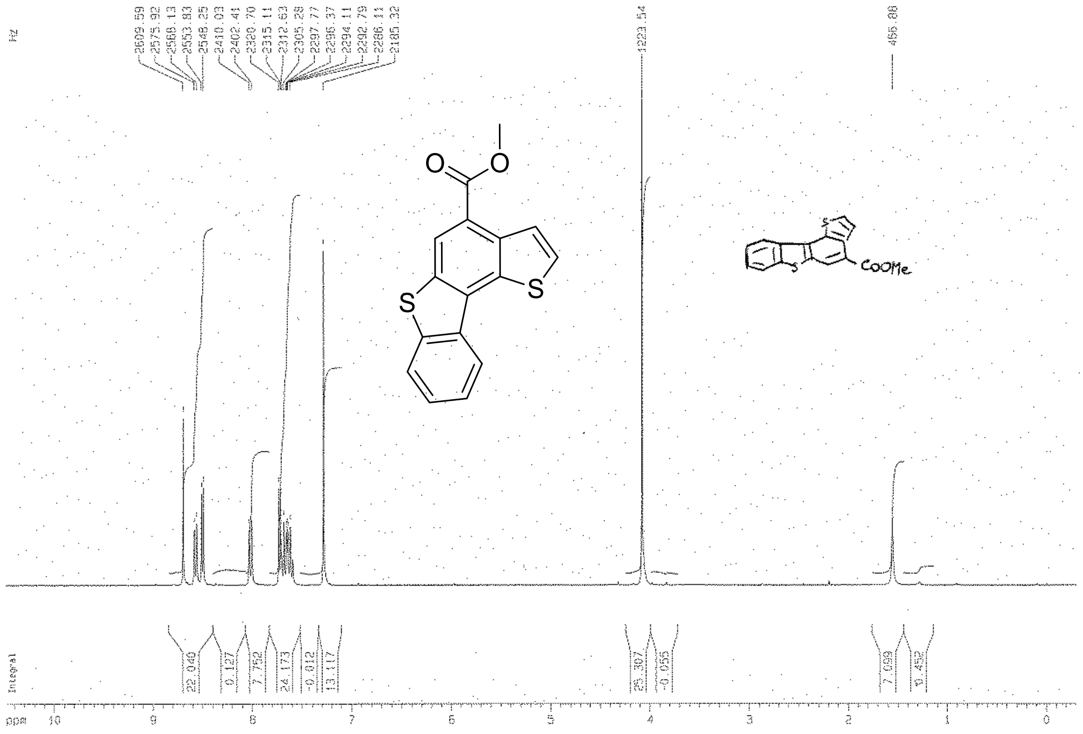

^{13}C NMR (75 MHz, CDCl$_3$)

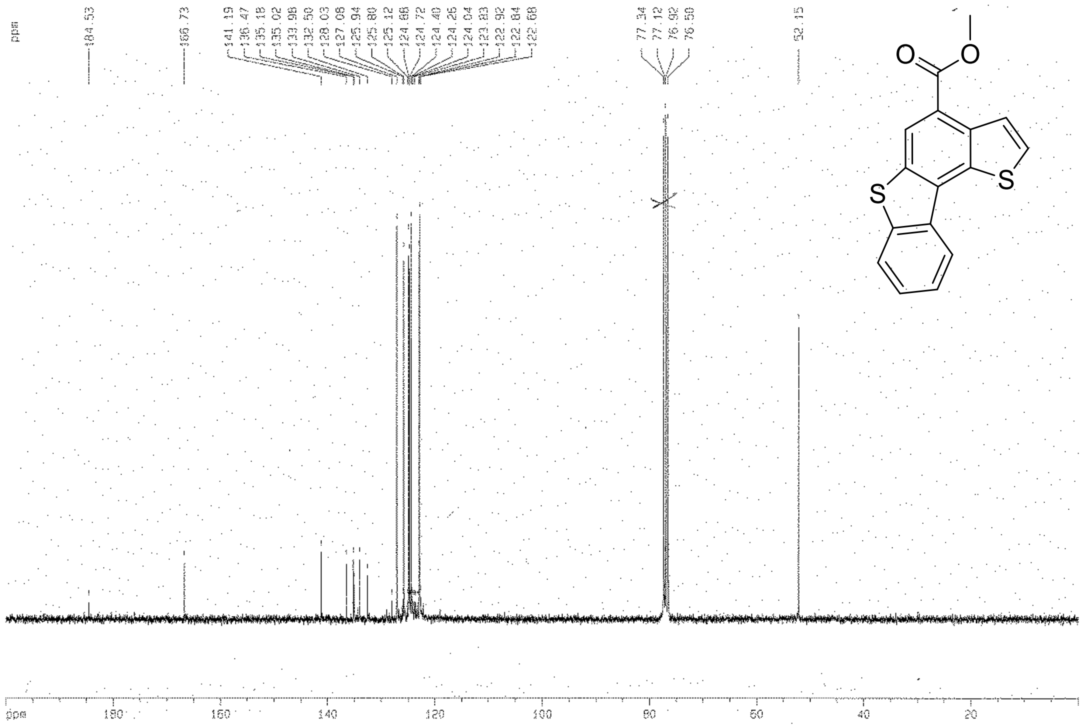

Zoom of aromatic region

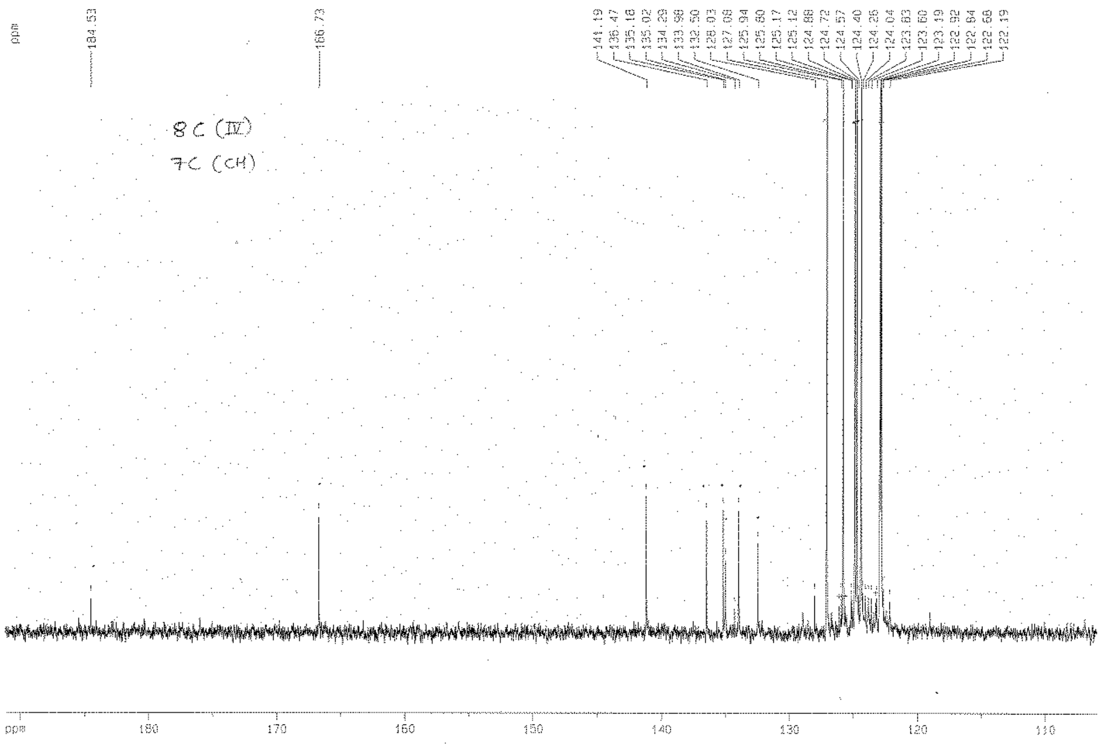

DE-MS

\\193.206.72.116\d\...\Pasini\pasini52 18/07/2018 11:36:27 GC 17

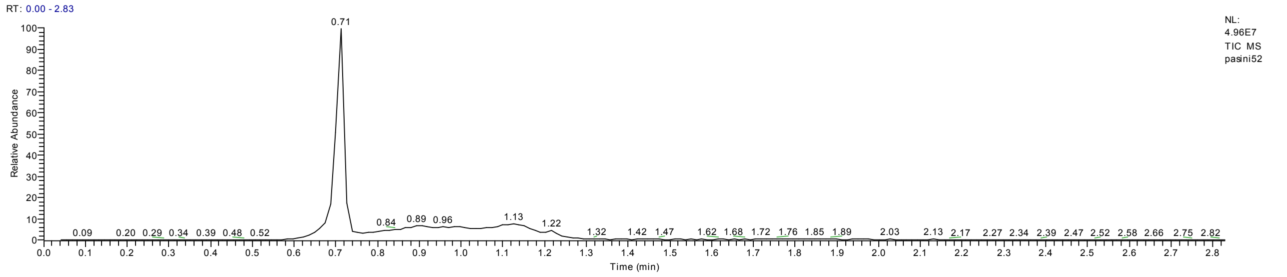

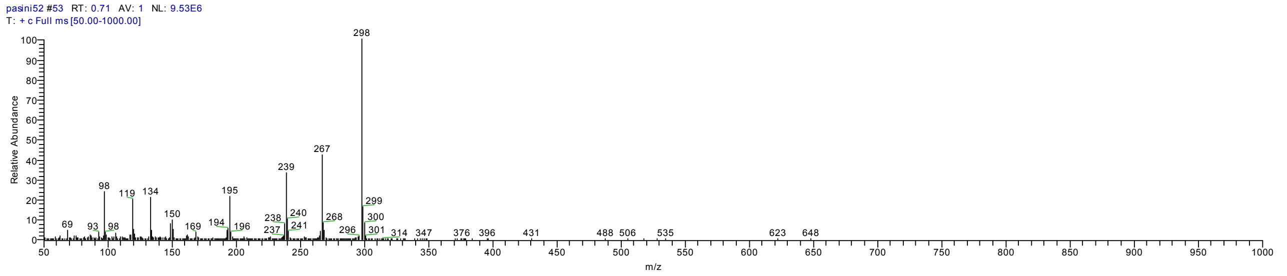

Compound **13**

^{1}H NMR (200 MHz, $CDCl_3$)

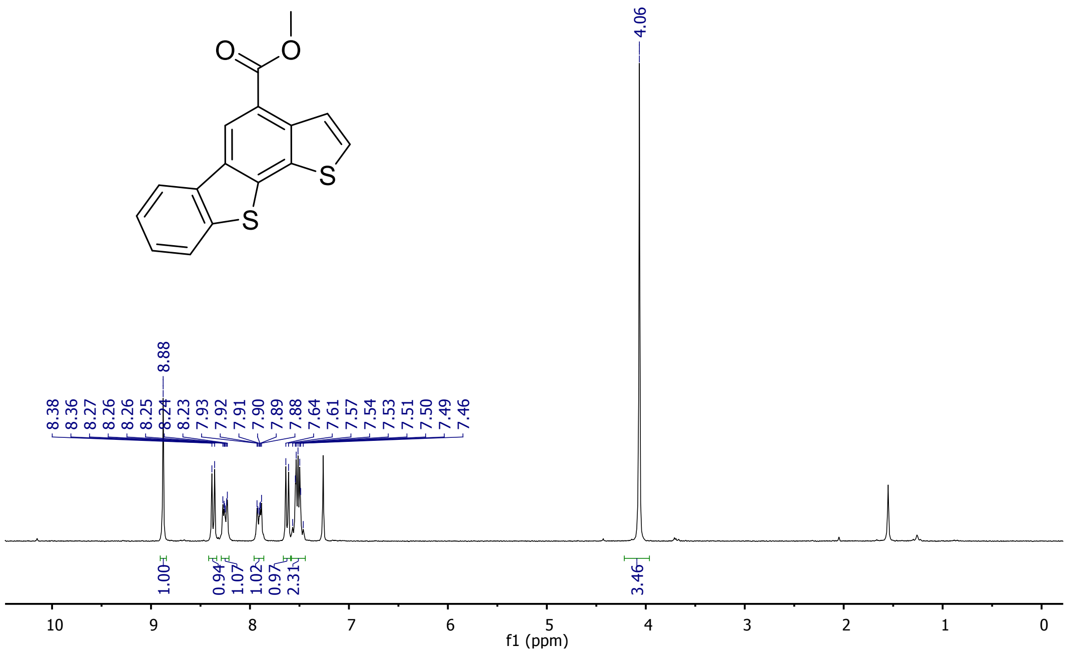

^{13}C NMR (75 MHz, $CDCl_3$)

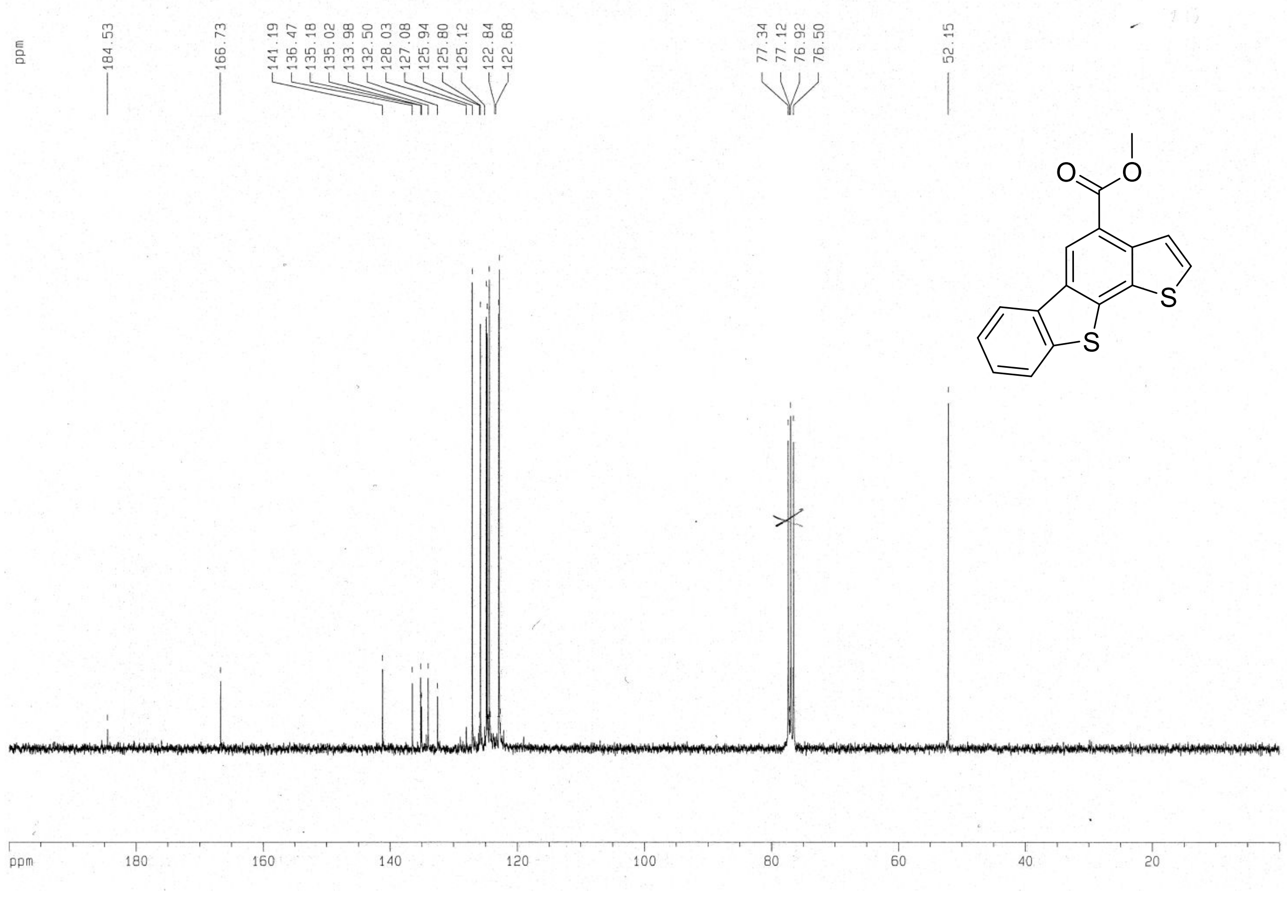

DE-MS

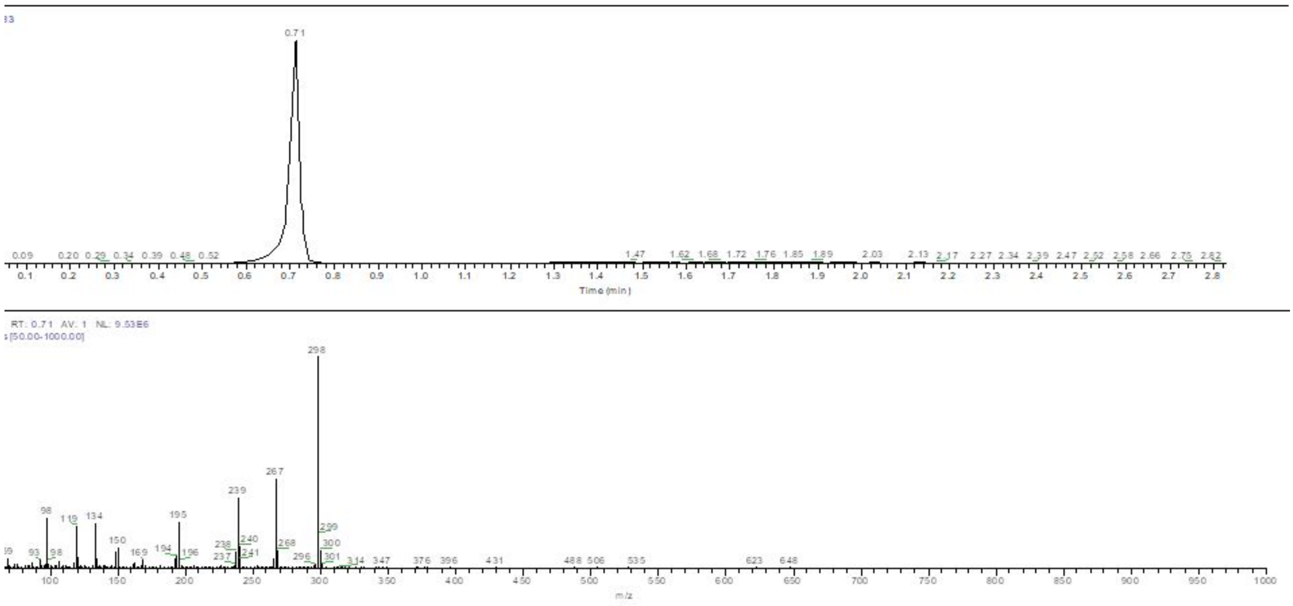

Compound **14**

^{1}H NMR (300 MHz, CDCl$_3$)

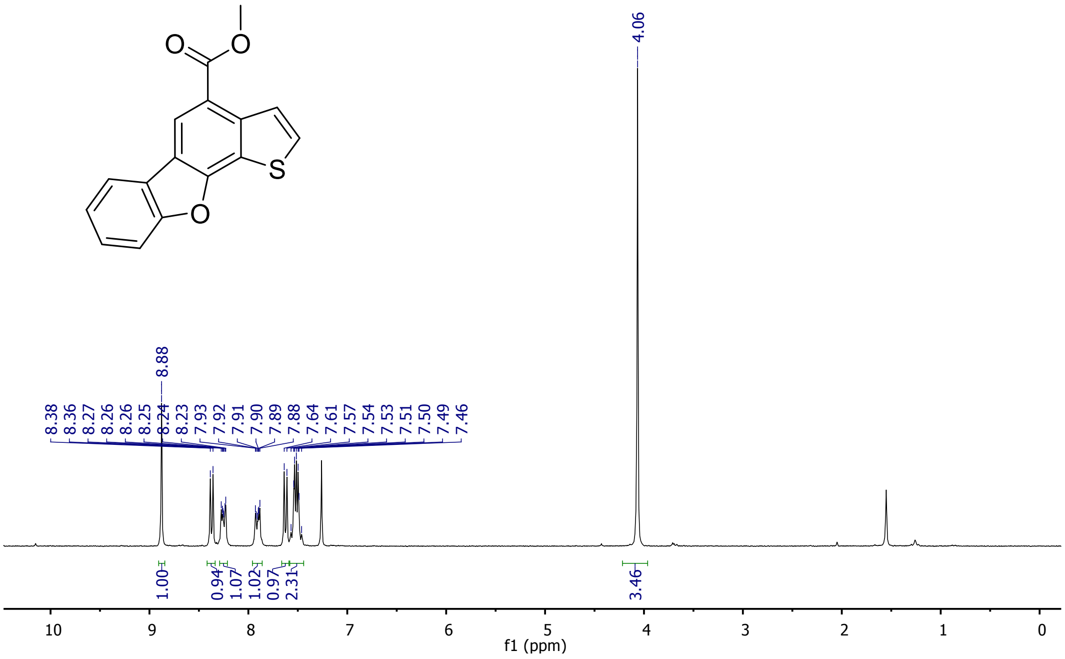

^{13}C NMR (90 MHz, CDCl$_3$)

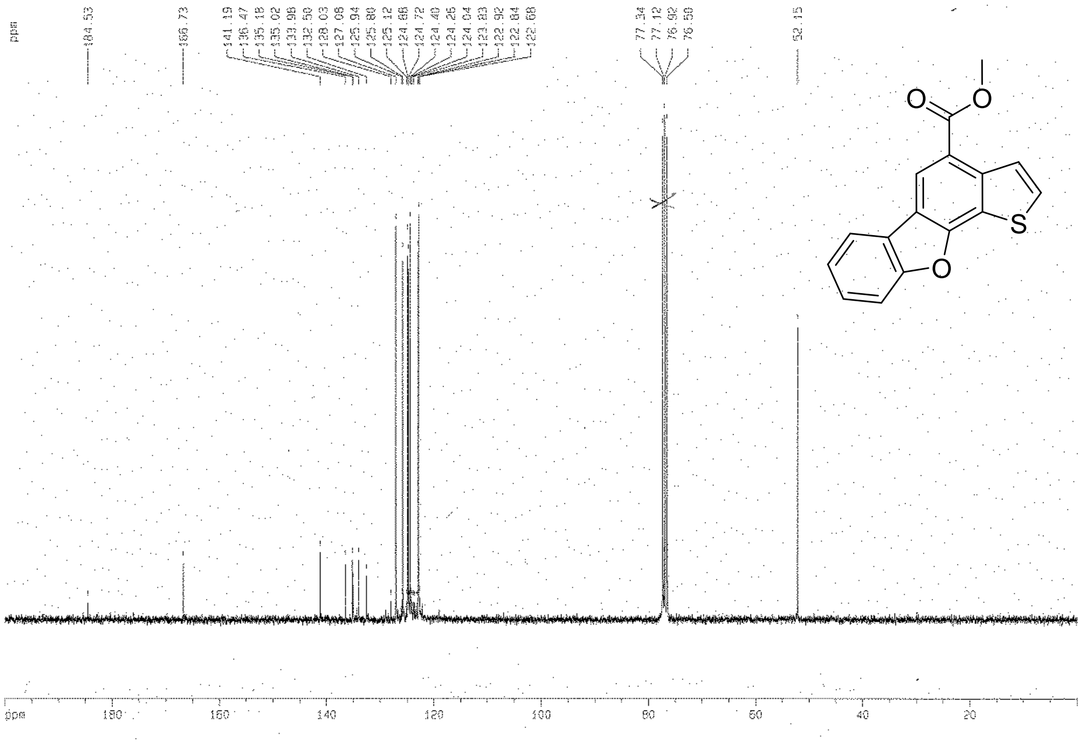

Compound **15**

^{1}H NMR (300 MHz, CDCl$_3$)

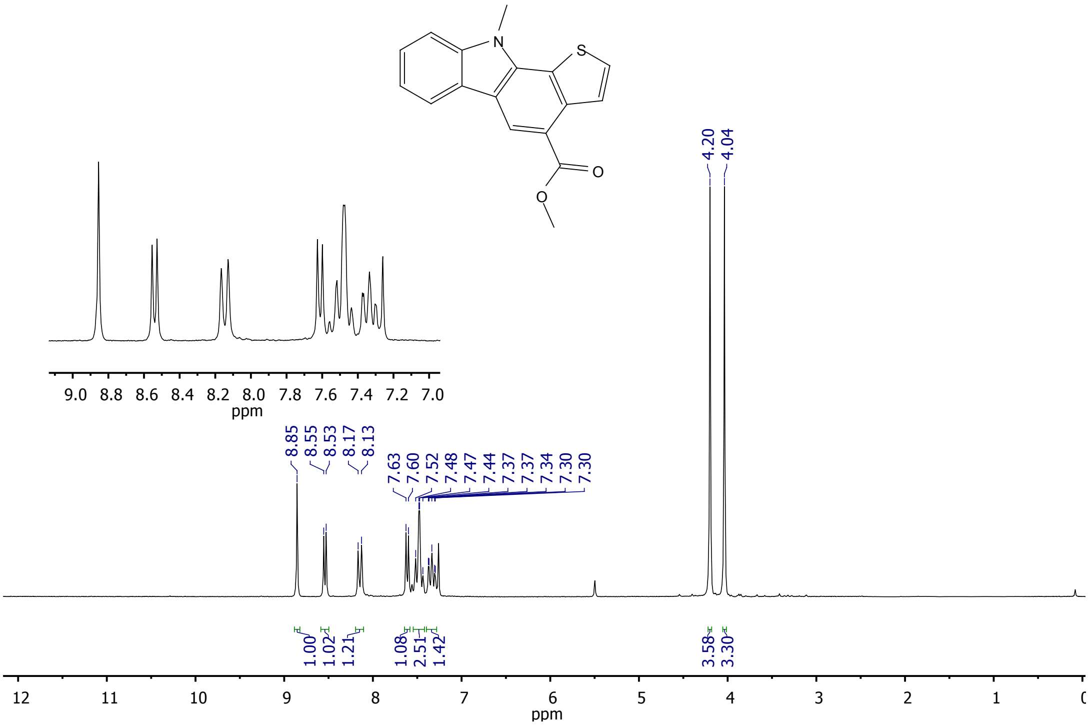

^{13}C NMR (75 MHz, CDCl$_3$)

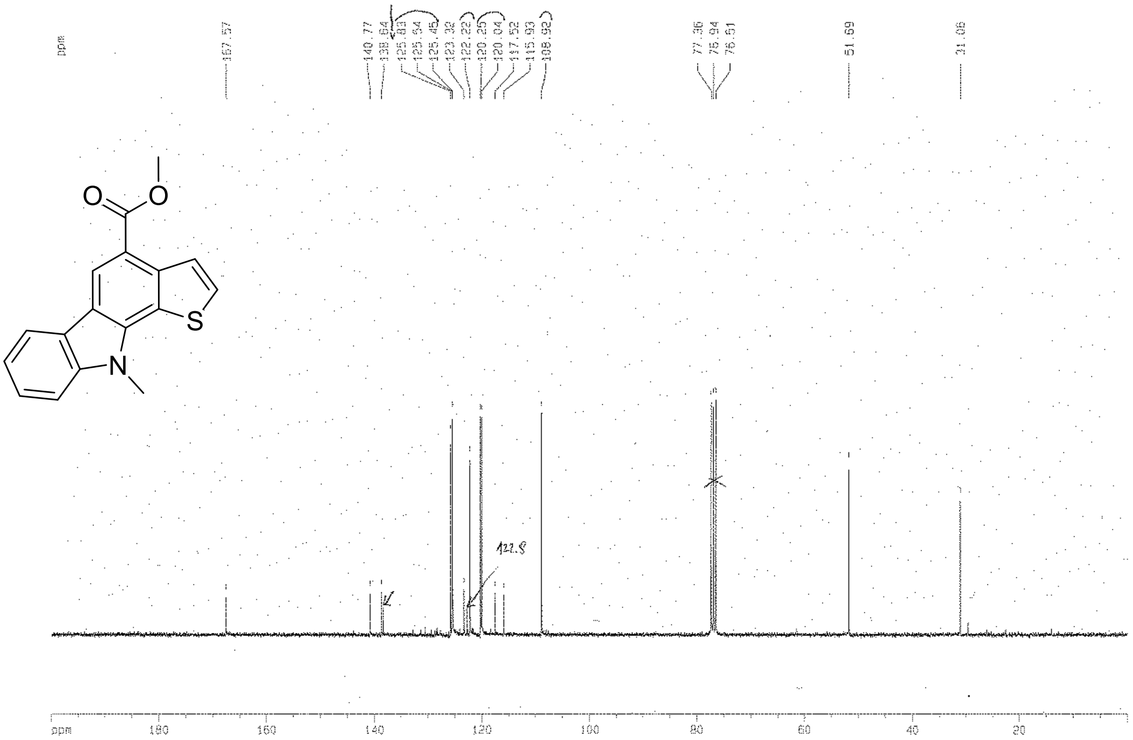

DEPT

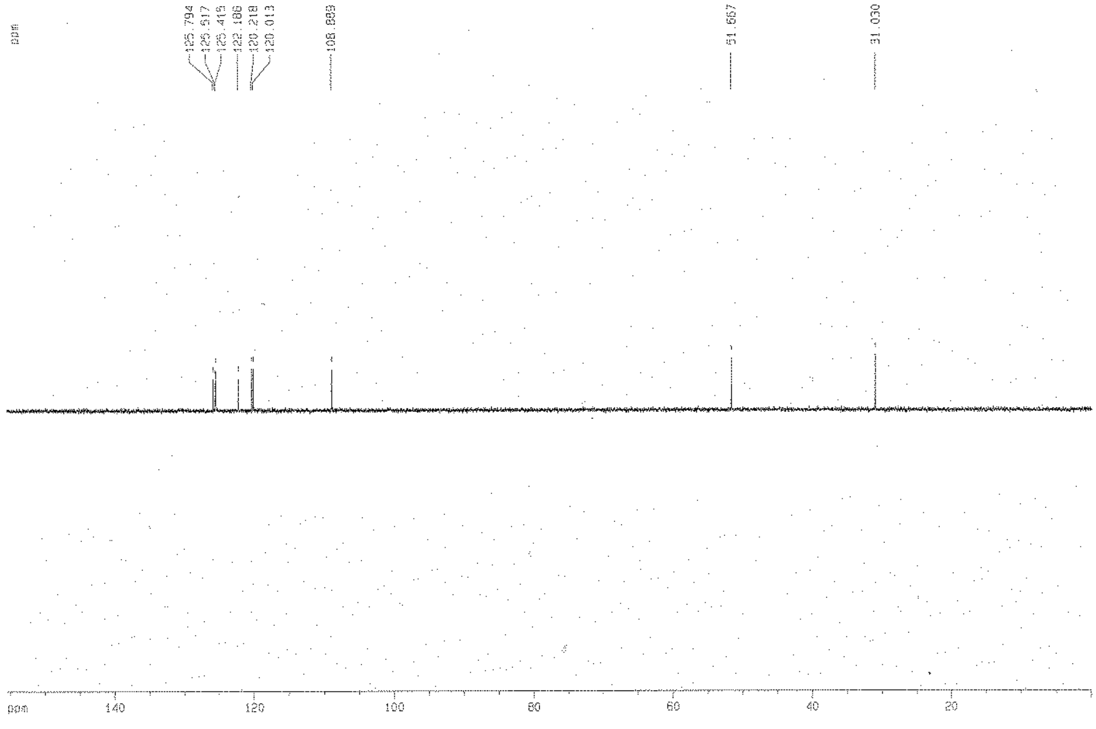
ppm
125.794
125.517
125.419
122.186
120.218
120.013
108.889
51.667
31.030
ppm
140
120
100
80
60
40
20

ESI-MS

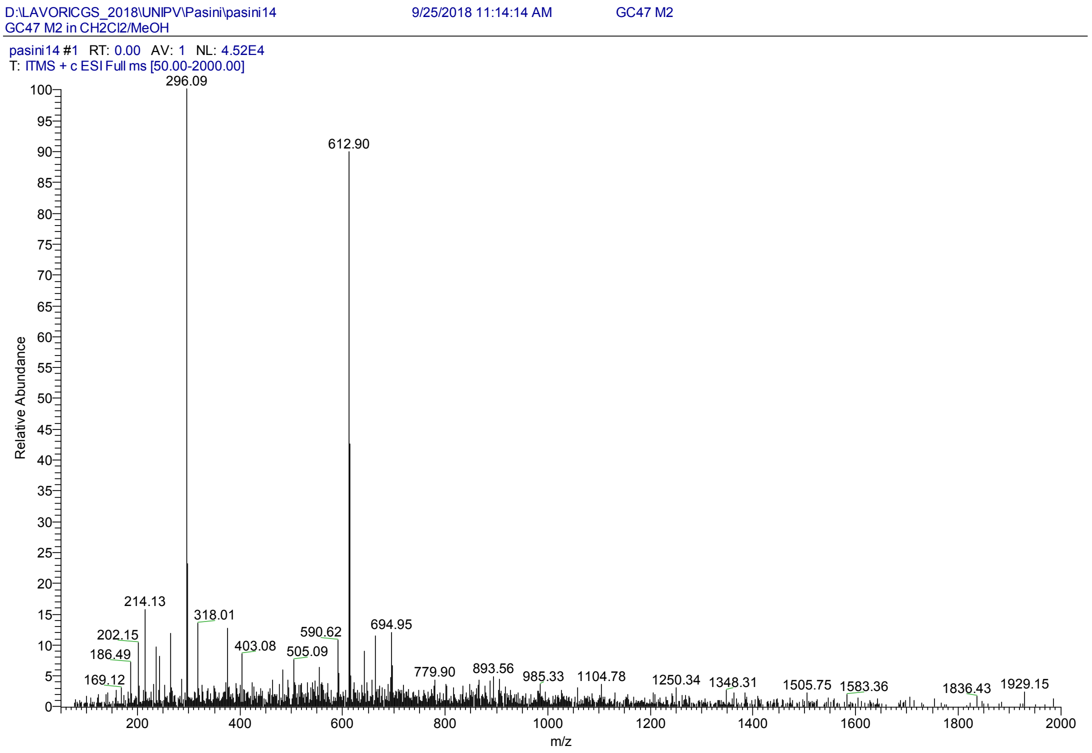
D:\LAVORICGS_2018\UNIPV\Pasini\pasini14
9/25/2018 11:14:14 AM
GC47 M2
GC47 M2 in CH2Cl2/MeOH
pasini14 #1 RT: 0.00 AV: 1 NL: 4.52E4
T: ITMS + c ESI Full ms [50.00-2000.00]
Relative Abundance
m/z
296.09
612.90
214.13
318.01
202.15
186.49
169.12
403.08
505.09
590.62
694.95
779.90
893.56
985.33
1104.78
1250.34
1348.31
1505.75
1583.36
1836.43
1929.15

Compound **16**

^{1}H NMR (300 MHz, $CDCl_3$)

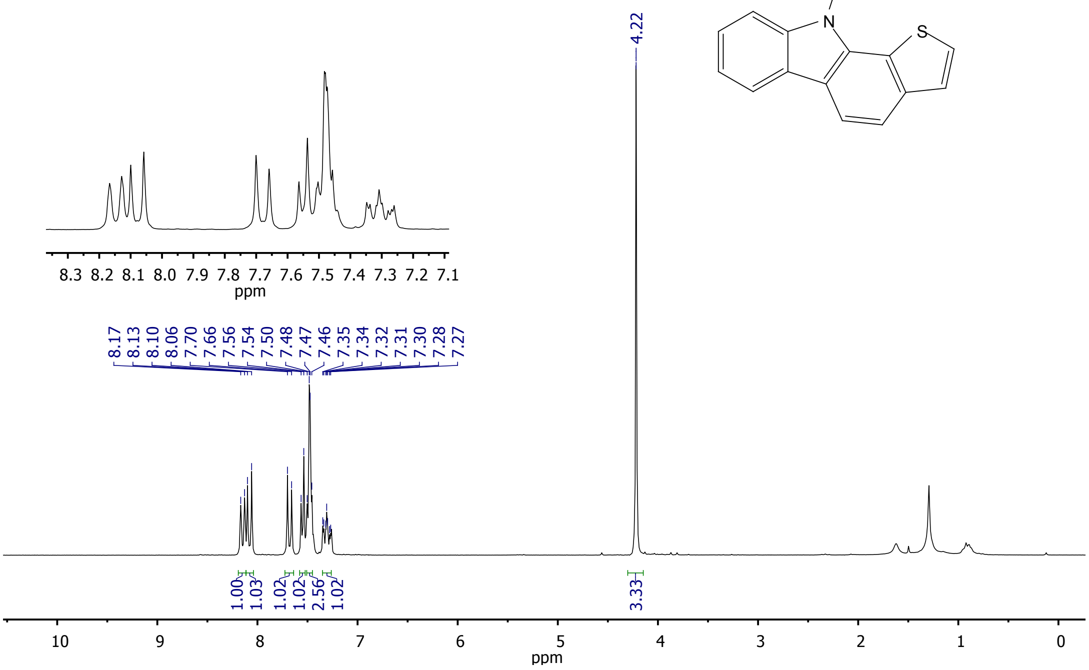

^{13}C NMR (75 MHz, $CDCl_3$)

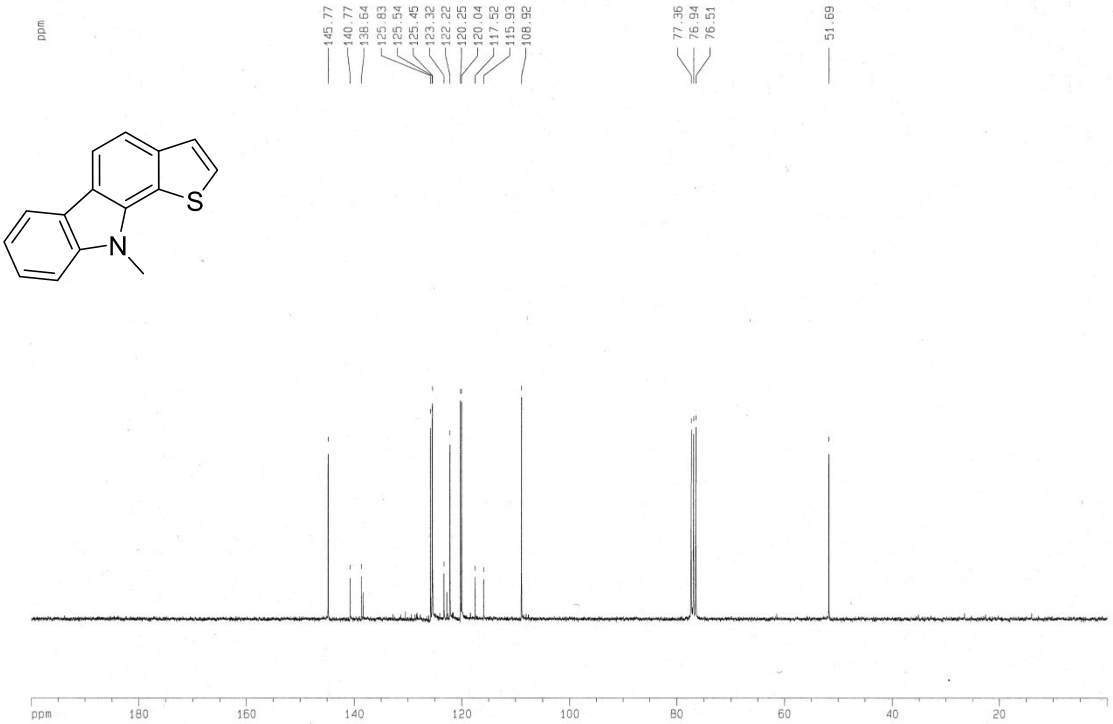

ESI-MS

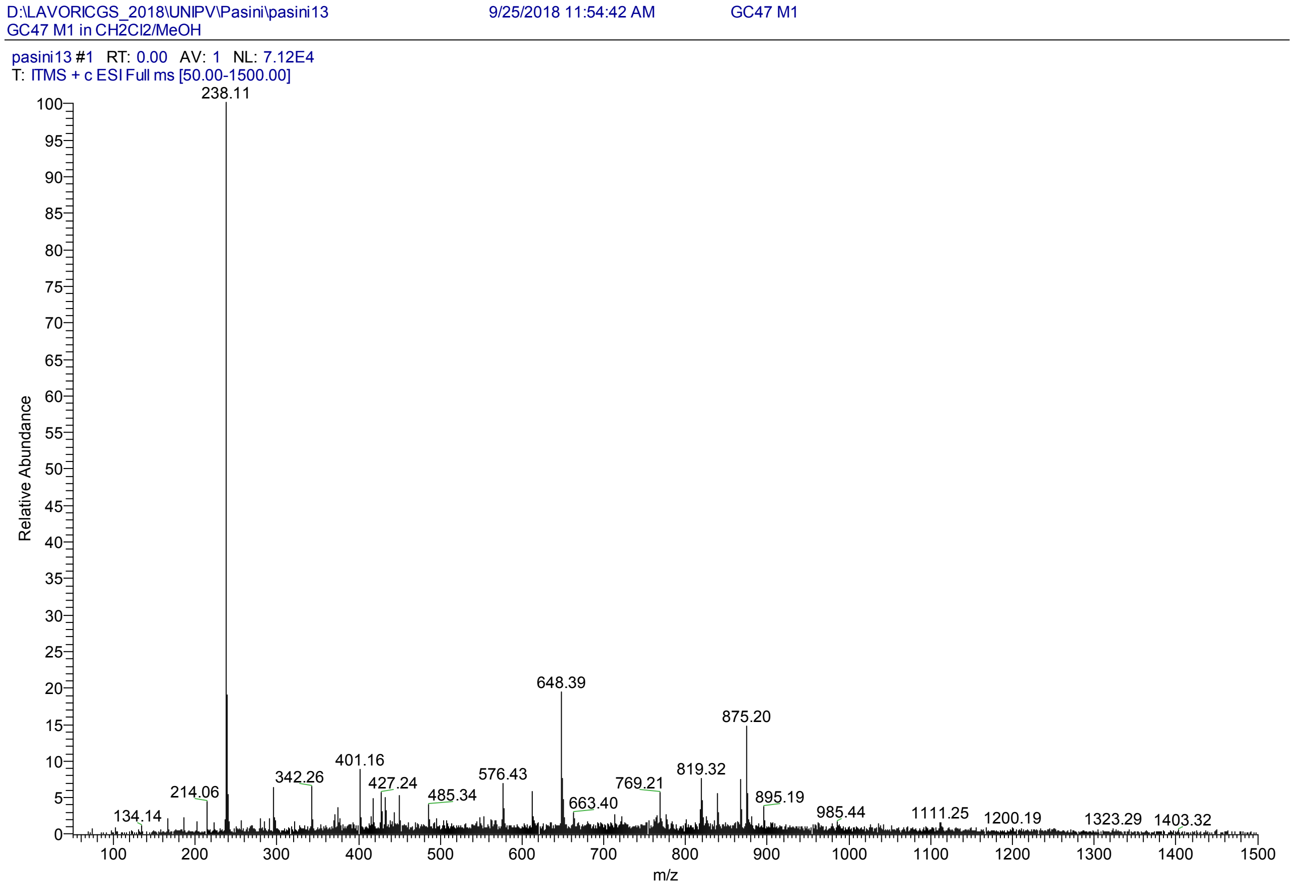
D:\LAVORICGS_2018\UNIPV\Pasini\pasini13
9/25/2018 11:54:42 AM
GC47 M1
GC47 M1 in CH2Cl2/MeOH
pasini13 #1 RT: 0.00 AV: 1 NL: 7.12E4
T: ITMS + c ESI Full ms [50.00-1500.00]
Relative Abundance
m/z
238.11
648.39
875.20
401.16
819.32
576.43
342.26
427.24
769.21
214.06
485.34
895.19
663.40
134.14
985.44
1111.25
1200.19
1323.29
1403.32

Compound **17**

^{1}H NMR (300 MHz, $CDCl_3$)

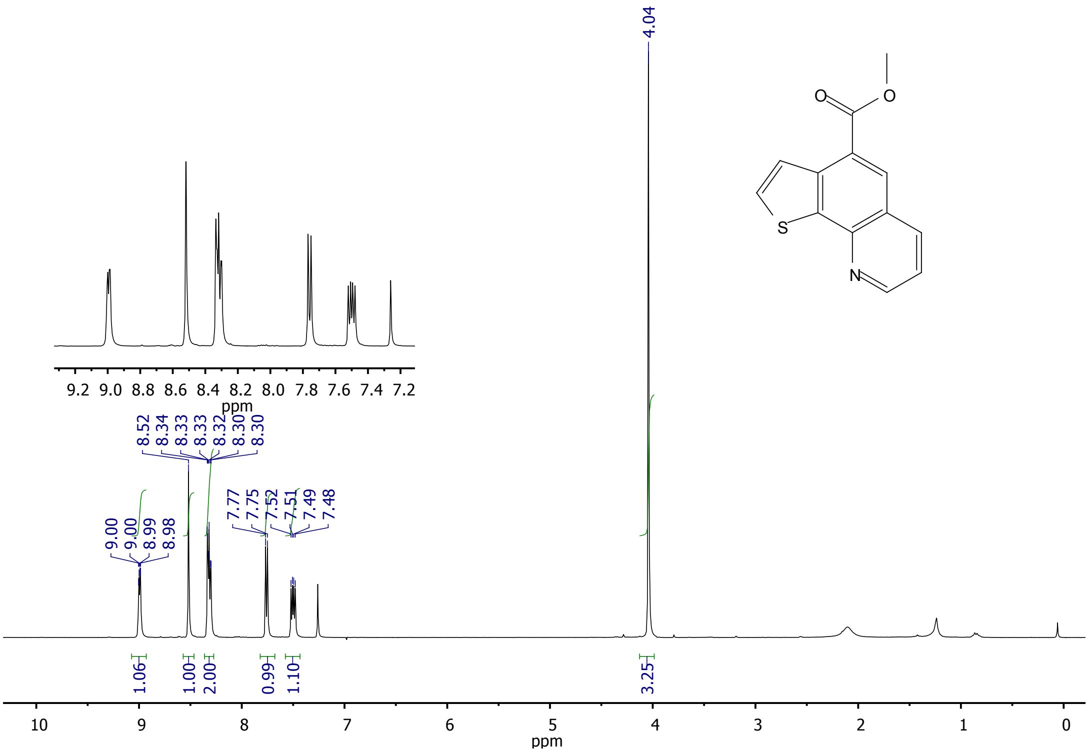

^{13}C NMR (90 MHz, $CDCl_3$)

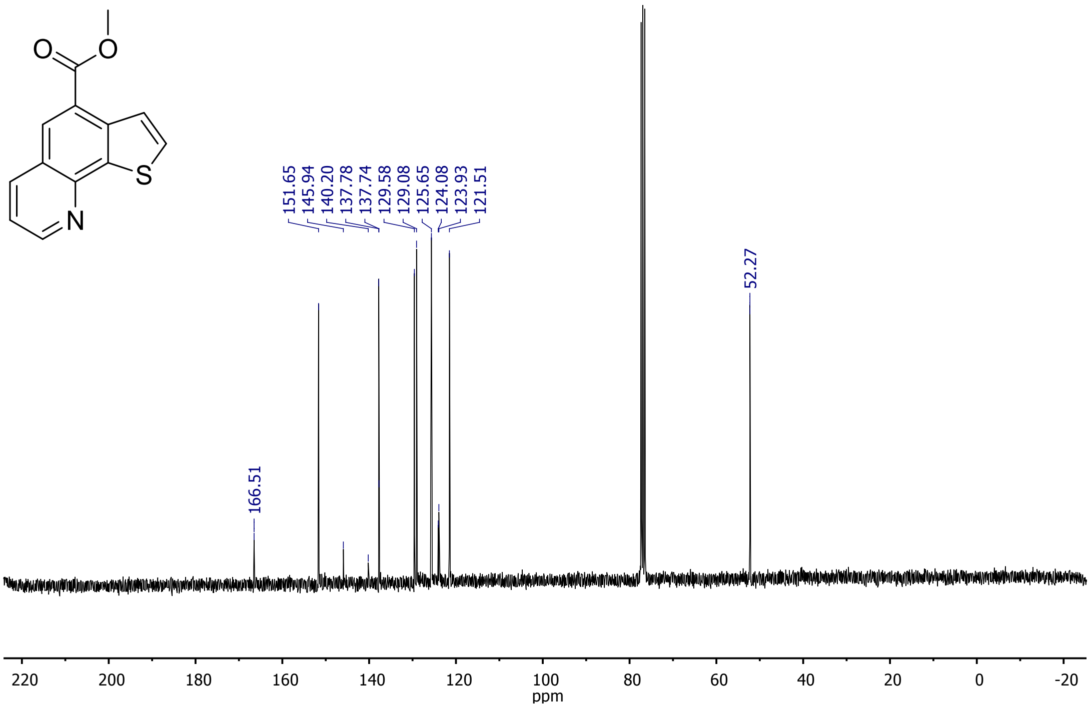

ESI-MS

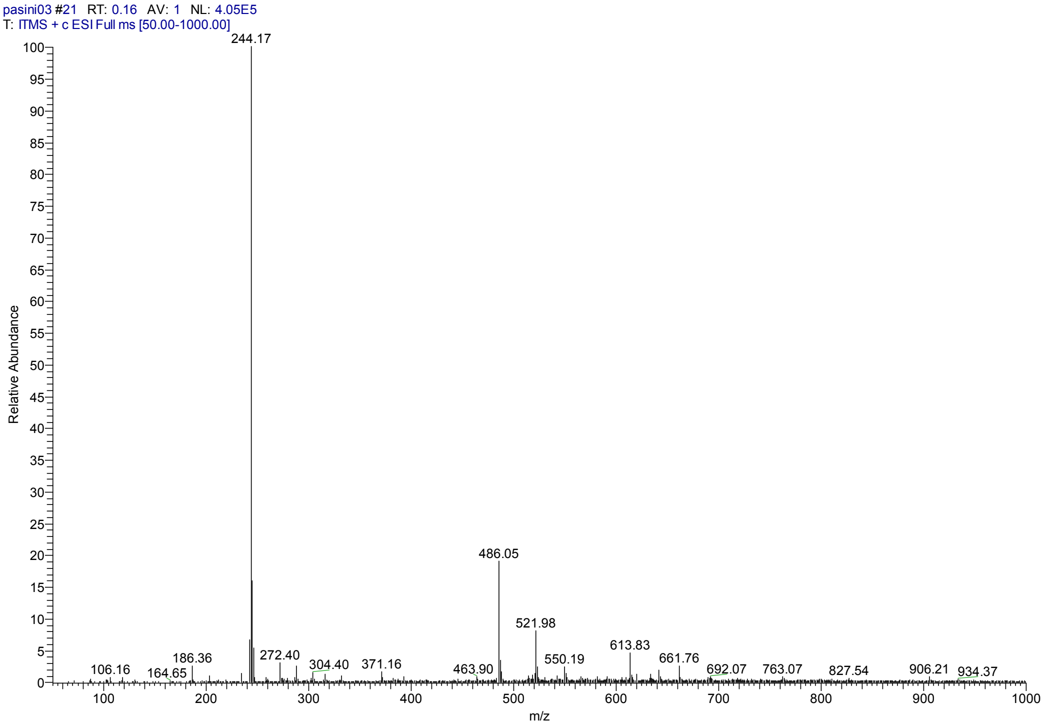

pasini03 #21 RT: 0.16 AV: 1 NL: 4.05E5
T: ITMS + c ESI Full ms [50.00-1000.00]
244.17
486.05
521.98
613.83
661.76
550.19
272.40
186.36
106.16
164.65
304.40
371.16
463.90
692.07
763.07
827.54
906.21
934.37
Relative Abundance
m/z

Compound **18**

^{1}H NMR (300 MHz, CDCl$_3$)

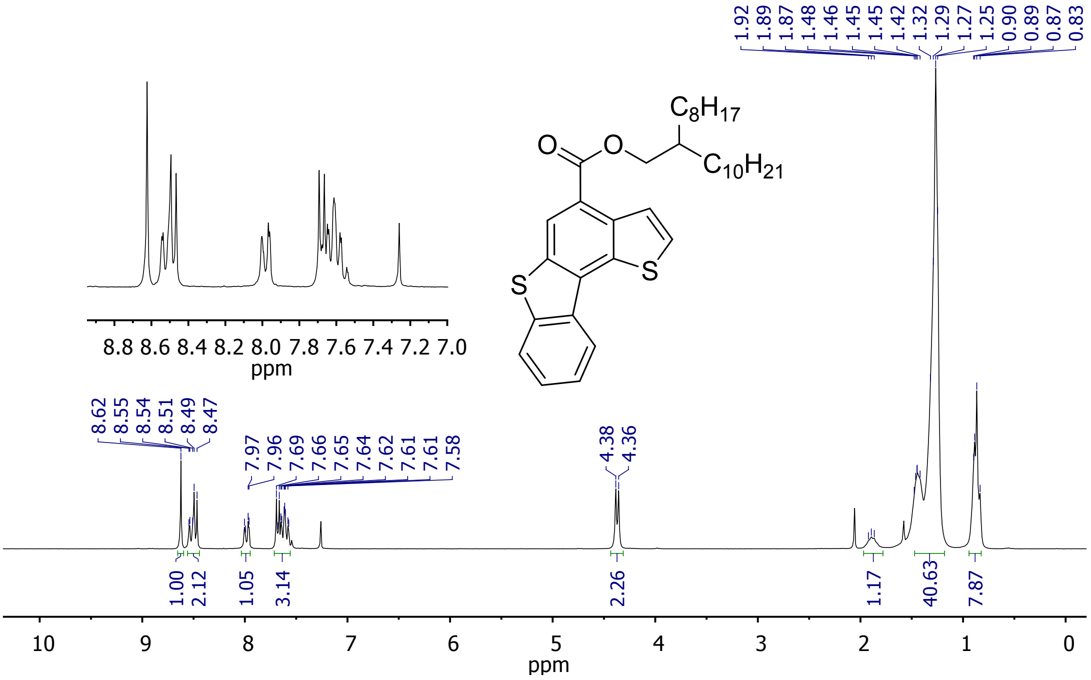

^{13}C NMR (75 MHz, CDCl$_3$)

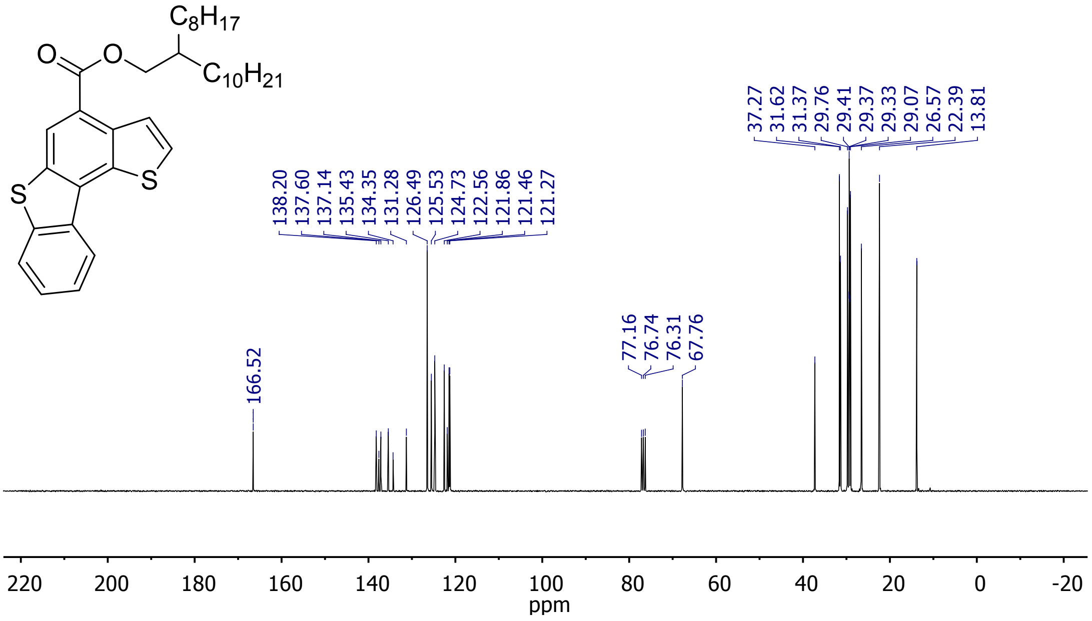

DE-MS

D:\LAVORICGS_2016\UNIPV\Pasini\pasini0   10/10/2017 4:56:56 PM   AN456

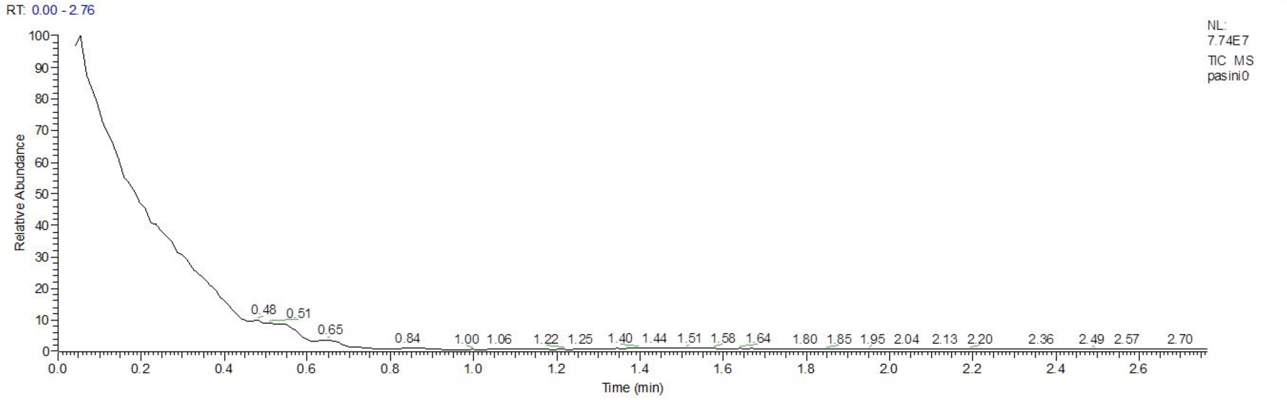

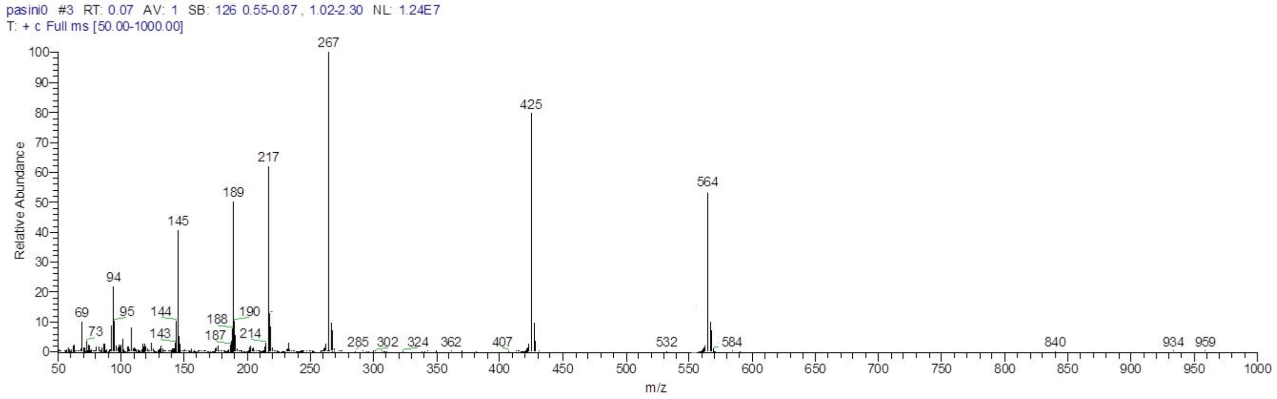

Compound **19**

^{1}H NMR (300 MHz, CDCl$_3$)

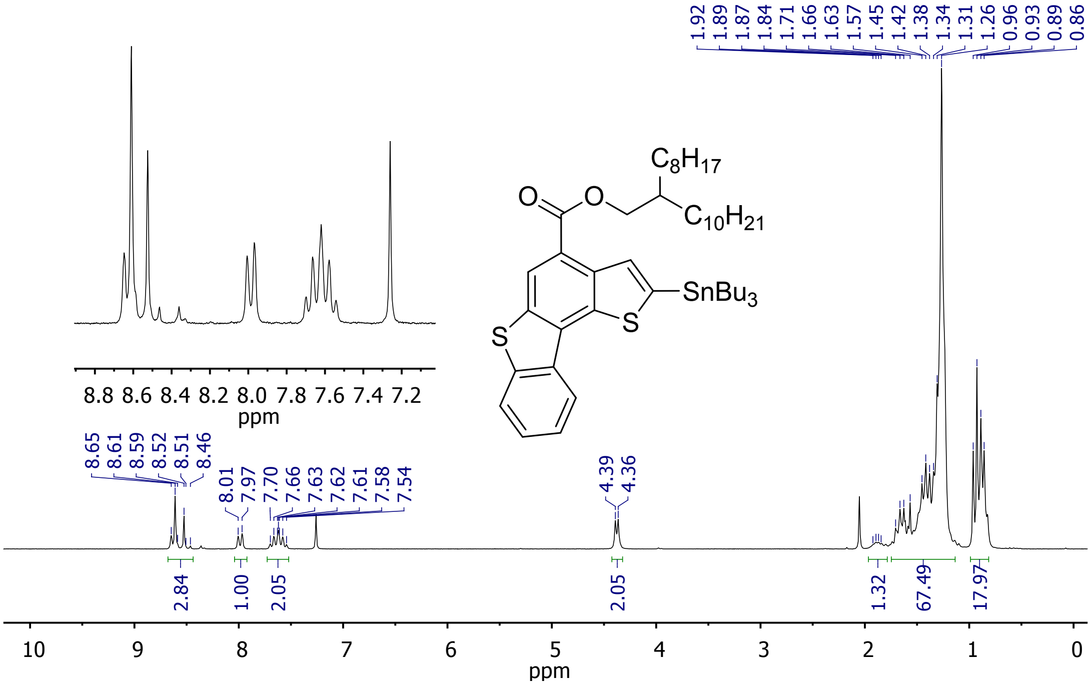

^{13}C NMR (75 MHz, CDCl$_3$)

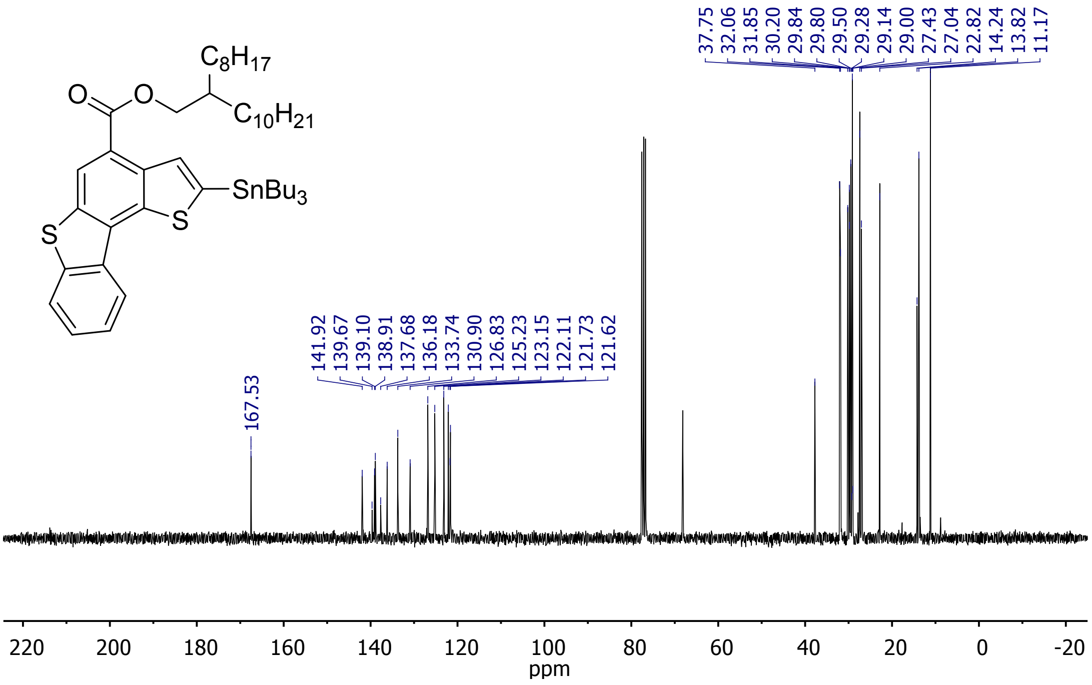

Compound **20**

^{1}H NMR (300 MHz, $CDCl_3$)

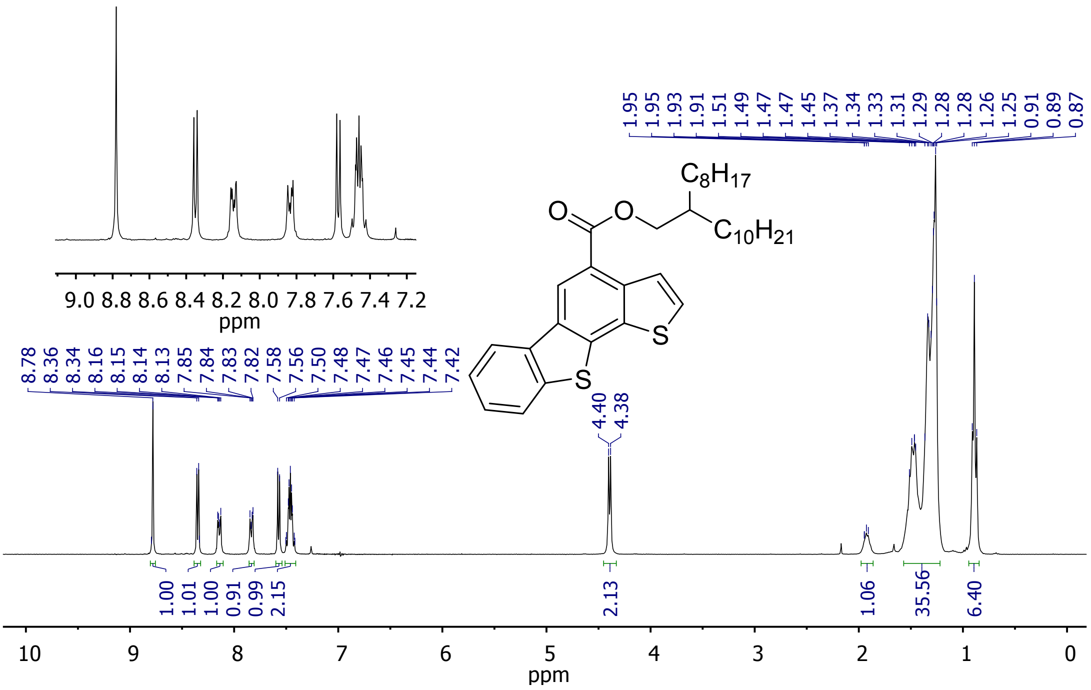

^{13}C NMR (75 MHz, $CDCl_3$)

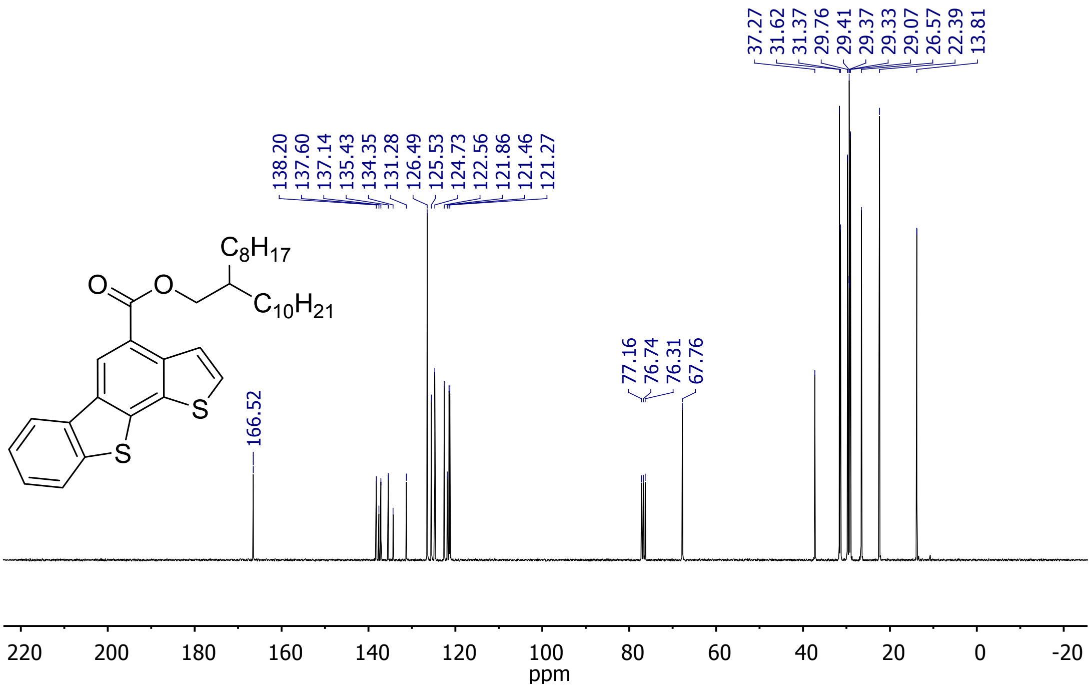

DE-MS

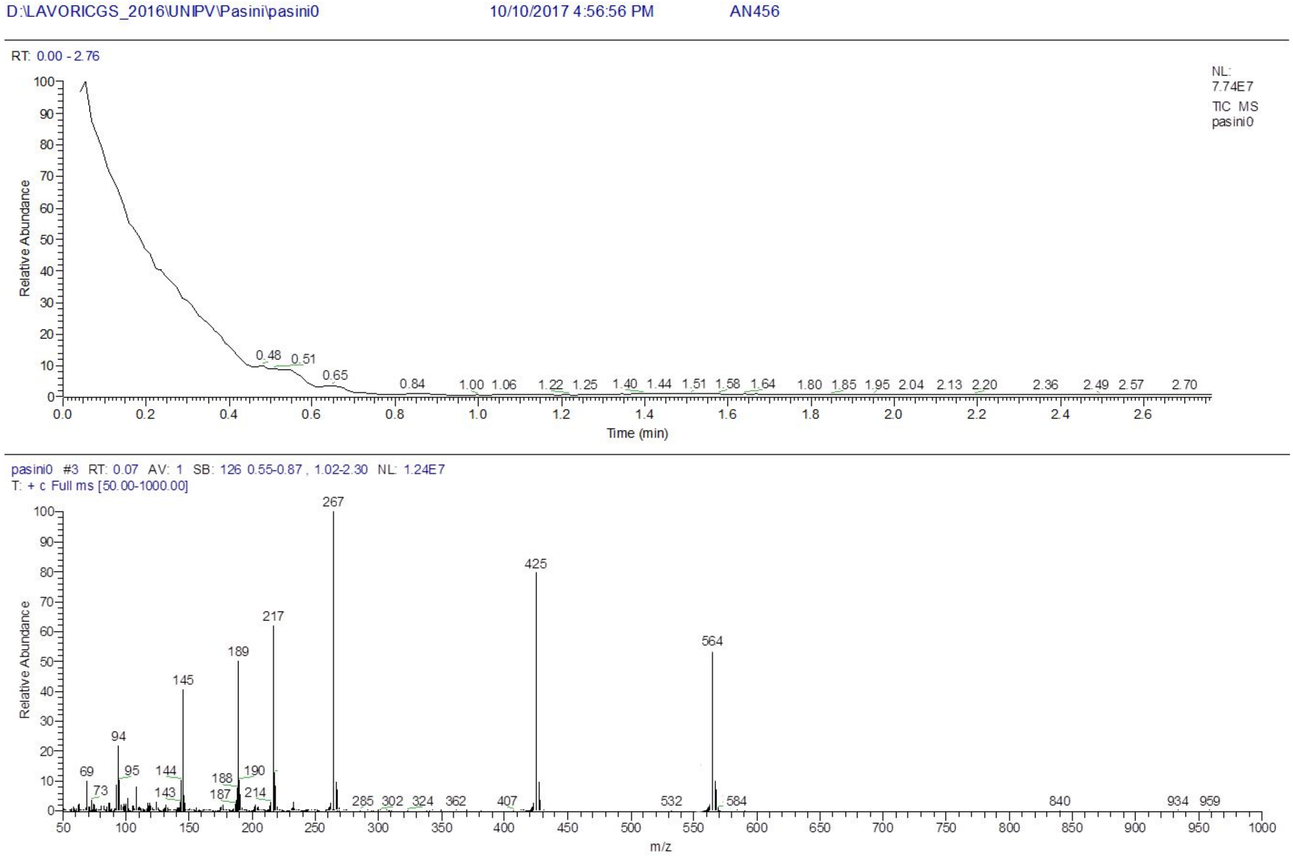

D:\LAVORICGS_2016\UNIPV\Pasini\pasini0
10/10/2017 4:56:56 PM
AN456
RT: 0.00 - 2.76
NL: 7.74E7
TIC MS pasini0
Relative Abundance
Time (min)
0.48
0.51
0.65
0.84
1.00
1.06
1.22
1.25
1.40
1.44
1.51
1.58
1.64
1.80
1.85
1.95
2.04
2.13
2.20
2.36
2.49
2.57
2.70
pasini0 #3 RT: 0.07 AV: 1 SB: 126 0.55-0.87 , 1.02-2.30 NL: 1.24E7
T: + c Full ms [50.00-1000.00]
267
425
217
564
189
145
94
69
95
144
188
190
73
143
187
214
285
302
324
362
407
532
584
840
934
959
m/z

Compound **21**

^{1}H NMR (300 MHz, $CDCl_3$)

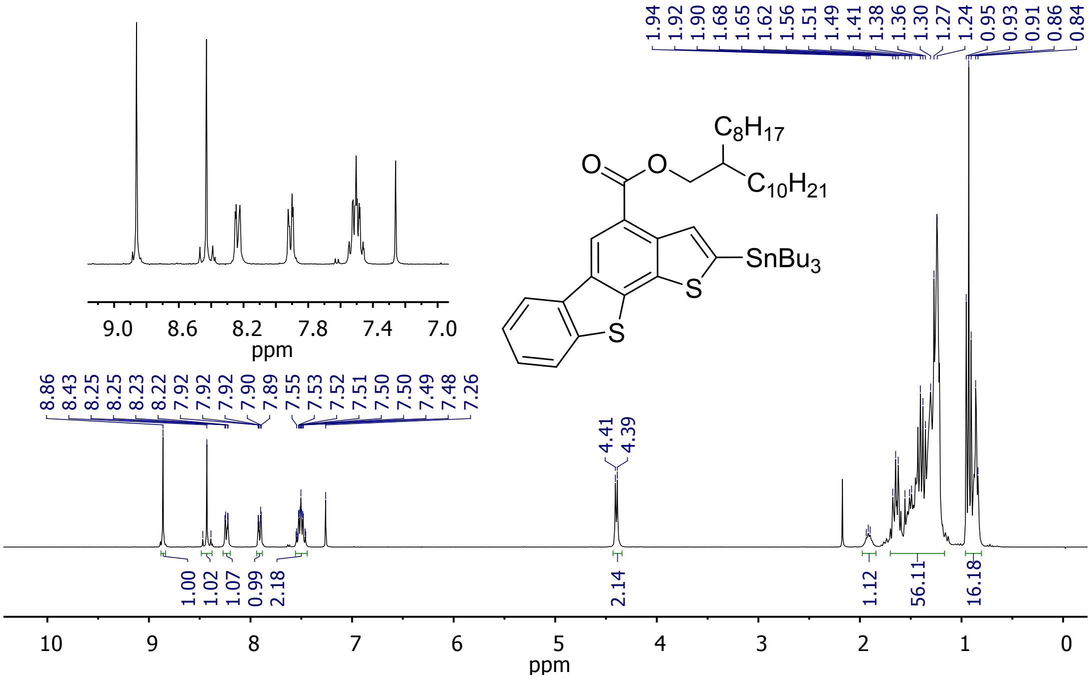

^{13}C NMR (75 MHz, $CDCl_3$)

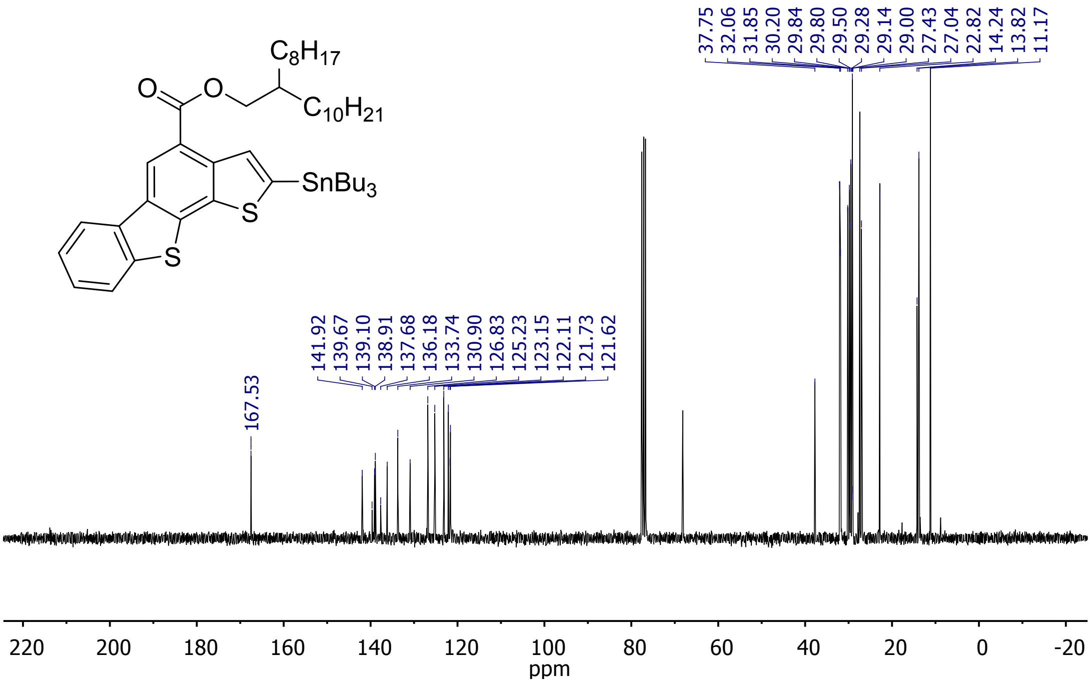

Compound **24**

^{1}H NMR (400 MHz, $CDCl_3$)

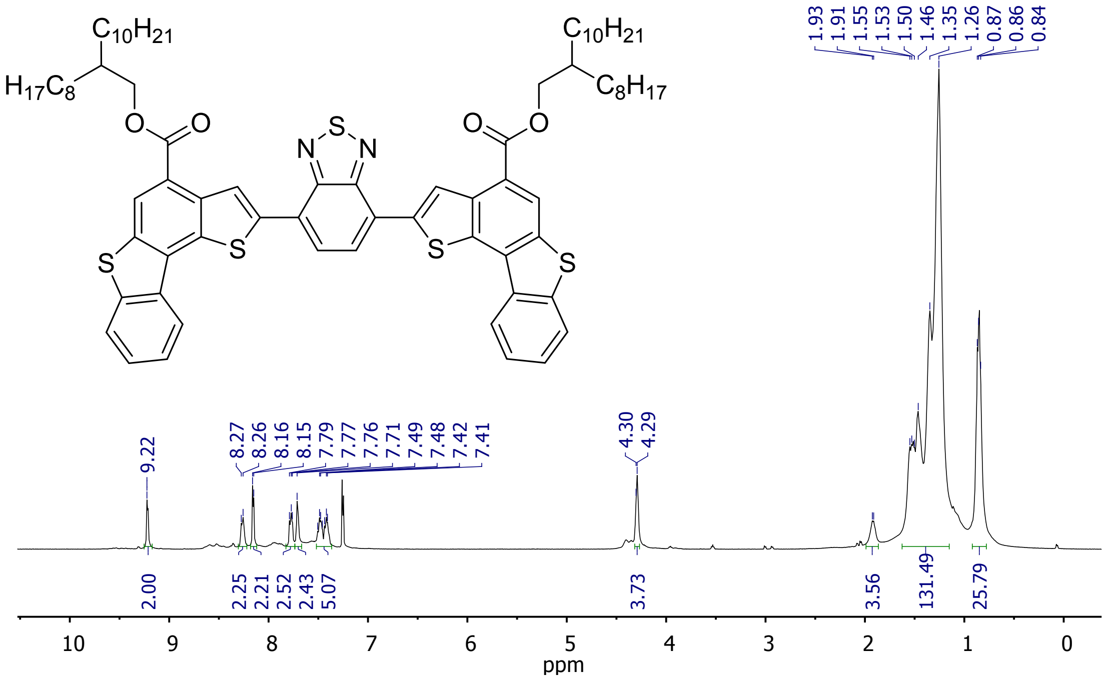

^{13}C NMR (101 MHz, $CDCl_3$)

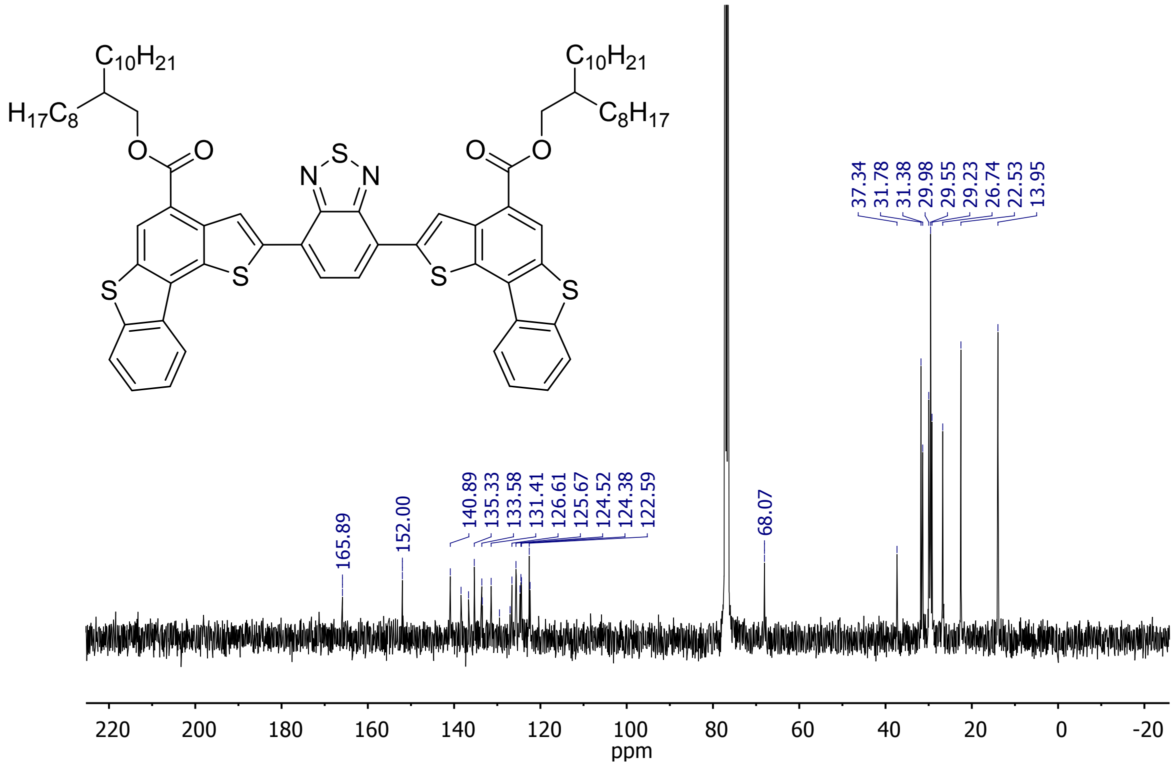

DEPT

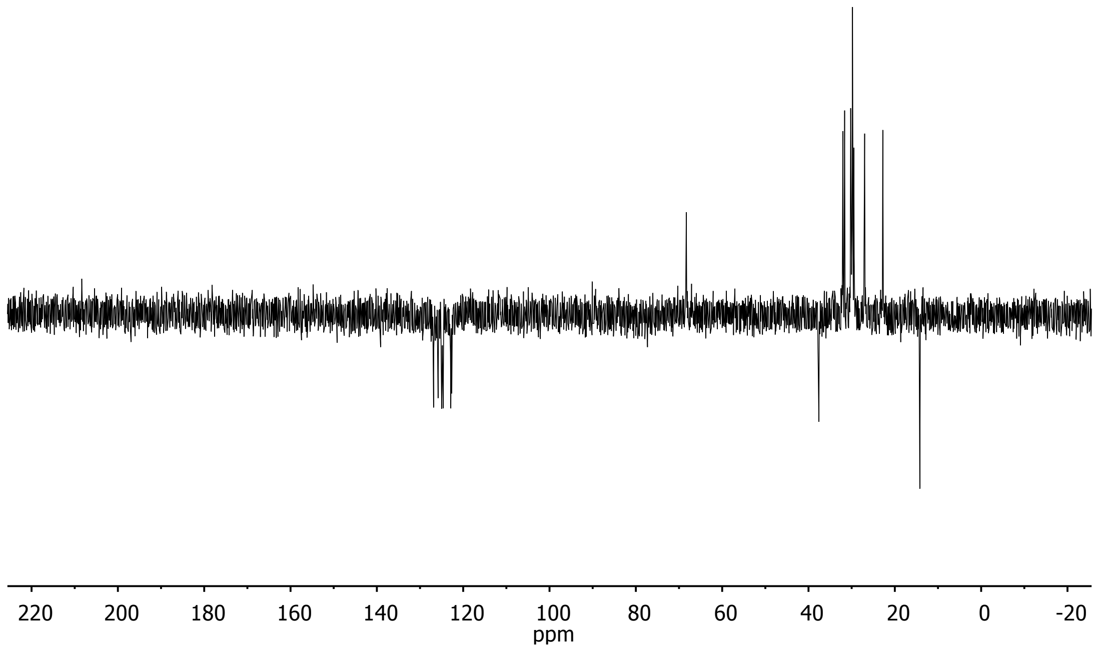
220
200
180
160
140
120
100
ppm
80
60
40
20
0
-20

HMQC

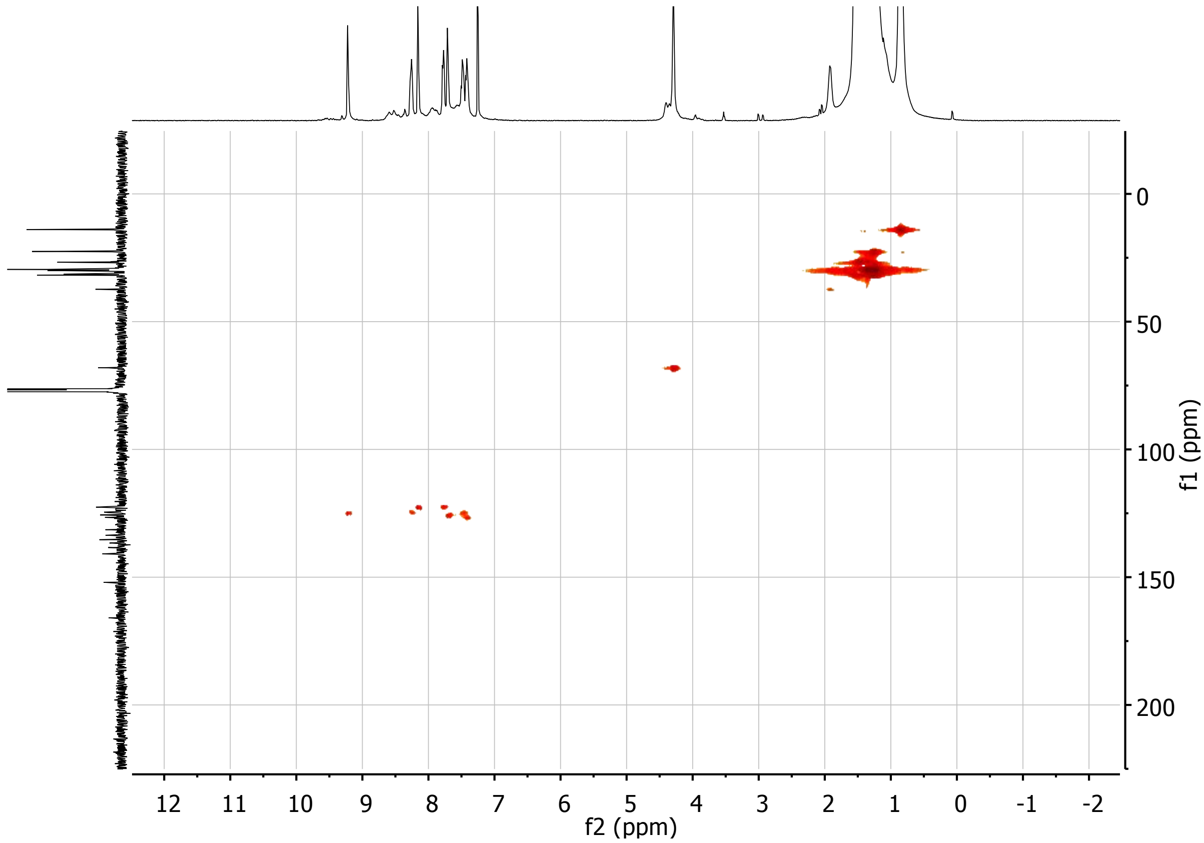
12
11
10
9
8
7
6
5
4
3
2
1
0
-1
-2
f2 (ppm)
0
50
100
150
200
f1 (ppm)

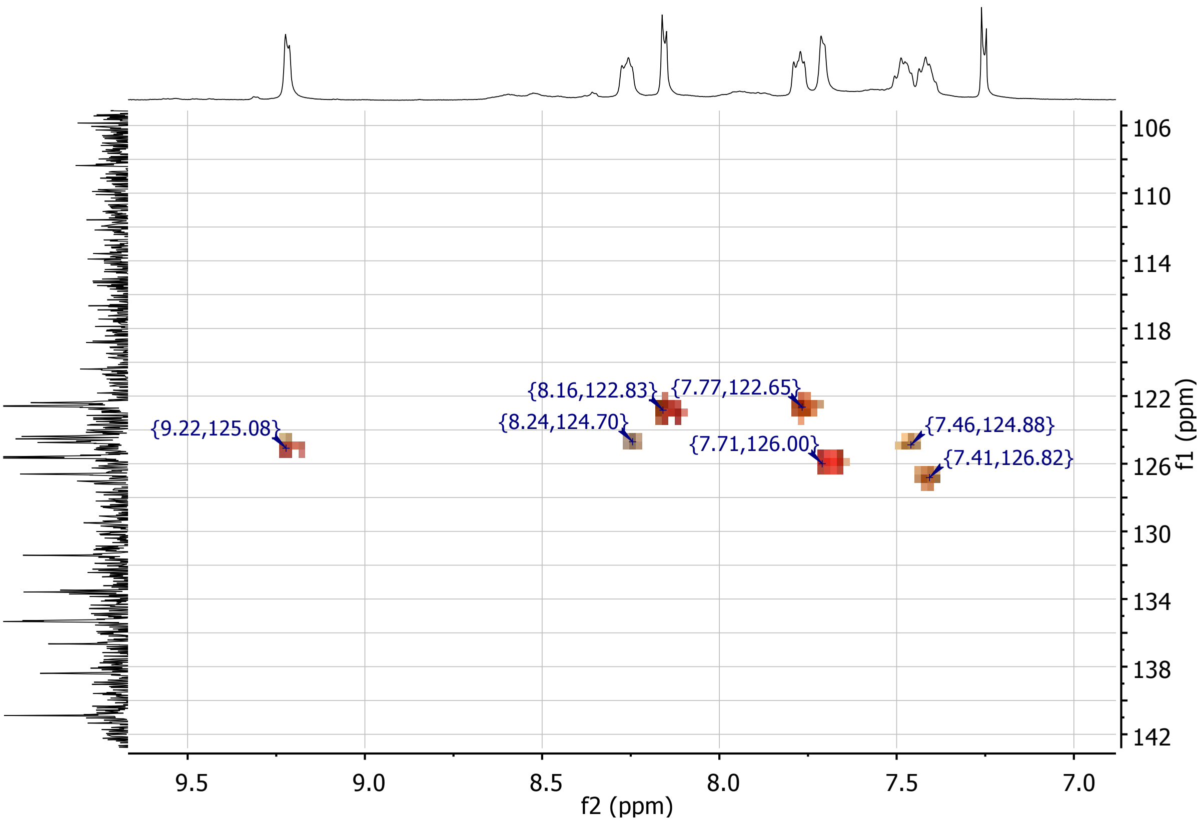

{9.22,125.08}
{8.24,124.70}
{8.16,122.83}
{7.77,122.65}
{7.71,126.00}
{7.46,124.88}
{7.41,126.82}
f2 (ppm)
f1 (ppm)

HMBC

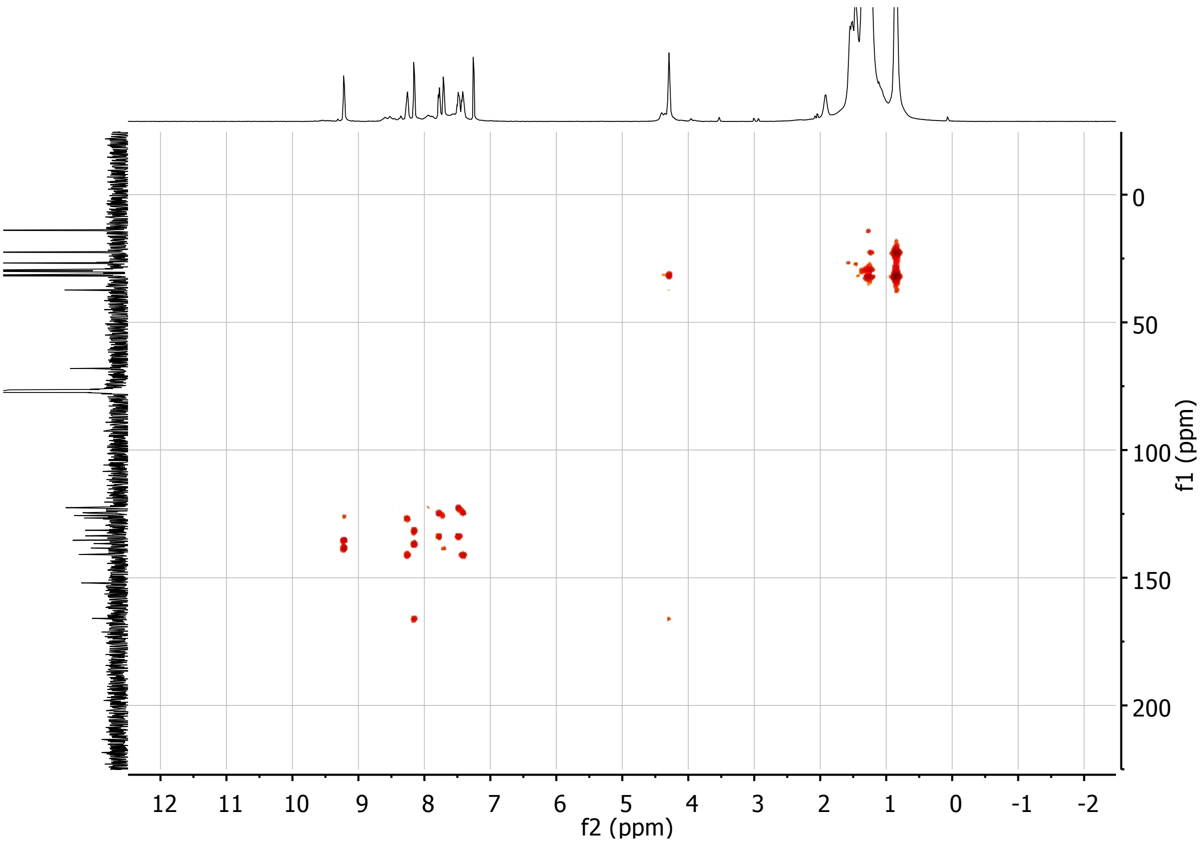

f2 (ppm)
f1 (ppm)

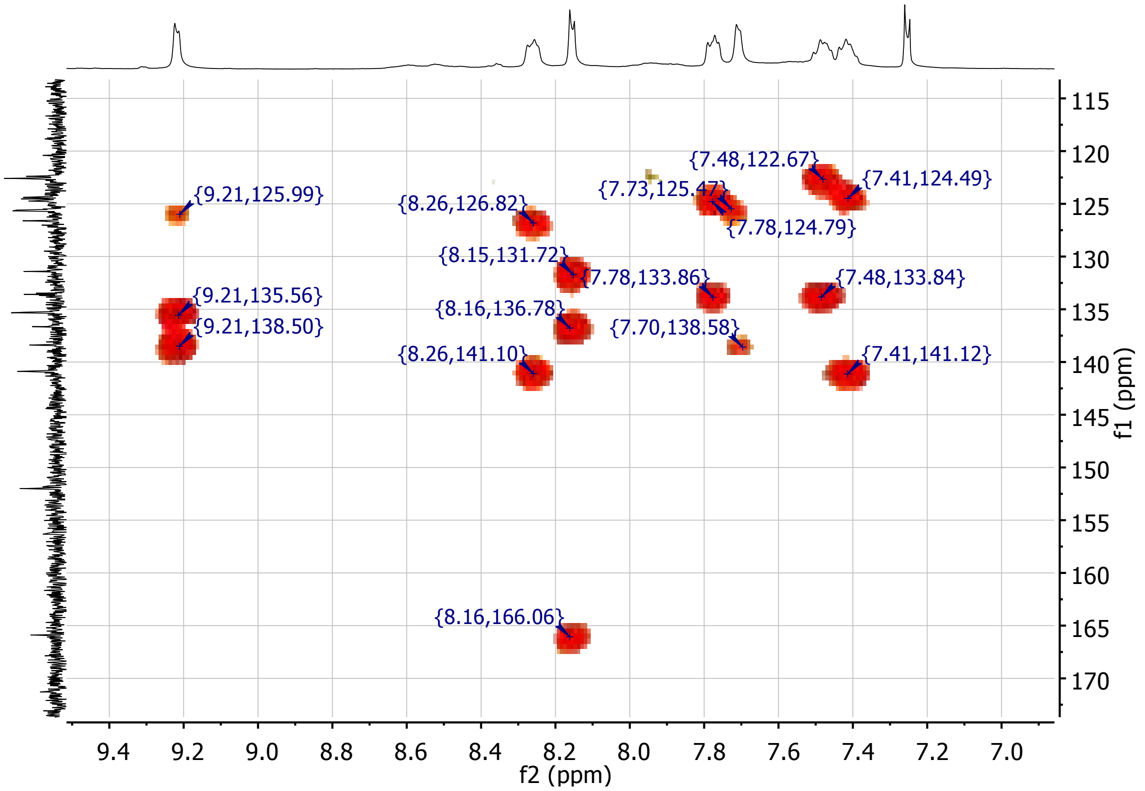
{9.21,125.99}
{8.26,126.82}
{7.73,125.47}
{7.48,122.67}
{7.41,124.49}
{7.78,124.79}
{8.15,131.72}
{7.78,133.86}
{7.48,133.84}
{9.21,135.56}
{9.21,138.50}
{8.16,136.78}
{7.70,138.58}
{8.26,141.10}
{7.41,141.12}
{8.16,166.06}
f1 (ppm)
f2 (ppm)

HRMS

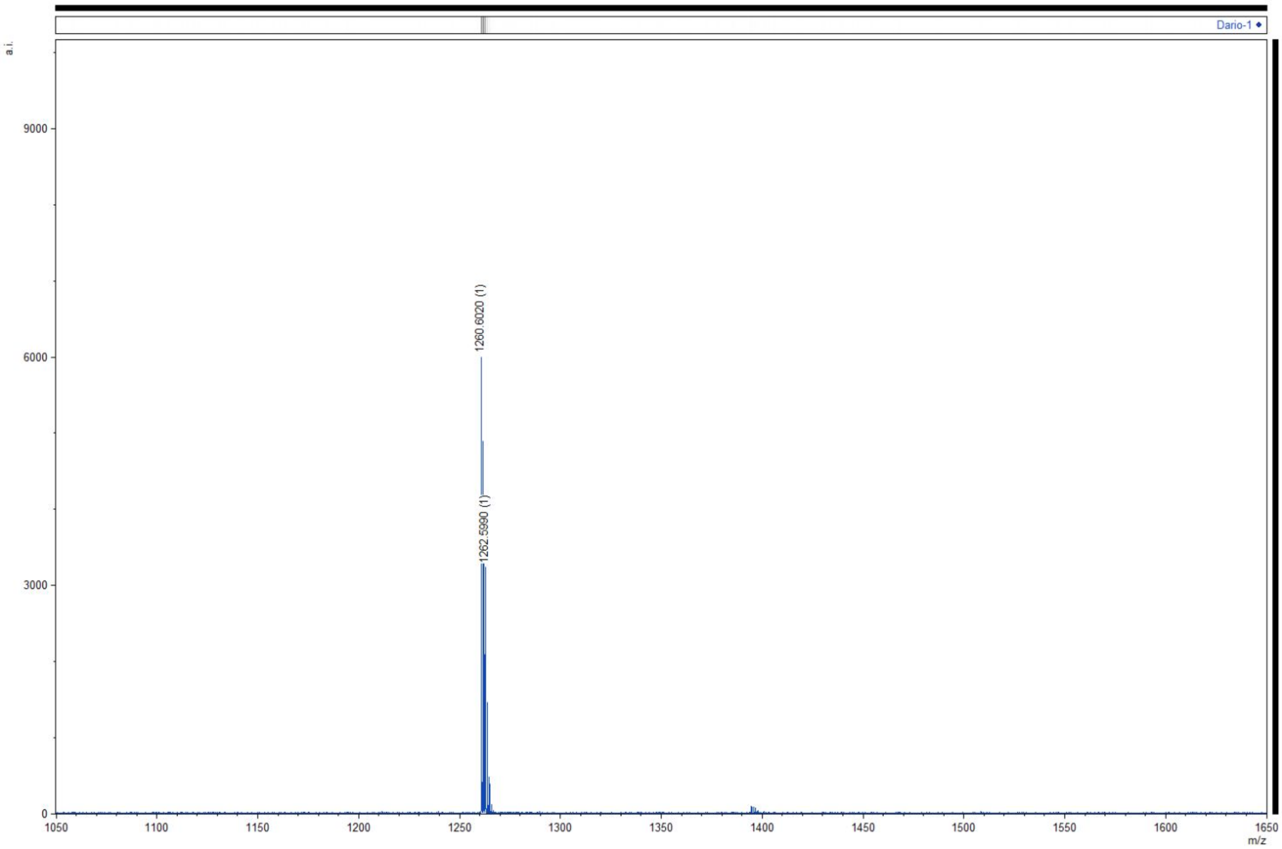
Dario-1
a.i.
1260.6020 (1)
1262.5990 (1)
m/z

Compound **25**

^{1}H NMR (400 MHz, $CDCl_3$)

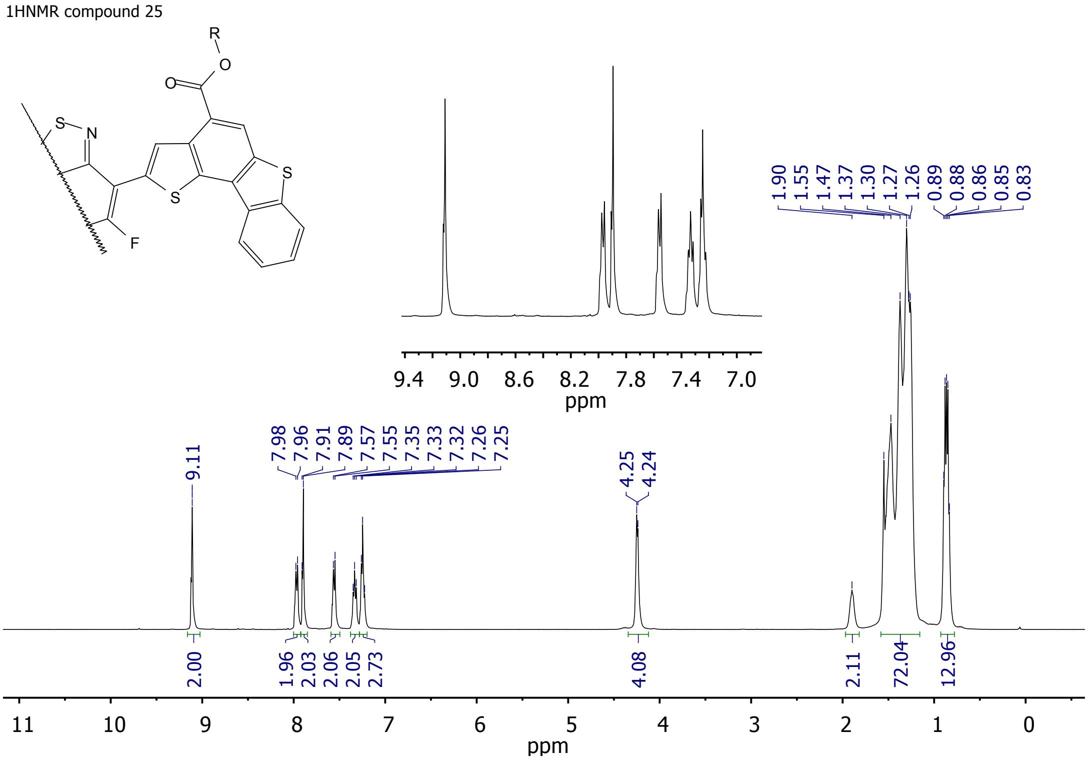

^{19}F NMR

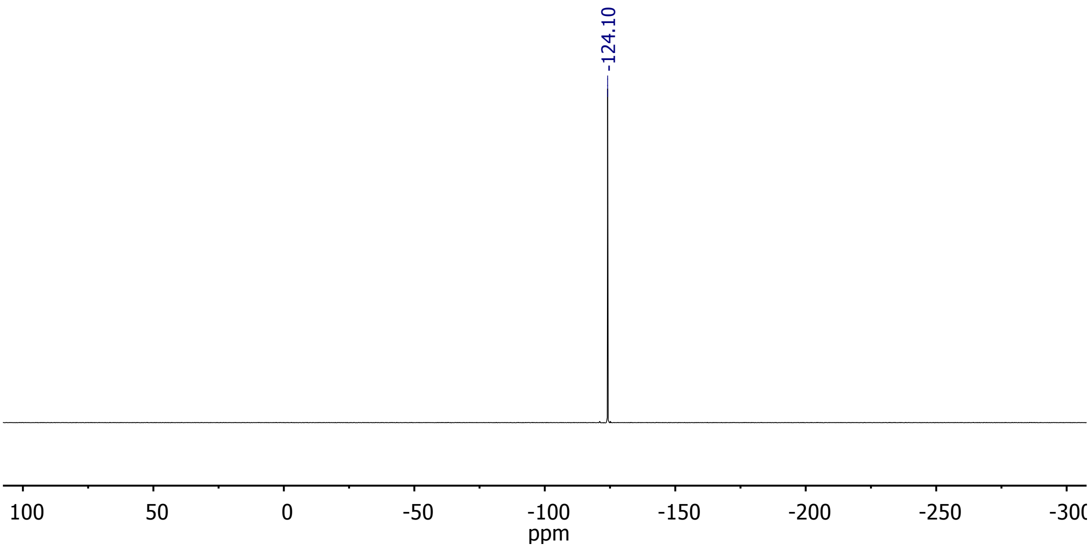

^{13}C NMR (101 MHz, $CDCl_3$)

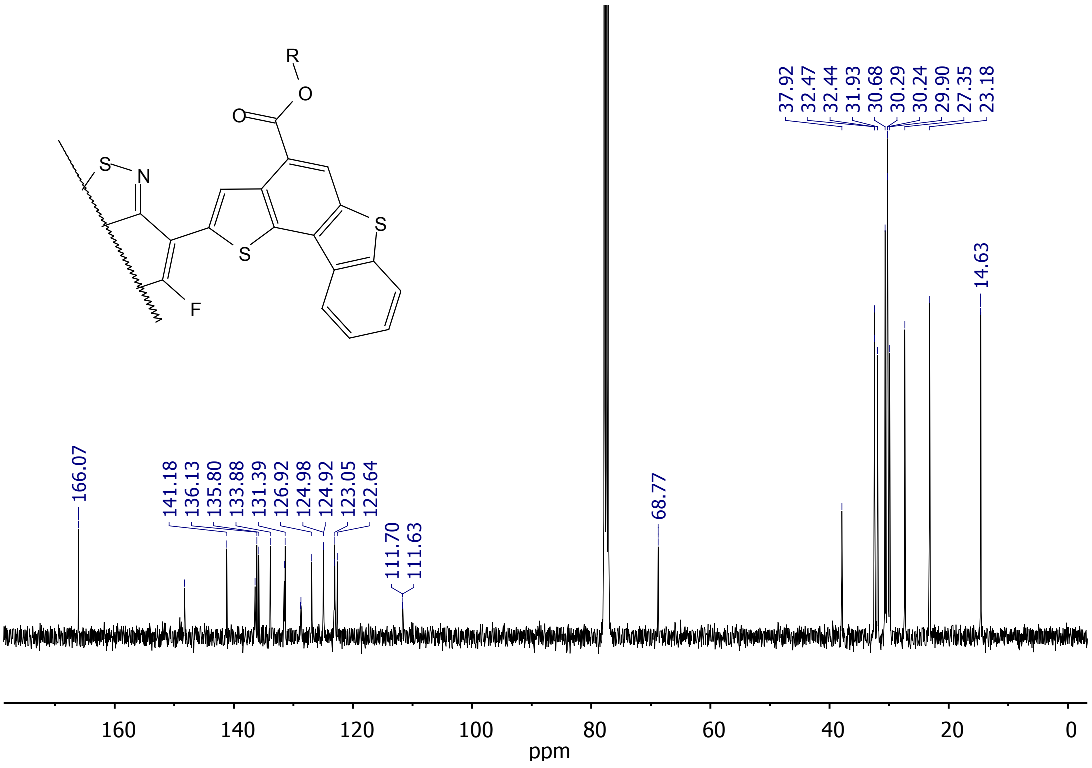

DEPT

DEPT compound 25

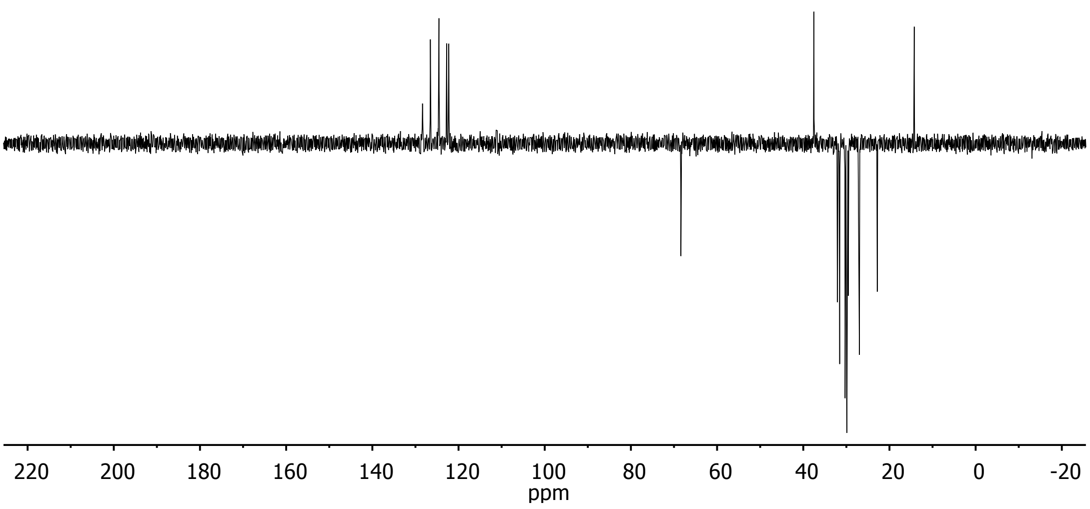

HMQC

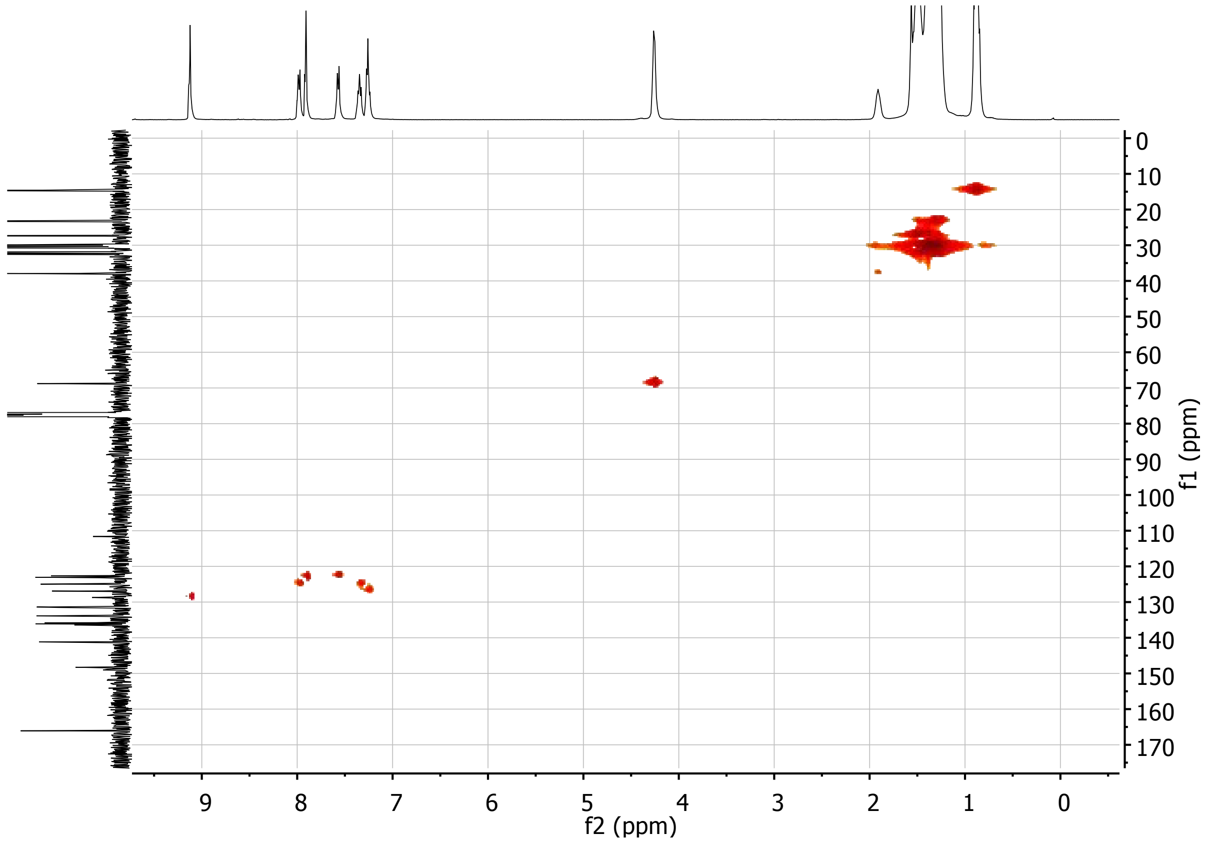

f2 (ppm)
f1 (ppm)

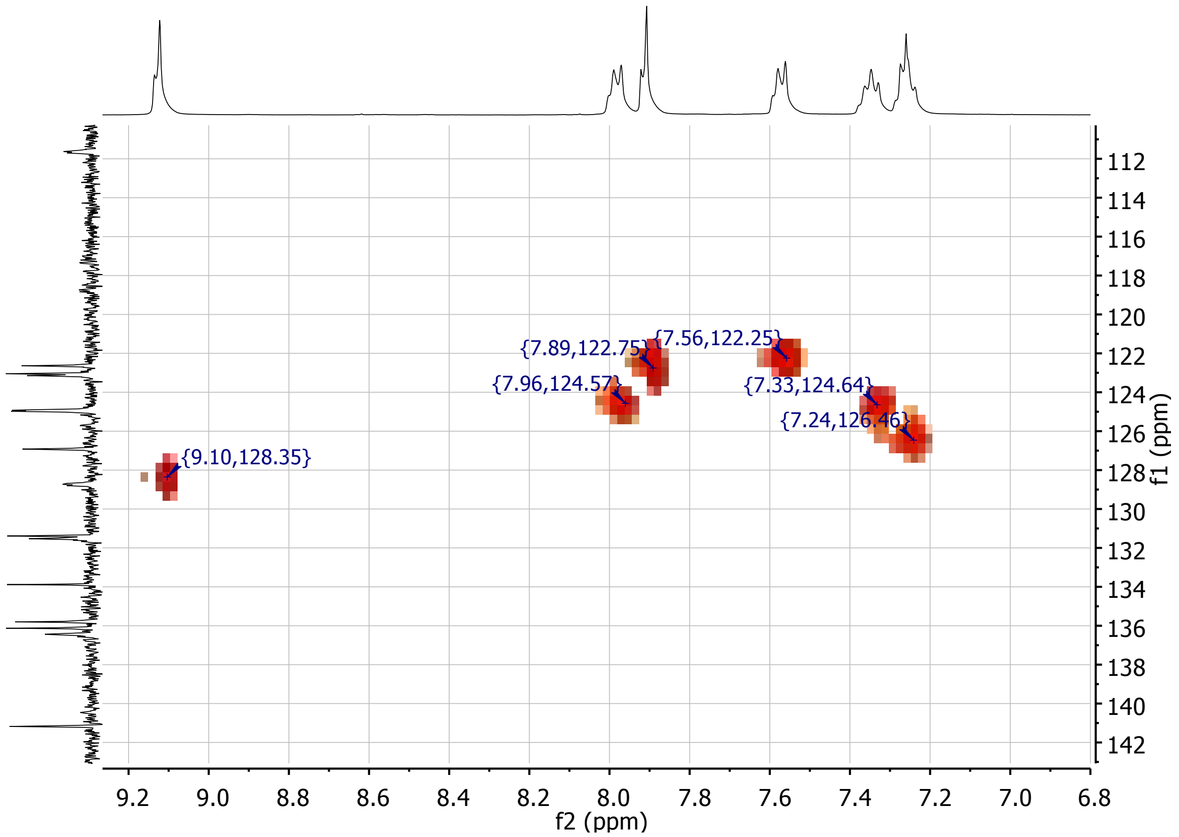

{7.89,122.75}
{7.56,122.25}
{7.96,124.57}
{7.33,124.64}
{7.24,126.46}
{9.10,128.35}
f2 (ppm)
f1 (ppm)

HMBC

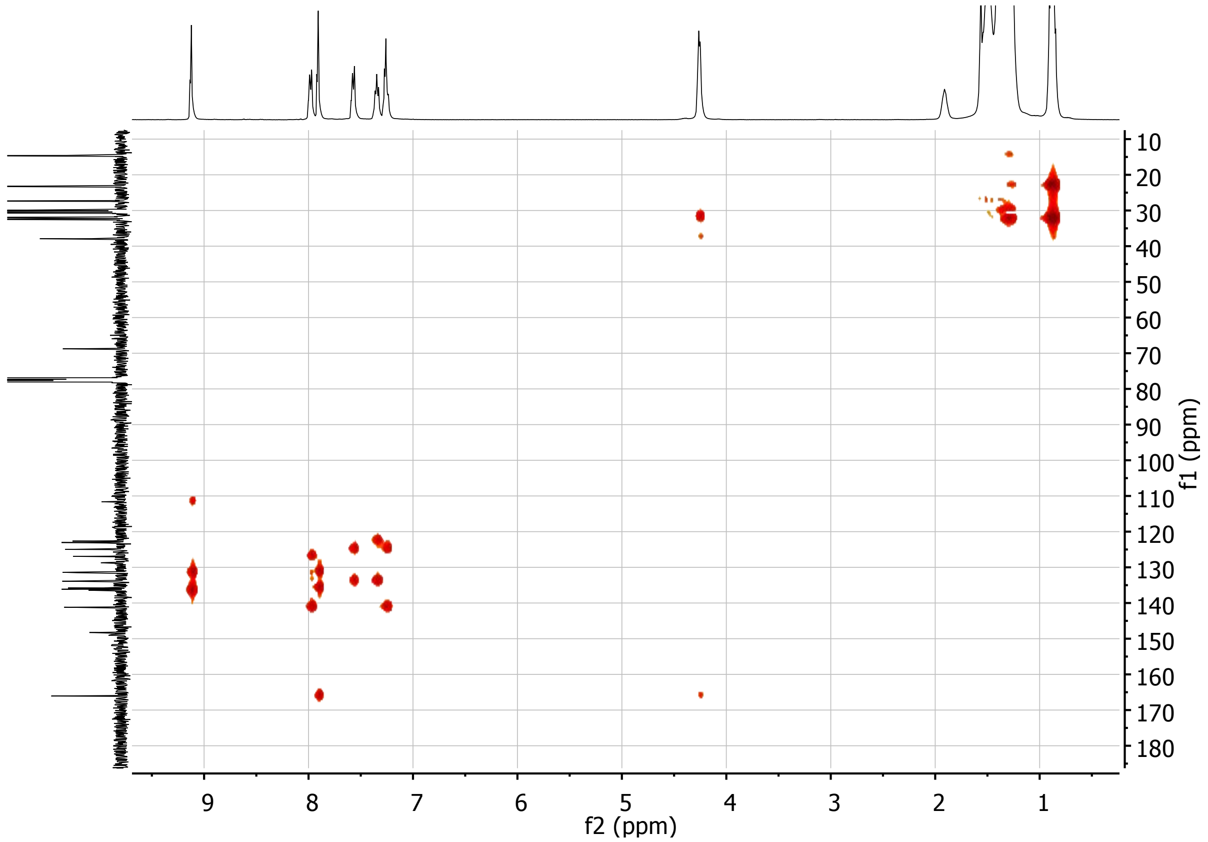

f2 (ppm)
f1 (ppm)

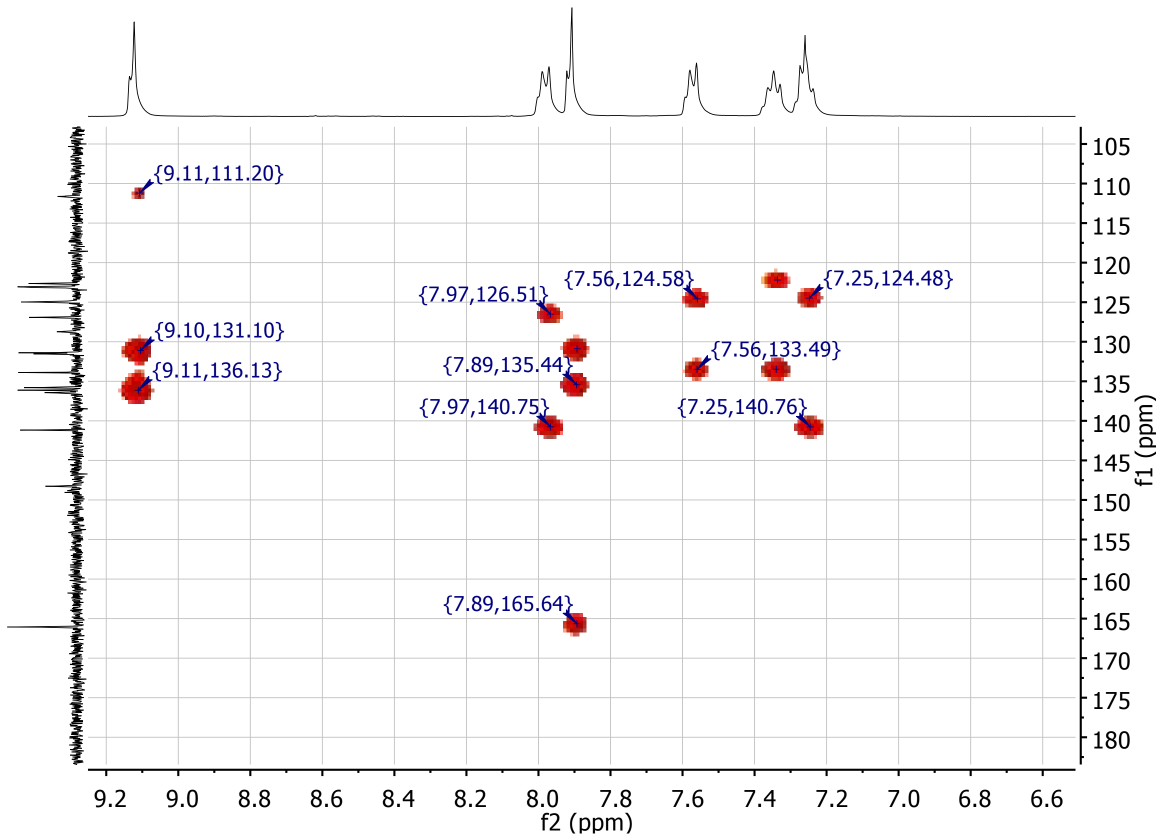

{9.11,111.20}
{7.56,124.58}
{7.25,124.48}
{7.97,126.51}
{9.10,131.10}
{7.56,133.49}
{9.11,136.13}
{7.89,135.44}
{7.97,140.75}
{7.25,140.76}
{7.89,165.64}
f2 (ppm)
f1 (ppm)

HRMS

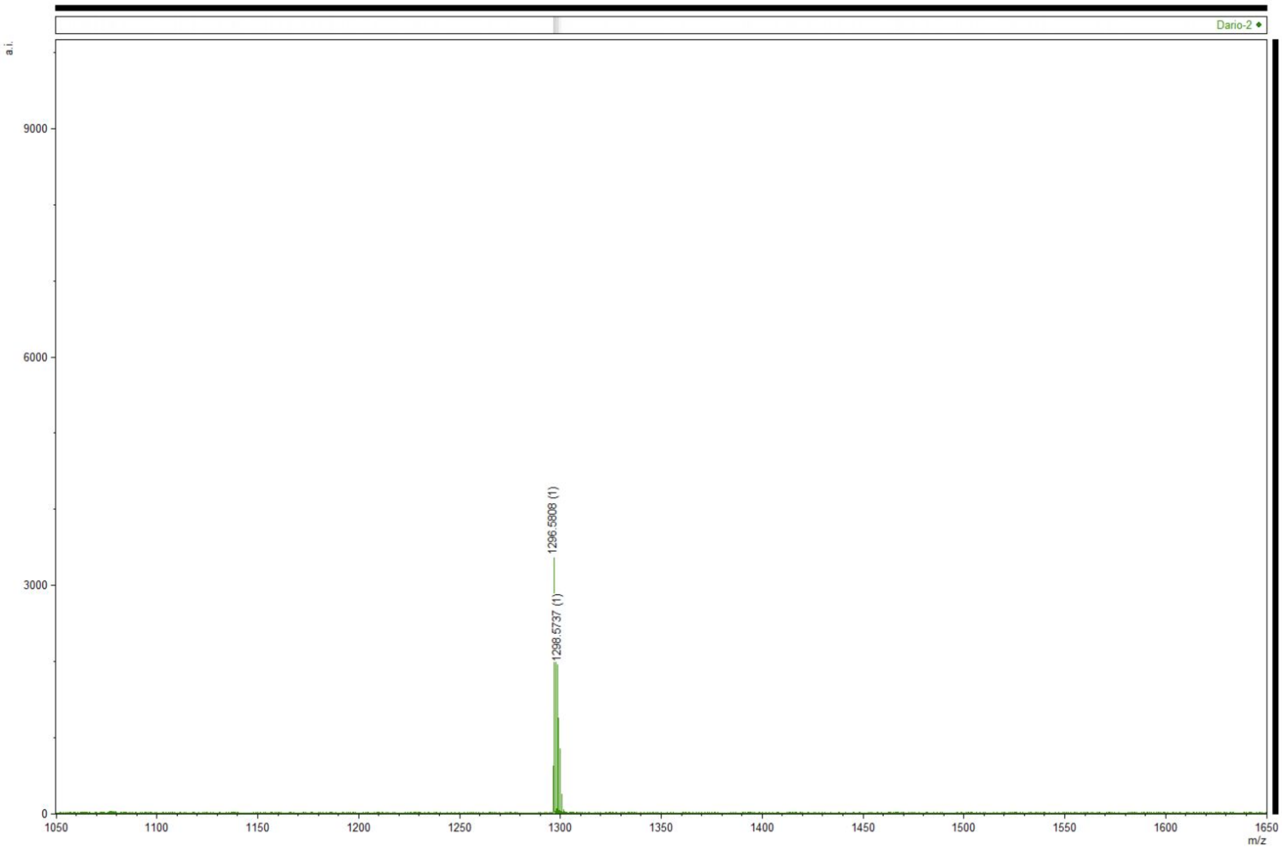

Dario-2
a.i.
9000
6000
3000
0
1296.5808 (1)
1298.5737 (1)
1050
1100
1150
1200
1250
1300
1350
1400
1450
1500
1550
1600
1650
m/z

Compound **26**

^1H NMR (400 MHz, $CDCl_3$)

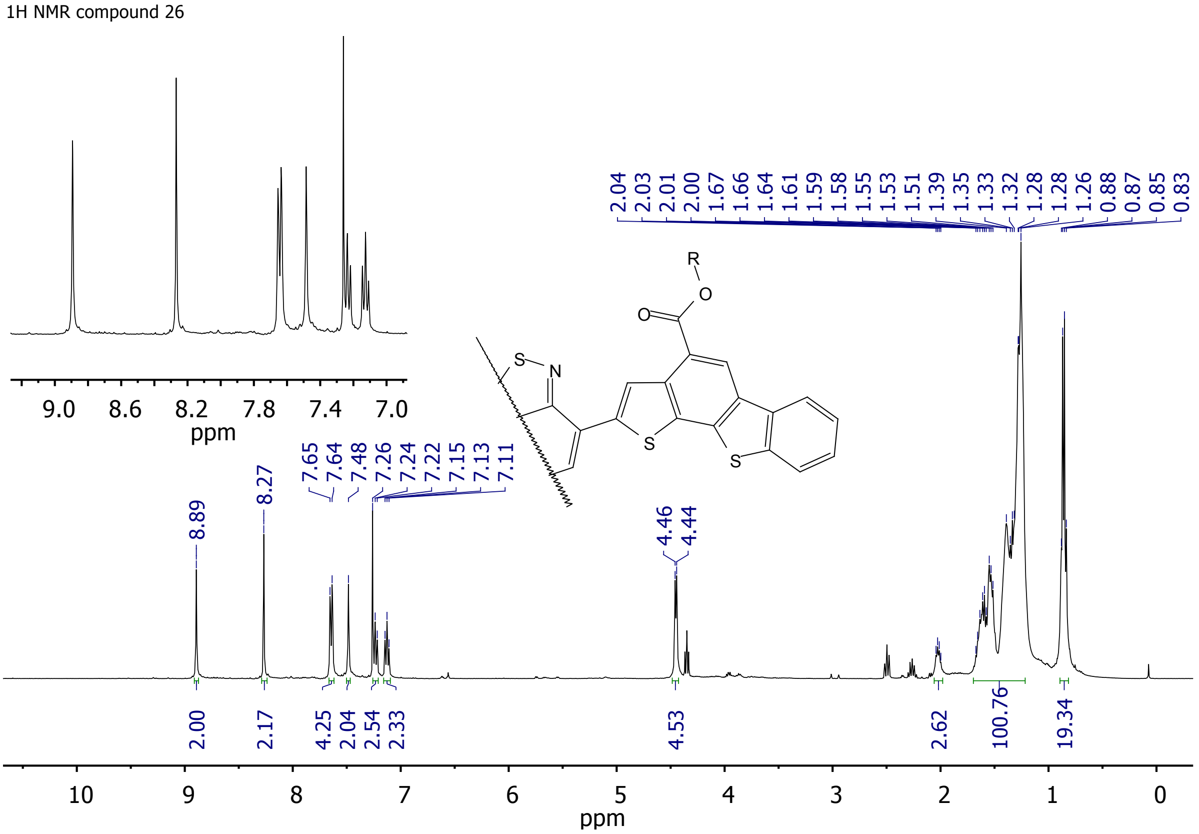

^{13}C NMR (101 MHz, $CDCl_3$)

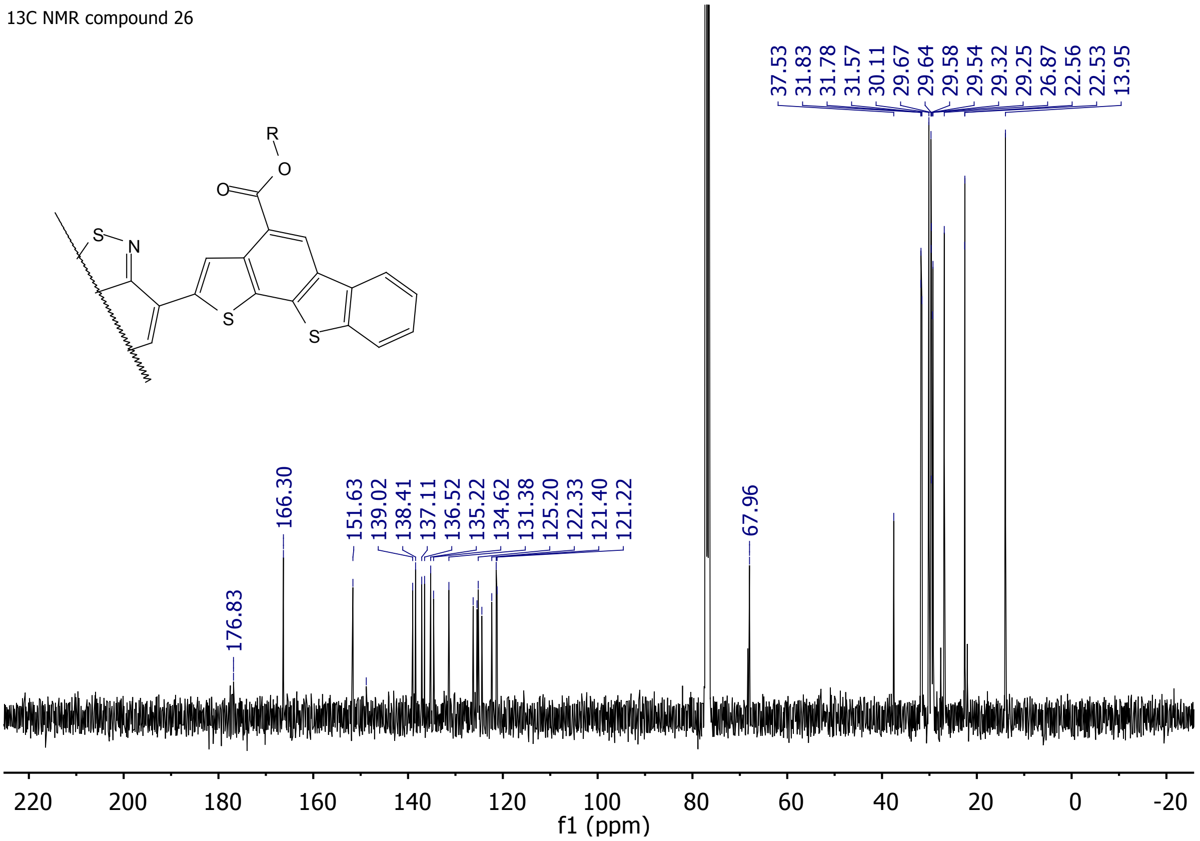

DEPT

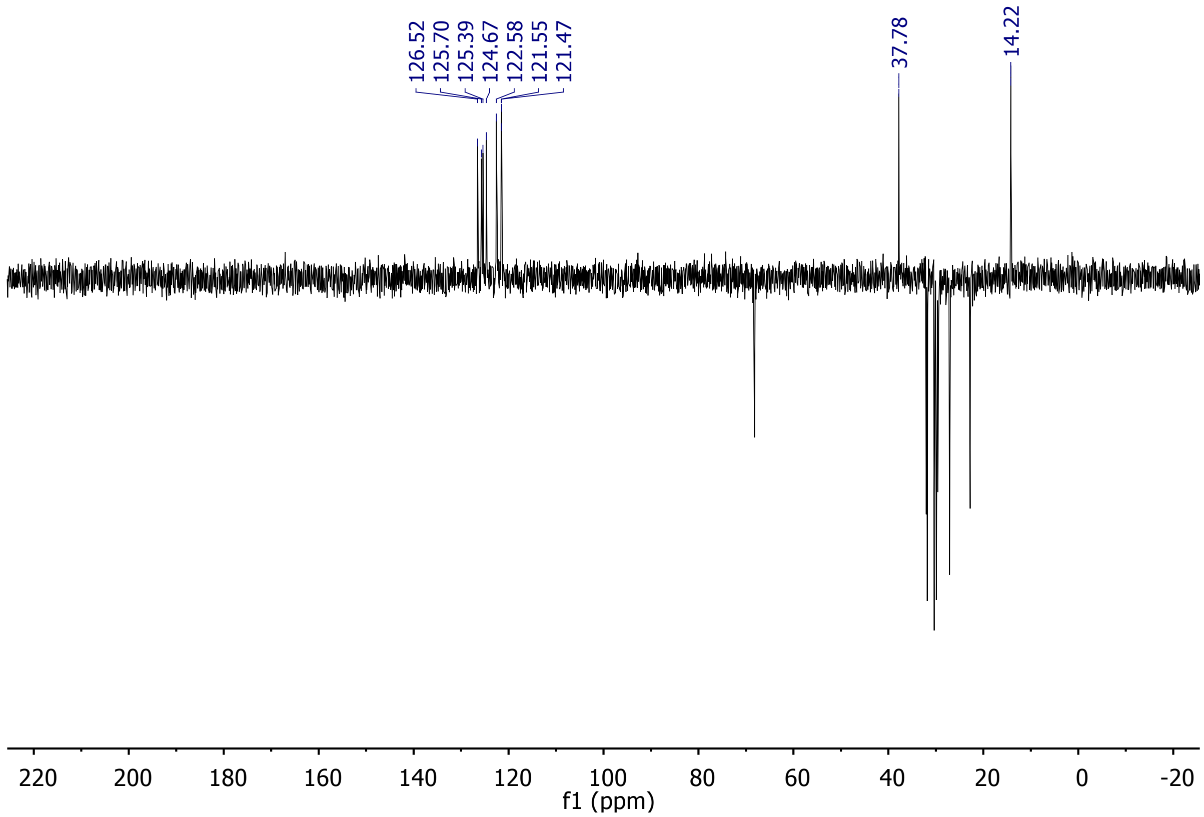

126.52
125.70
125.39
124.67
122.58
121.55
121.47
37.78
14.22
220
200
180
160
140
120
100
80
60
40
20
0
-20
f1 (ppm)

HMQC

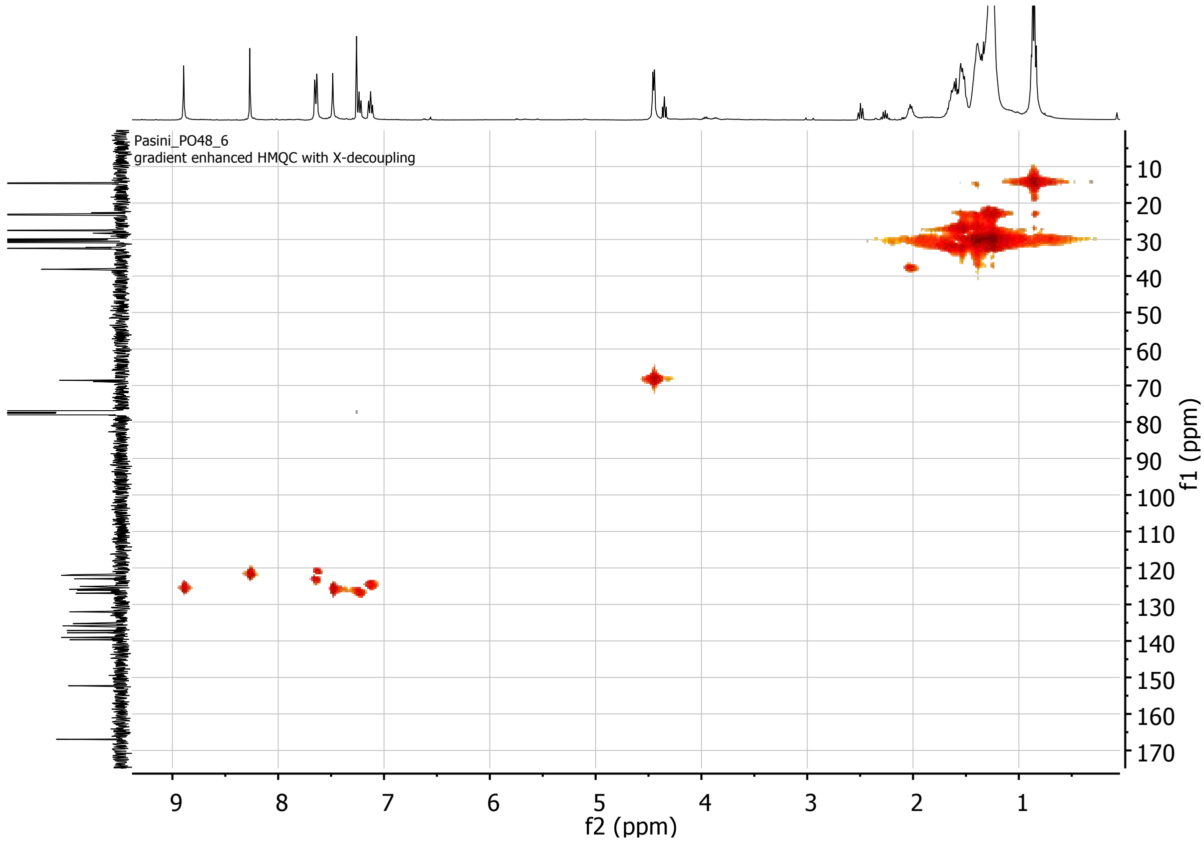

Pasini_PO48_6
gradient enhanced HMQC with X-decoupling
f1 (ppm)
f2 (ppm)

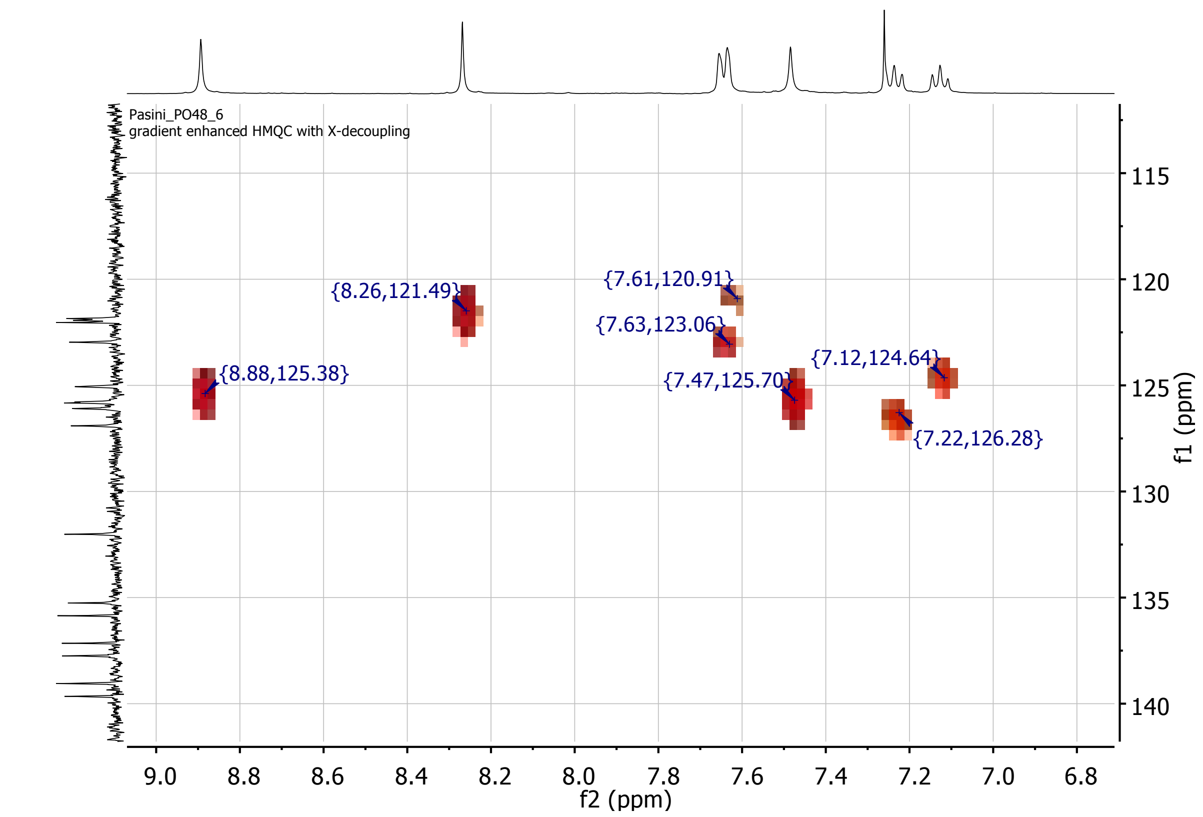
Pasini_PO48_6
gradient enhanced HMQC with X-decoupling
{8.26,121.49}
{7.61,120.91}
{7.63,123.06}
{8.88,125.38}
{7.12,124.64}
{7.47,125.70}
{7.22,126.28}
f2 (ppm)
f1 (ppm)

HMBC

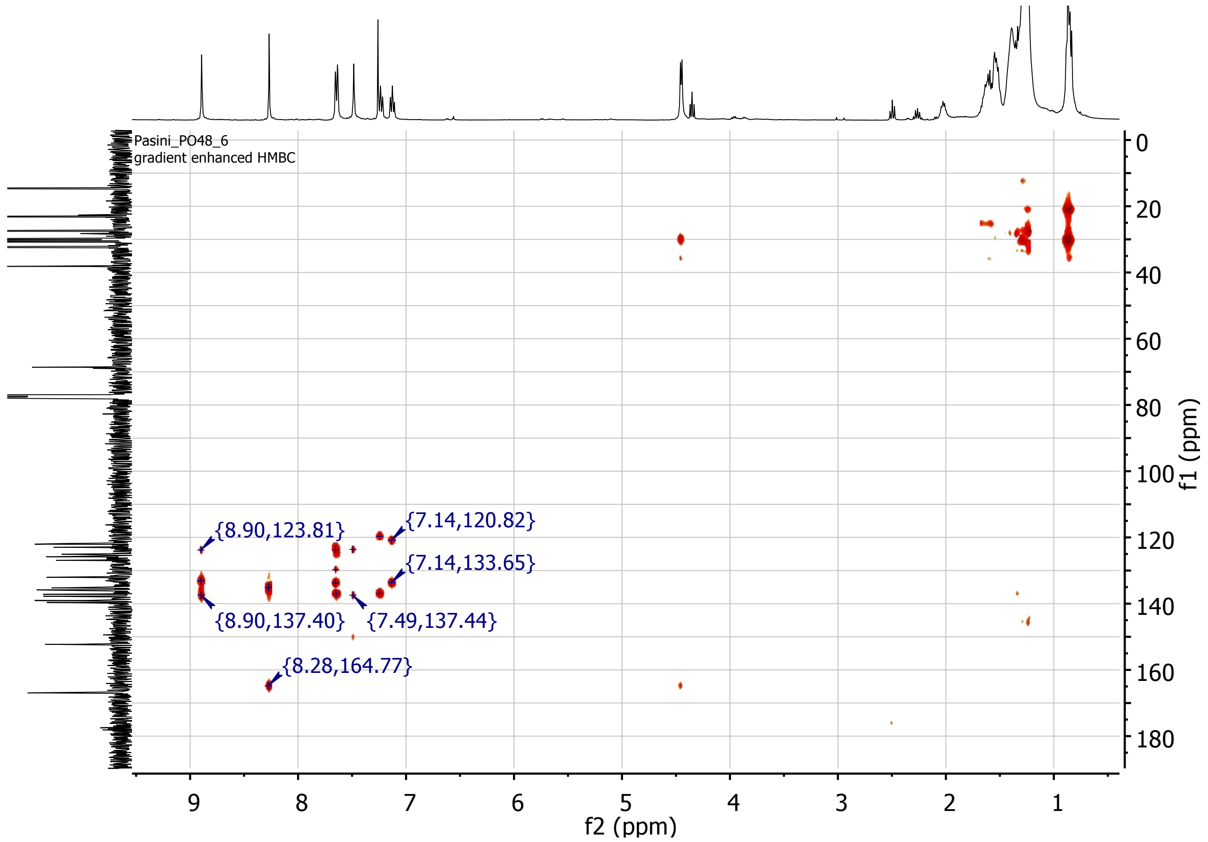
Pasini_PO48_6
gradient enhanced HMBC
{8.90,123.81}
{7.14,120.82}
{7.14,133.65}
{8.90,137.40}
{7.49,137.44}
{8.28,164.77}
f2 (ppm)
f1 (ppm)

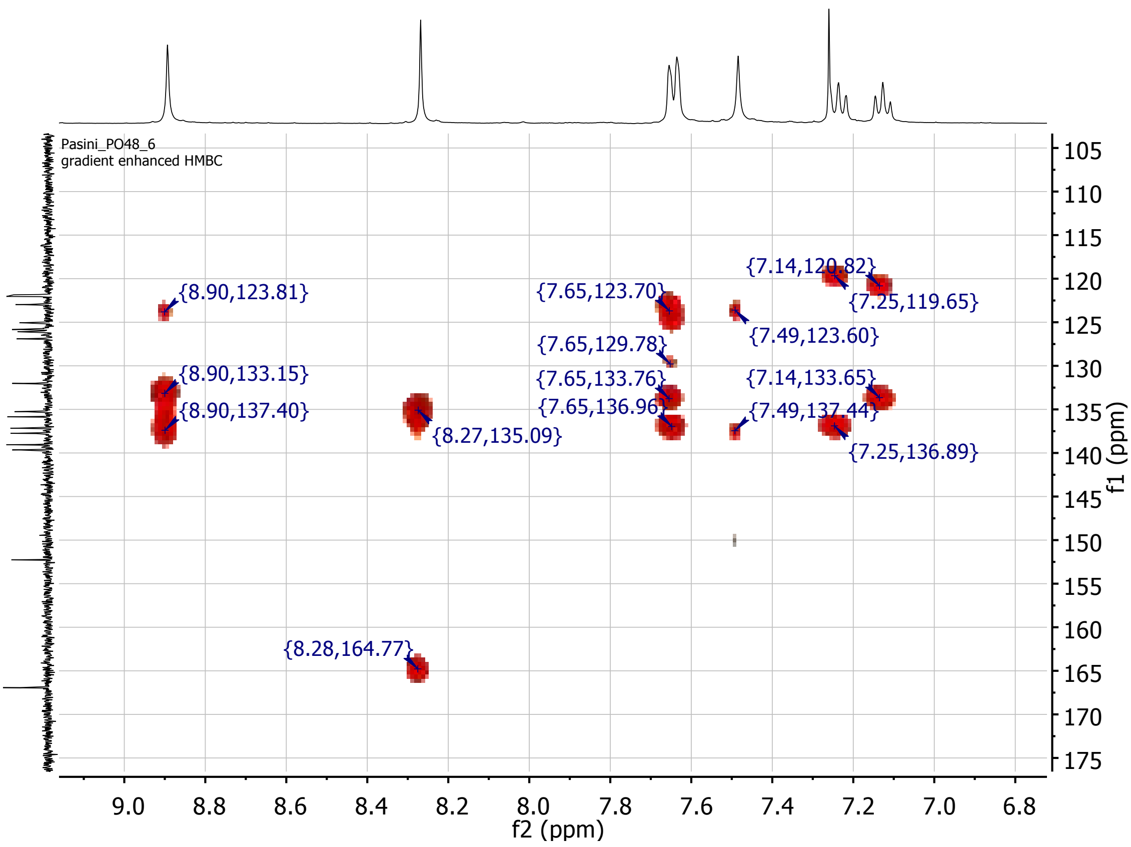
Pasini_PO48_6
gradient enhanced HMBC
{8.90,123.81}
{8.90,133.15}
{8.90,137.40}
{8.27,135.09}
{8.28,164.77}
{7.65,123.70}
{7.65,129.78}
{7.65,133.76}
{7.65,136.96}
{7.49,123.60}
{7.49,137.44}
{7.14,120.82}
{7.25,119.65}
{7.14,133.65}
{7.25,136.89}
f2 (ppm)
f1 (ppm)

HRMS

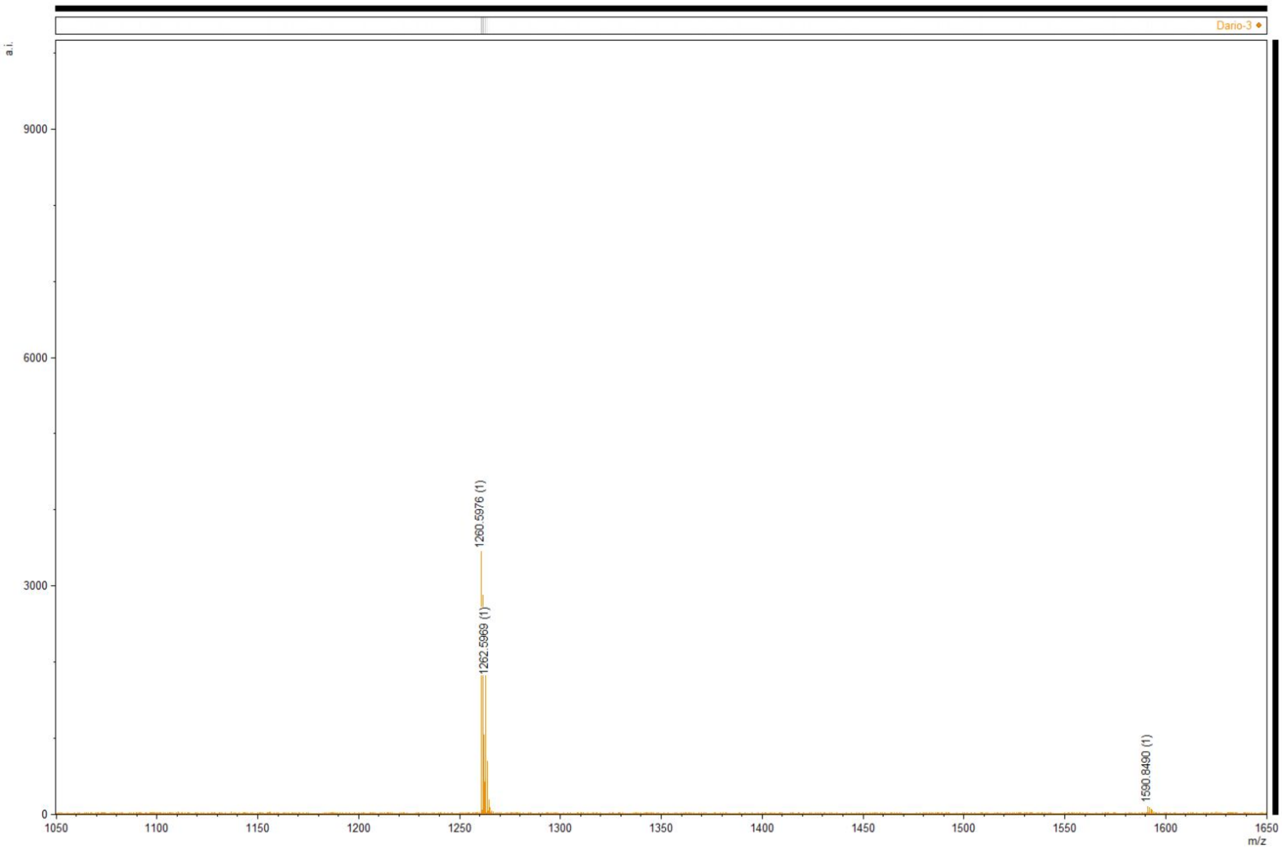
Dario-3
a.i.
1260.5976 (1)
1262.5969 (1)
1590.8490 (1)
m/z

Compound **27**

¹H NMR (400 MHz, $CDCl_3$)

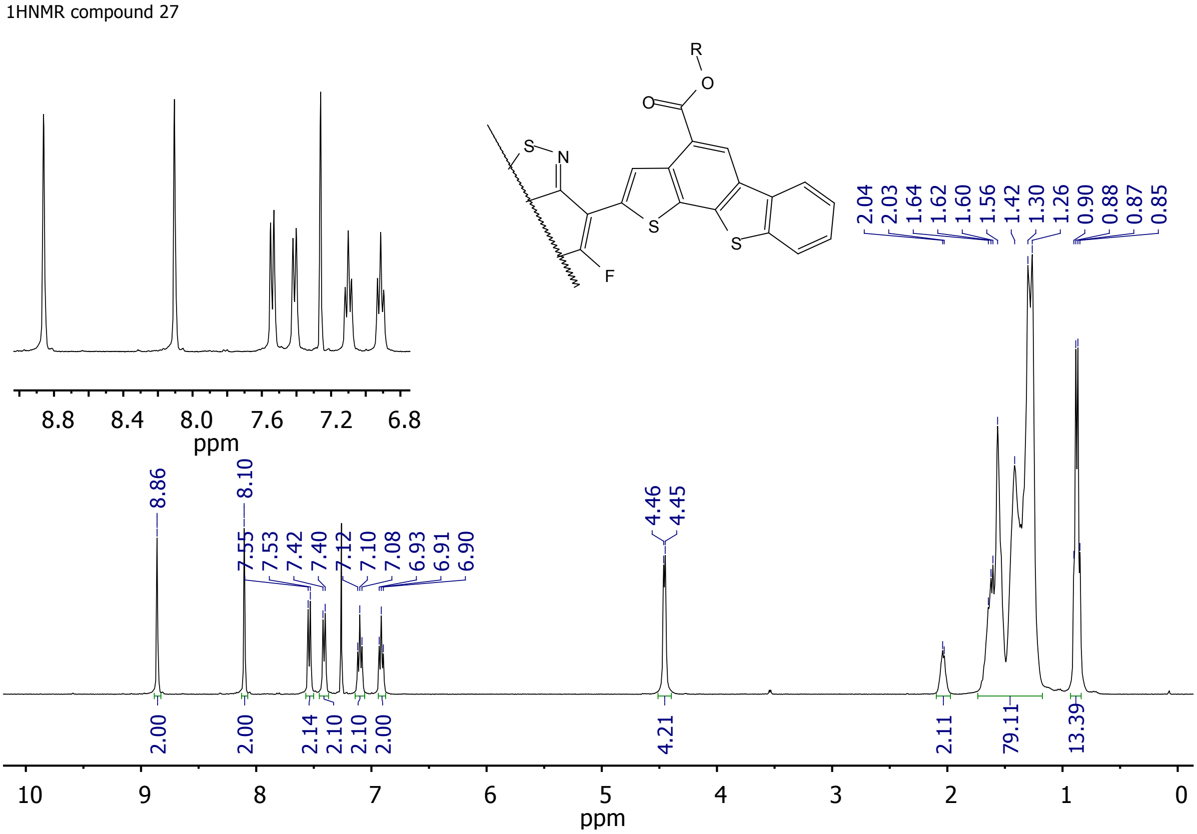

¹⁹F NMR

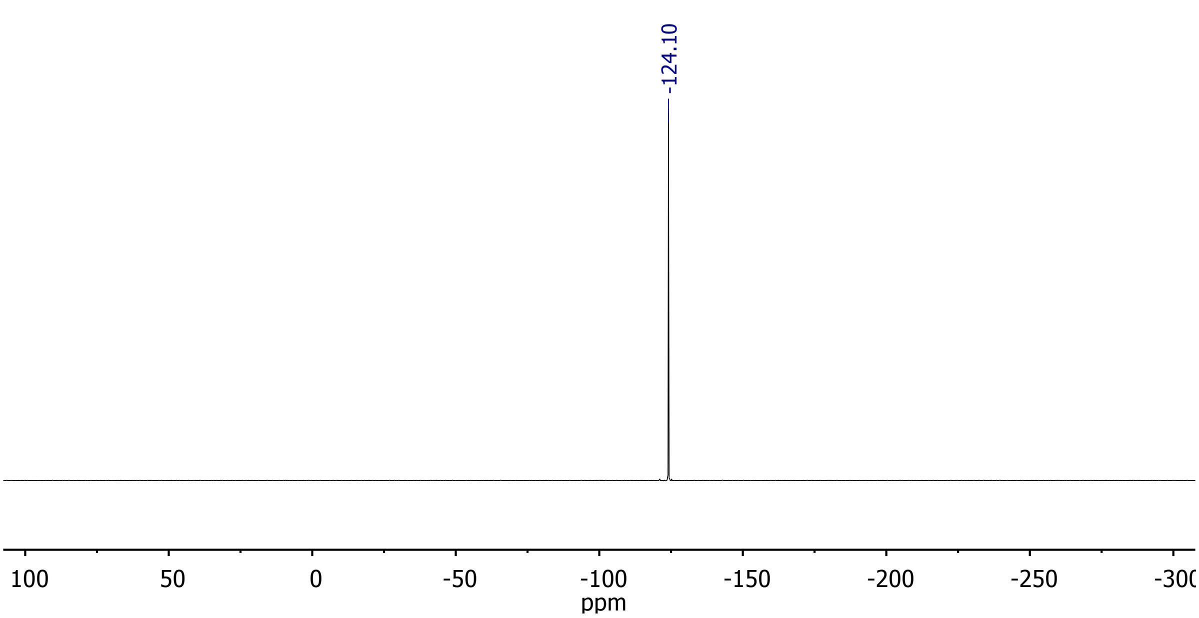

^{13}C NMR (101 MHz, CDCl$_3$)

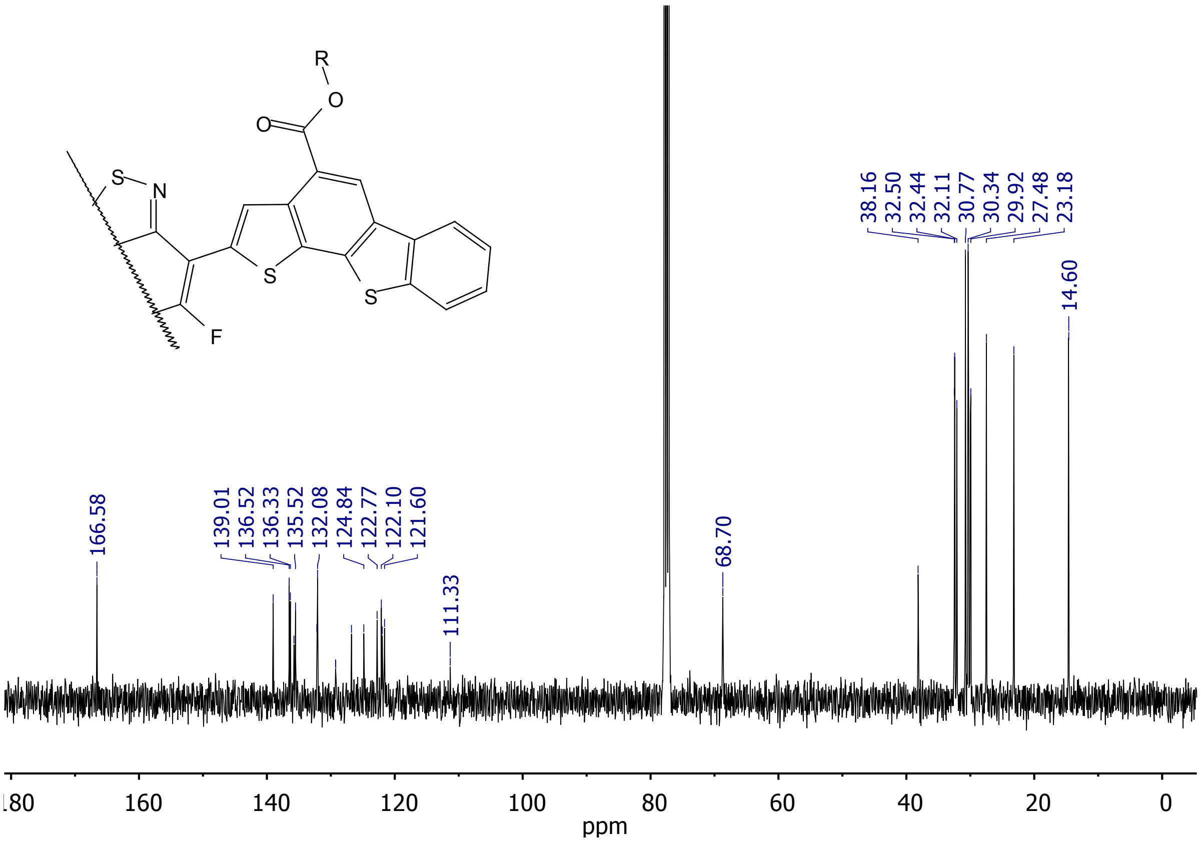

DEPT

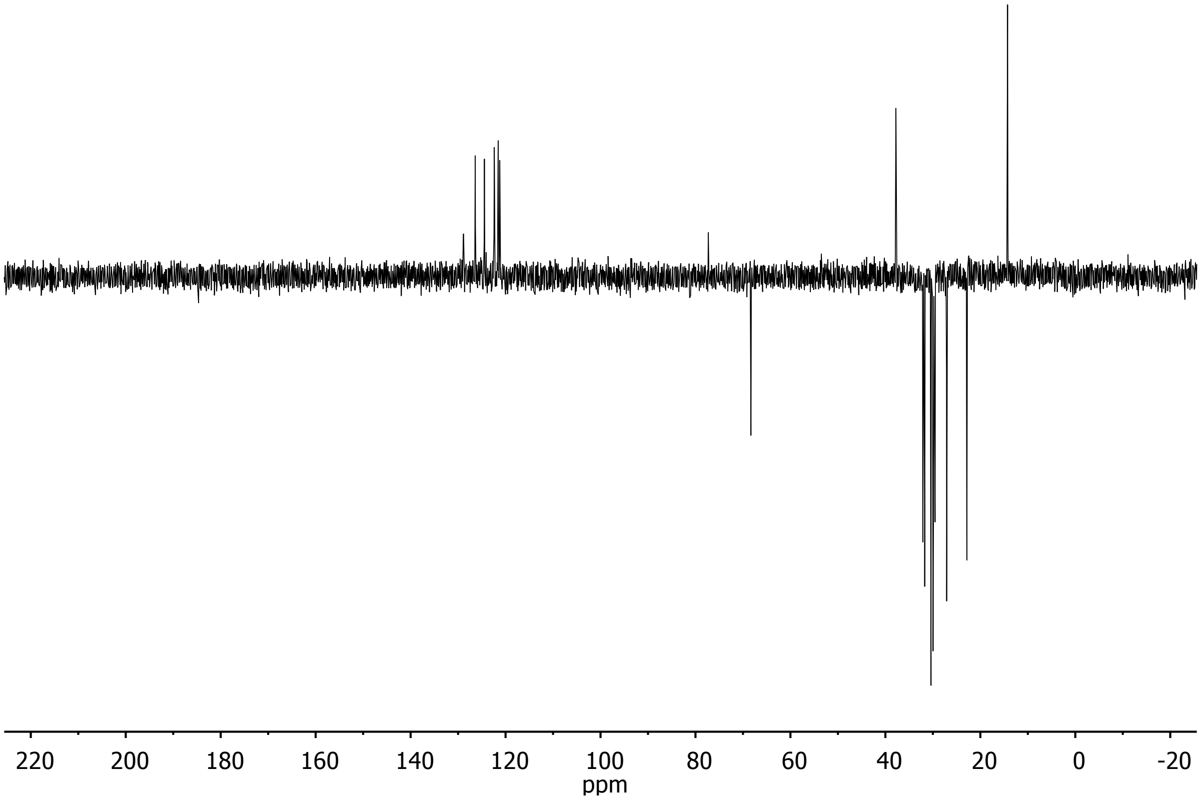

COSY

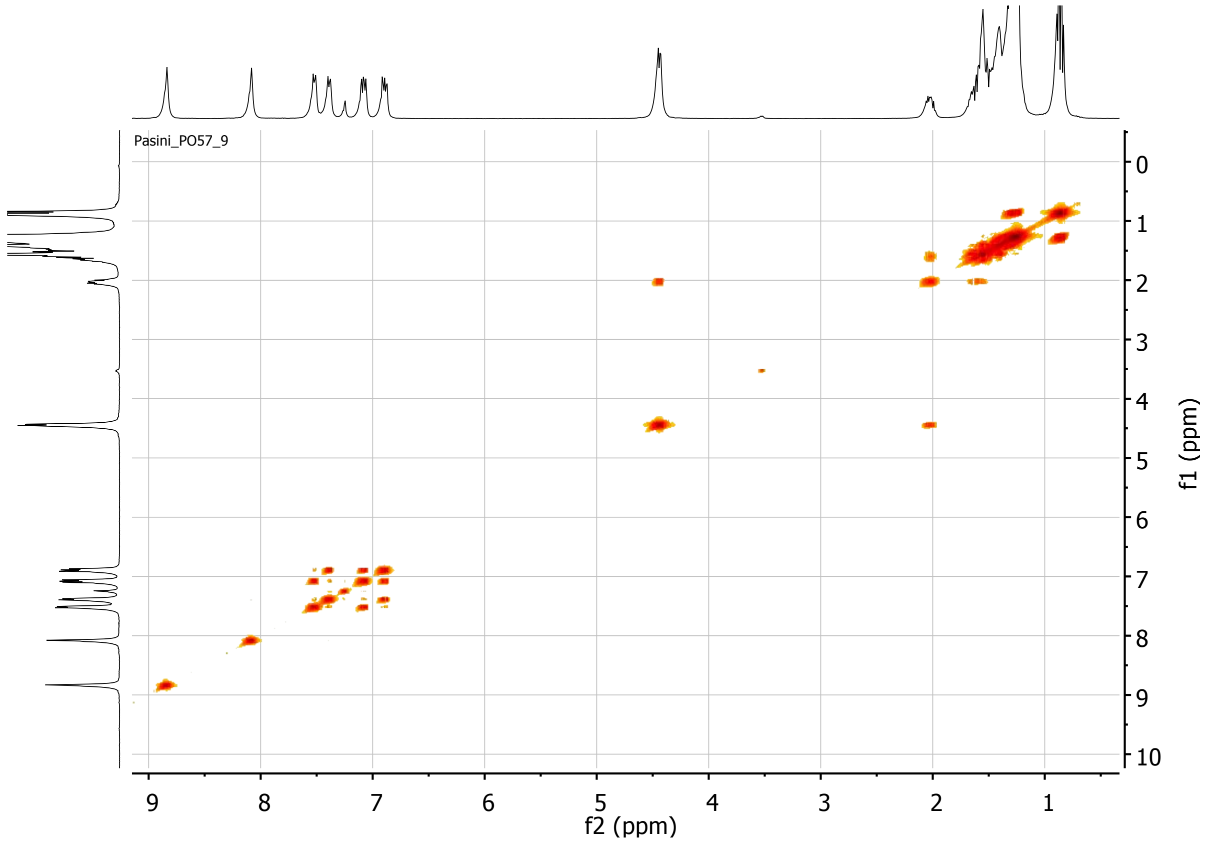

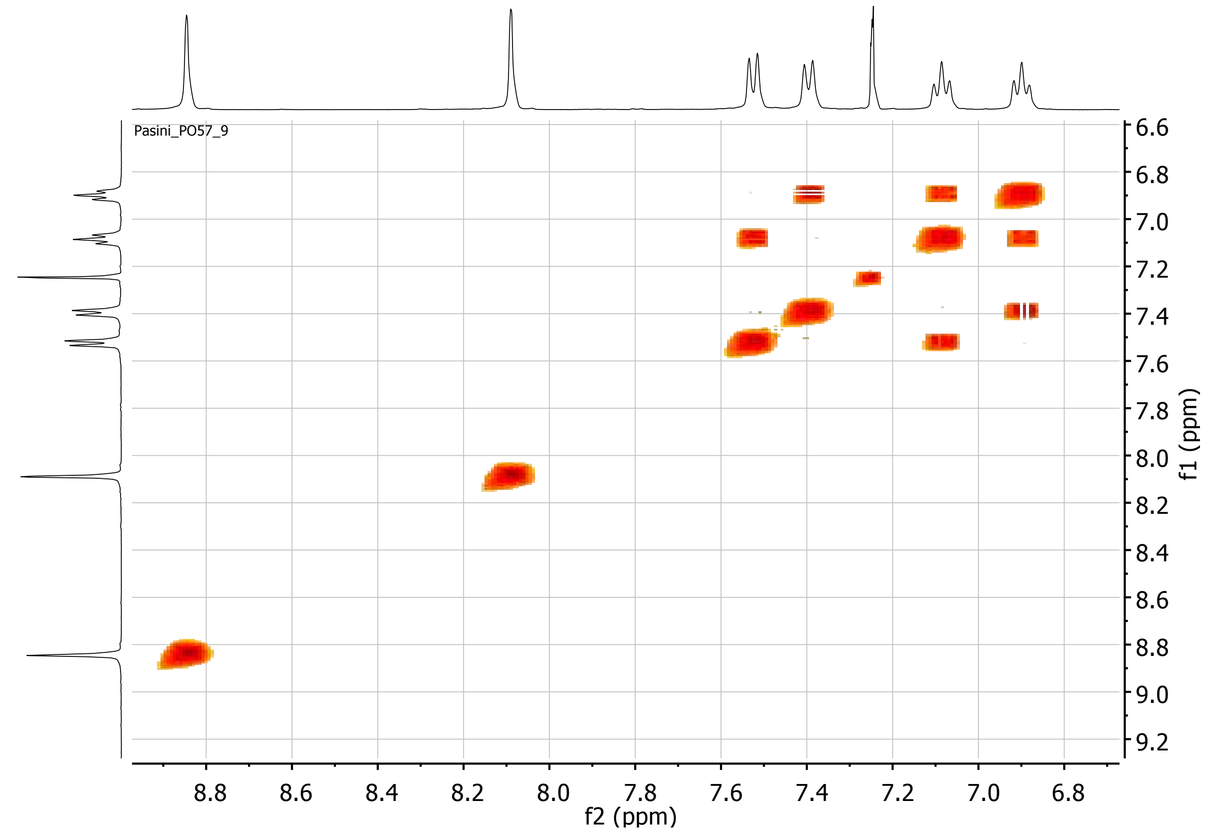

HMQC

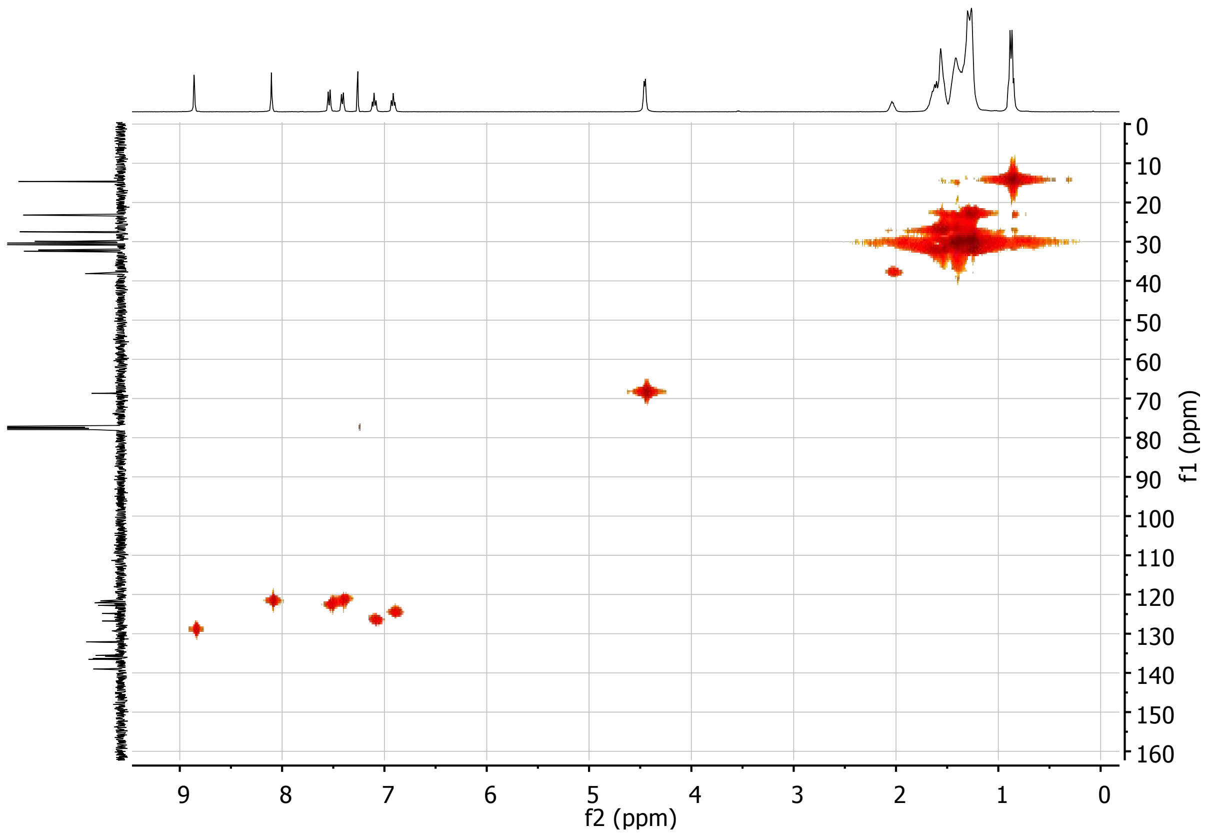

f2 (ppm)
f1 (ppm)

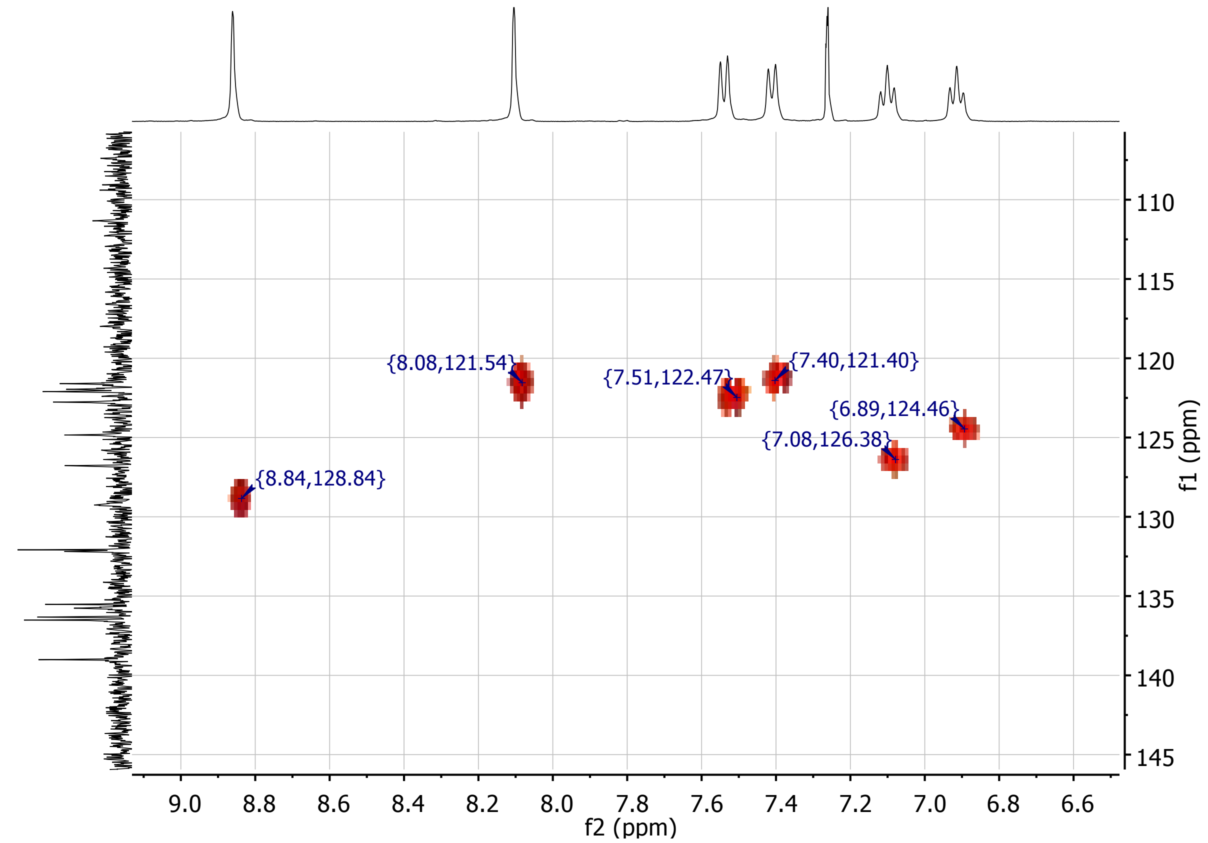

{8.08,121.54}
{7.51,122.47}
{7.40,121.40}
{6.89,124.46}
{7.08,126.38}
{8.84,128.84}
f2 (ppm)
f1 (ppm)

HMBC

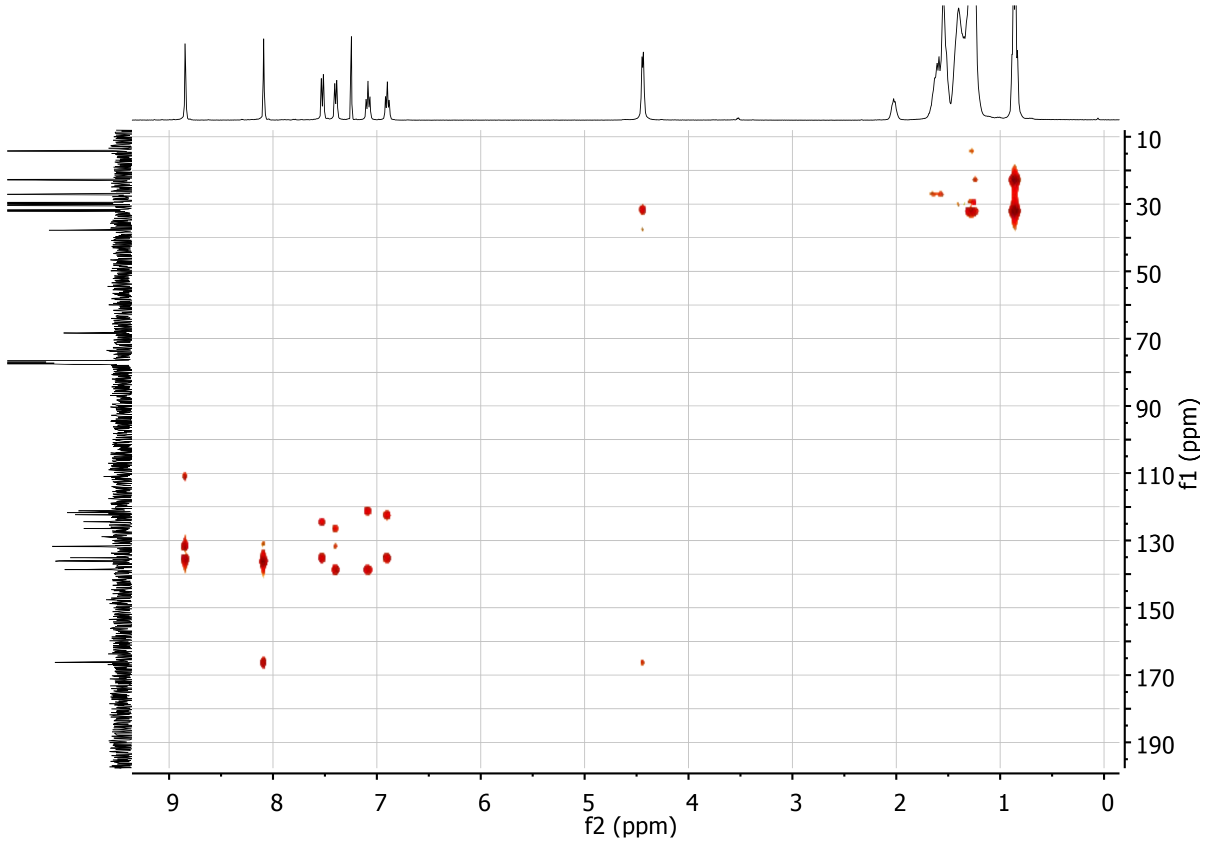

f2 (ppm)
f1 (ppm)

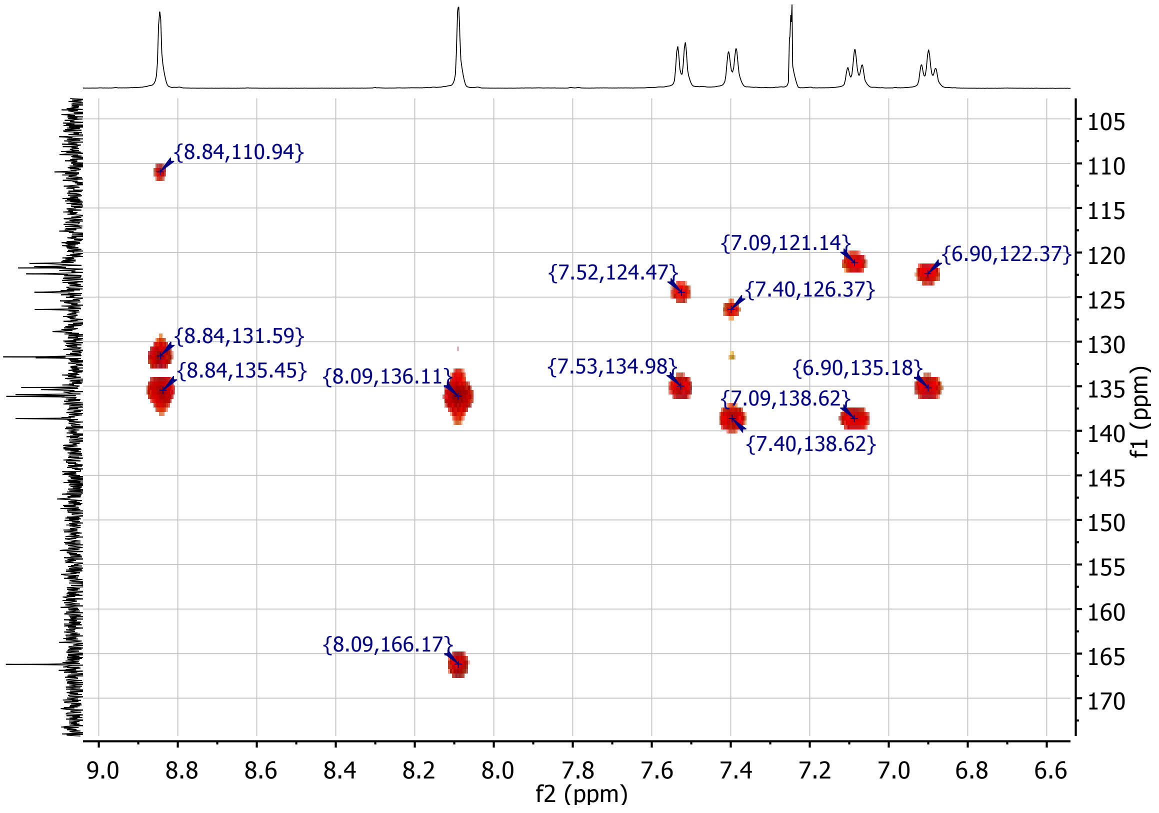

{8.84,110.94}
{7.09,121.14}
{6.90,122.37}
{7.52,124.47}
{7.40,126.37}
{8.84,131.59}
{8.84,135.45}
{8.09,136.11}
{7.53,134.98}
{6.90,135.18}
{7.09,138.62}
{7.40,138.62}
{8.09,166.17}
f2 (ppm)
f1 (ppm)

HRMS

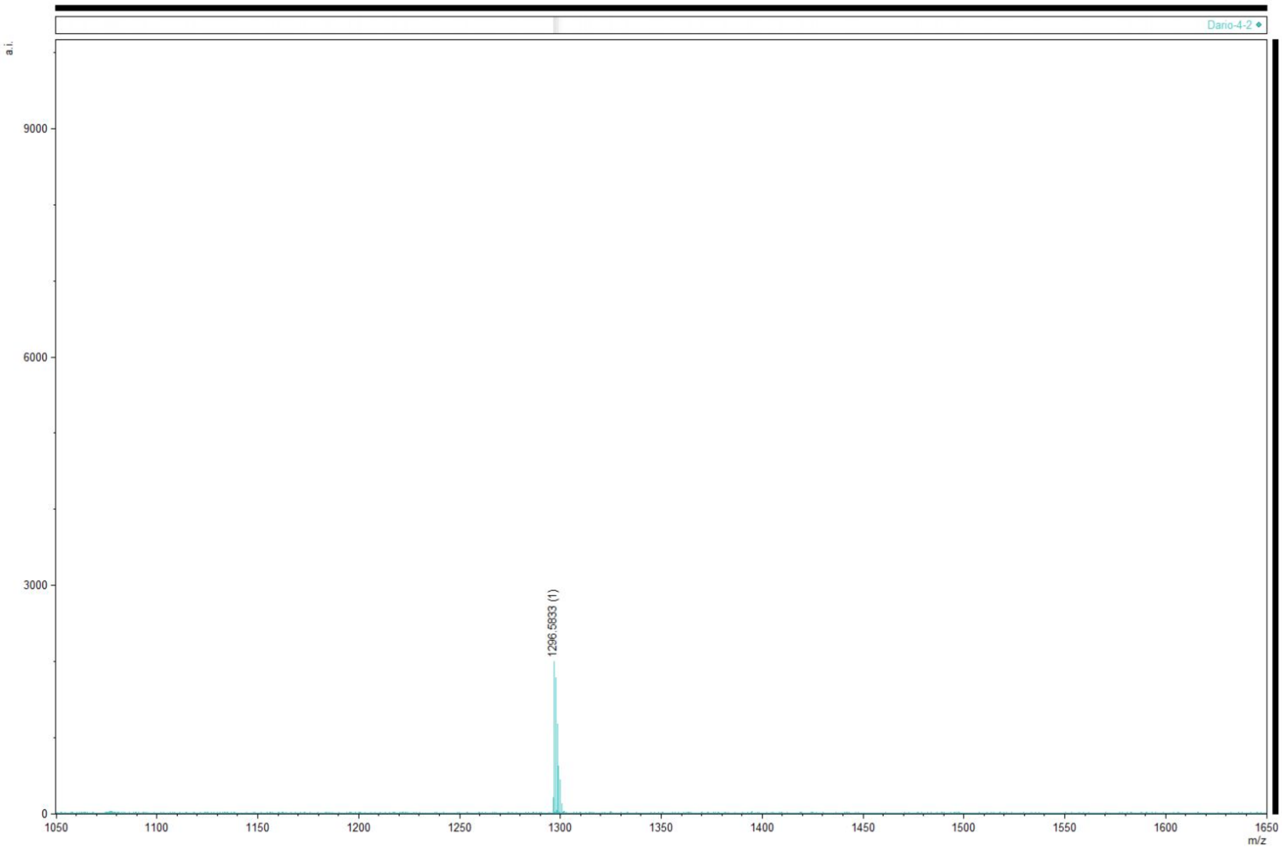

Dario-4-2
a.i.
9000
6000
3000
0
1296.5833 (1)
1050
1100
1150
1200
1250
1300
1350
1400
1450
1500
1550
1600
1650
m/z
